# The molecular athlete: exercise physiology from mechanisms to medals

**DOI:** 10.1152/physrev.00017.2022

**Published:** 2023-01-05

**Authors:** Regula Furrer, John A. Hawley, Christoph Handschin

**Affiliations:** ^1^Biozentrum, University of Basel, Basel, Switzerland; ^2^Exercise and Nutrition Research Program, Mary MacKillop Institute for Health Research, Australian Catholic University, Melbourne, Victoria, Australia

**Keywords:** athlete, endurance training, exercise, resistance training, skeletal muscle

## Abstract

Human skeletal muscle demonstrates remarkable plasticity, adapting to numerous external stimuli including the habitual level of contractile loading. Accordingly, muscle function and exercise capacity encompass a broad spectrum, from inactive individuals with low levels of endurance and strength to elite athletes who produce prodigious performances underpinned by pleiotropic training-induced muscular adaptations. Our current understanding of the signal integration, interpretation, and output coordination of the cellular and molecular mechanisms that govern muscle plasticity across this continuum is incomplete. As such, training methods and their application to elite athletes largely rely on a “trial-and-error” approach, with the experience and practices of successful coaches and athletes often providing the bases for “post hoc” scientific enquiry and research. This review provides a synopsis of the morphological and functional changes along with the molecular mechanisms underlying exercise adaptation to endurance- and resistance-based training. These traits are placed in the context of innate genetic and interindividual differences in exercise capacity and performance, with special consideration given to aging athletes. Collectively, we provide a comprehensive overview of skeletal muscle plasticity in response to different modes of exercise and how such adaptations translate from “molecules to medals.”

## Graphical abstract

**Figure:**
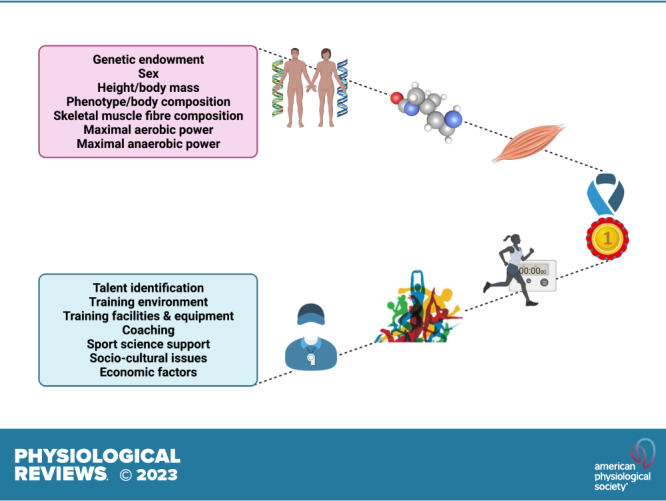

CLINICAL HIGHLIGHTS

1)  During human evolution, *Homo sapiens* emerged as mobile hunters and gatherers, dependent on the natural availability of food. However, today’s sedentary lifestyle and overabundant food availability place a major burden on our metabolic health and are strong drivers underpinning the dramatic rise in noncommunicable diseases.
2)  A sedentary lifestyle, characterized by low maximal oxygen uptake ($\dot{\text{V}}{\text{O}}_{2\max\;}$), unfavorable body composition, and low muscle strength, is an independent risk factor for many chronic diseases and a strong predictor of morbidity and mortality.
3)  Despite marked interindividual differences in the response to standardized exercise training, regular physical activity lowers the risk of and confers therapeutic benefits for many noncommunicable diseases.
4)  Endurance- and resistance-based exercise training protocols confer distinct clinical and health-related benefits and can prevent or reverse many lifestyle-induced metabolic diseases.
5)  Clinical exercise tests based on established, validated physiological outcomes are essential for the diagnosis and subsequent monitoring of clinical conditions.
6)  Investigations of elite human performance provide valuable insights into the molecular, cellular, tissue, and whole body adaptations to extreme metabolic loading. Identification of the mechanisms and pathways that limit exercise capacity may ultimately aid in the identification of novel therapeutic targets to be prescribed to patient populations.

## 1. INTRODUCTION AND BACKGROUND

### 1.1. Historical Context: The Evolution of Human Movement

**FIGURE 1.**
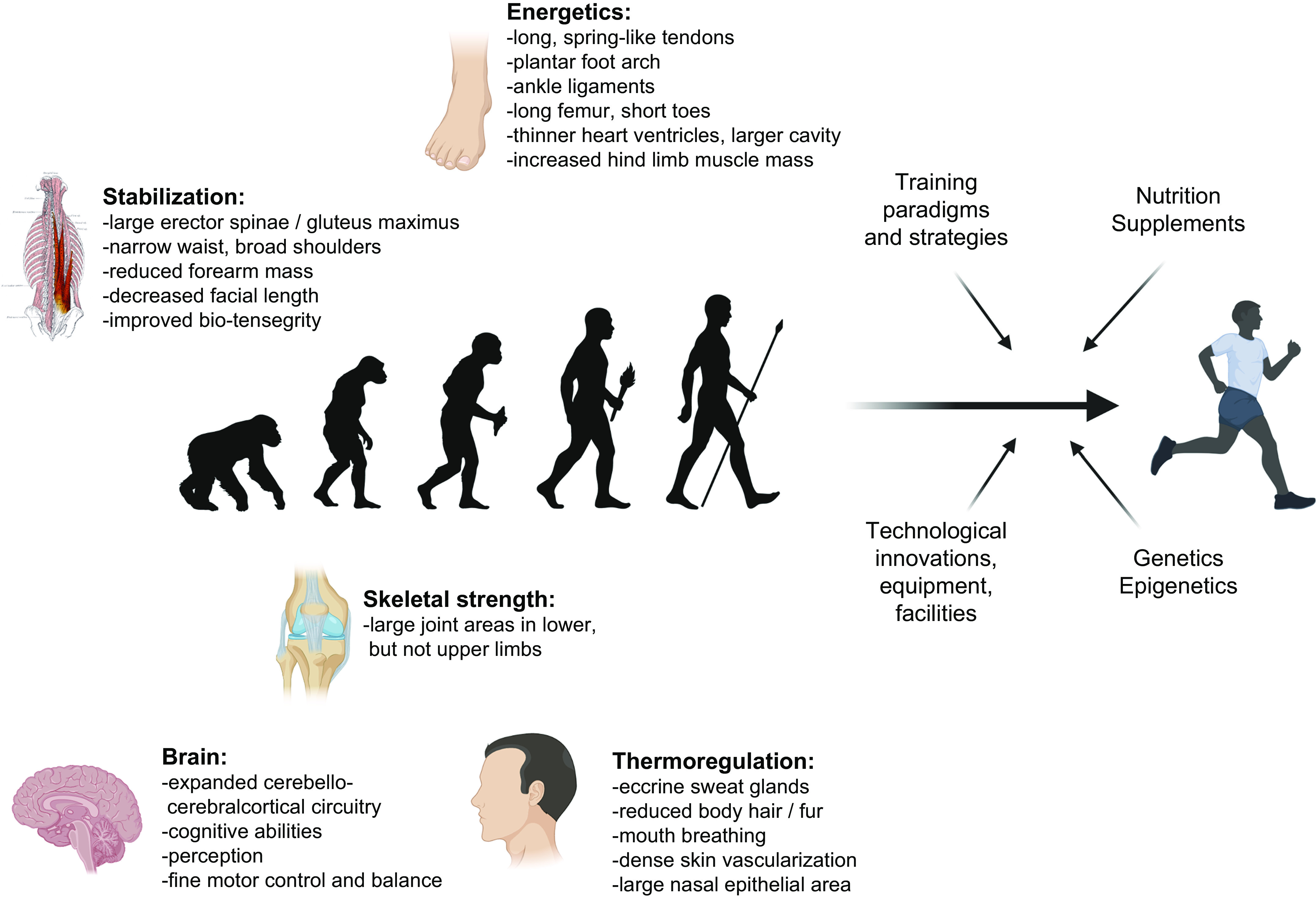
From evolution to modern-day athleticism. Evolutionary selection of 5 main traits has facilitated the prolonged upright, bounding, bipedal locomotion in humans. Energetic barriers are lowered by long, spring-like tendons (in particular the Achilles tendon), the longitudinal plantar foot arch, ankle ligaments, long legs, in particular femur length, and short toes (to increase stride length and reduce vertical trajectories for better locomotor economy), thinner heart ventricles and larger cavity, increased hind limb muscle mass, and other adaptations. Skeletal strength is conferred, e.g., by larger joint areas in lower but not upper limbs to dissipate impact forces. Stabilization for bipedal movement is mediated by large erector spinae and gluteus muscles opposed to reduced forearm mass and an elongated, narrow waist and broad shoulders to facilitate counterrotation of thorax and arms, while decreased facial length helps head stabilization or an integrated system of bio-tensegrity for embedded perturbation repelling. Eccrine sweat glands, with a particular high density in the head for brain cooling, reduced body hair, dense skin vascularization, mouth breathing, and a large nasal epithelial area all contribute to thermoregulation. Finally, coevolution of locomotion and the brain resulted in expanded cerebello-cerebralcortical circuitry (for anticipation, pre-preparation, sensory integration, pre-planned multilevel compensation to deal with perturbations and destabilizations), cognitive capabilities (to recognize landmarks, long-range orientation, recognizing prey and predators, tracking and speculative tracking/anticipation), perception, fine motor control, and balance. See Refs. 1–8 for more information. Modern-day athletic peak performances most likely exceed these general evolutionary traits because of efficient training strategies and paradigms, nutrition and supplements, technological innovations, i.e., pertaining to equipment and facilities, and genetic and epigenetic predispositions. Figure created with BioRender.com, with permission.

The evolution of humankind is inextricably linked to the attainment of an upright, bipedal gait, which conferred an advantage for locomotion, foraging, and recognition of prey and predators (FIGURE 1). Indeed, a superior endurance capacity, coupled with an outstanding ability to thermoregulate, was essential for human survival (1). Evolutionary theory describes the mechanism of natural selection as “survival of the fittest,” the underlying supposition being that the “fit,” as opposed to the “unfit,” had a greater likelihood of survival (9). In this regard, human skeletal muscles, limbs, and the supporting ventilatory, cardiovascular, and metabolic systems were well suited for upright locomotion, with economy of movement for bipedal walking and running far exceeding that of other primates (2–5). Modifications in bone and cartilage structure, larger limbs and joints, and spring-like plantar arches (2, 3), combined with a robust system of perception, fine motor control, and balance, were linked to a larger brain size and associated cognitive sophistication (6–8). The evolution of the larger brain in humans was likely facilitated by the running behavior of our ancestors that enabled the procurement of high-protein sources of food essential for brain development (10). Bipedal, long-distance running not only necessitates complex computation to control gait, balance, and stride but also requires large-scale cognitive processes to recall landmarks associated with abundant sources of food, recognize prey and predators, and enable long-range orientation (10). Such adaptations were supported by adequate energy availability and oxygen supply, coupled with a high degree of metabolic regulation and flexibility (6–8). The superior human proficiencies as hunters, gatherers, and ultimately farmers provided dietary subsistence that enabled the evolution of our energy-costly brain. The coevolution of skeletal muscle and associated organ systems was characterized by progressive and iterative mutual interactions (10). The behavioral lifestyle and energy availability were determined by the periodic cycles of feasts and famines, with certain genes evolving to regulate efficient storage and utilization of endogenous fuel stores, the so-called “thrifty genes” (11, 12).

In contrast to the strong evolutionary pressure to optimize endurance capacity (1), the control of skeletal muscle mass and strength evolved in a more restrained manner. Although adequate muscle strength was closely aligned to the prevailing environmental demands of the day and was indispensable for survival, genes encoding proteins that act on muscle cells to inhibit muscle cell growth, such as myostatin, escaped negative evolutionary selection. This would appear to be somewhat of a paradox, as naturally occurring mutations in the myostatin gene confer several benefits including a substantial increase in muscle mass in mice, dogs, cattle, and even humans (13). In evolutionary terms, however, a lower muscle mass would be associated with a reduction in both resting and locomotive energy expenditure in times of food scarcity, along with the conservation of carbohydrate-based fuels obligatory for preservation of brain function. Excessive muscle mass can also lead to parturition issues (i.e., higher birth weight and larger offspring), predisposing to evolutionary disadvantages (14–16). Non-muscle-related functions of myostatin such as tendon maintenance and repair and injury risk could have contributed to the positive selection of this factor (17). Finally, potential trade-offs between the promotion of fatigue resistance, stamina, and endurance versus muscle mass, strength, and power could have affected the evolutionary process (1). Accordingly, although there exists a certain degree of synergy, distinct control and adaptation to endurance- and strength-based activities have evolved in humans.

### 1.2. Major Themes of This Review

The importance of physical activity for health and well-being was recognized early in human history, dating back to records from 3000 BCE (18). The concept of “exercise is medicine” and the appreciation of athletic prowess were prominent in ancient Greek and Roman civilizations (18). Notably, the evolutionary adaption of humans to a phenotype eminently suitable to the pursuit of long-distance running confers important implications for human health and athletic performance in the present day. Unfortunately, the fundamental link between endurance-based activities and the evolution of numerous human traits has been severely diminished in modern societies in which voluntary physical activity is at an all-time low and has recently been exacerbated by a global pandemic (19, 20). Our twenty-first century lifestyle that in many societies encompasses round-the-clock access to energy-dense, nutrient-poor food in the face of prolonged periods of inactivity has resulted in the proliferation in the rates of diagnosis of several metabolic disease states, a rise in morbidity and mortality, and a high financial burden on health care systems (21). Paradoxically, at the same time, the standard of athletic performance at both the amateur and professional levels continues to advance, indicating a historically unprecedented divergence between the physical capabilities of the great majority of the world’s population of inactive individuals and a small cohort of elite athletes. Indeed, Olympic and/or world championship medalists, world record holders, and athletes achieving within 2% of world-record performance and/or world-leading performance comprise <0.00006% (∼5,000 individuals) of the entire global population of 8 billion (22). In physiological terms, the measure of an individual’s maximal oxygen uptake ($\dot{\text{V}}{\text{O}}_{2\max}$), a marker of aerobic fitness, can be two- to threefold higher in champion endurance athletes than untrained individuals (23). The most striking training-induced adaptations contributing to such differing values are an increased stroke volume of the heart, elevated capillary and mitochondrial density, and a predominance of oxidative “slow-twitch” fibers in the muscles of endurance-trained athletes (24, 25). While a high $\dot{\text{V}}{\text{O}}_{2\max}$ is a prerequisite for successful endurance performance, this measure is also a better predictor of morbidity and mortality than any other established risk factor or biomarker (26–30). Likewise, relative muscle mass (31–33) and strength (34–36) are parameters with high predictive power for overall morbidity and mortality. Clearly, the biology underlying maximal endurance and resistance exercise performance confers advantages beyond the athletic arena (9, 37), and while differences in physiological capacity between elite athletes and sedentary individuals highlight the huge disparity in performance capacity, they also provide insights into the roles of various organ systems and the potential limits to human performance.

The last decade has seen major advances in unraveling many of the putative mechanisms by which cellular, molecular, and biochemical pathways are altered by exercise (9, 18, 38–41). However, many of the adaptations that underpin elite athletic performance remain poorly understood. In particular, the training programs of world-class athletes owe more to tradition and the “trial-and-error” methods of pioneering coaches than exercise biologists or sport scientists. Determining the precise role of exercise intensity, duration, and frequency in acutely modifying various signaling cascades and coordinating specific training-induced physiological adaptations in athletes may offer valuable insights into some of the critical pathways to target in order to fight the battle against inactivity-related diseases in the general population. Not only may sedentary or “at-risk” populations benefit from “personalized” physical activity-based interventions to prevent and treat chronic lifestyle-induced pathologies (42–44), but mechanistic insights could reveal targets for novel pharmacological interventions (45–47). A better understanding of the molecular mechanisms that control skeletal muscle cell plasticity may also provide a stimulus for further improvements in elite athletic performance (48, 49). The fastest 100 m sprint by a male athlete under 18 (10.31 s, Brume Okeoghene, June 17, 2021) would have won the gold medal at the 1980 Olympics, whereas Usain Bolt’s 100 m world record of 9.58 s in 2009 far exceeded predictive statistical models at that time (50). In recent years, much scientific debate has been focused on the limits to the men’s marathon (42.195 km) (51–54). Changes in both the culture of sport and the recognition of modern sports science research have supported emerging activities in which “barriers” to performance have been tackled as science-driven endeavors (55). The “sub-2 hour marathon project” is an example: the course design, ambient temperature, humidity, wind, elevation above sea level, and comprehensive use of pacemakers in highly choreographed formations helped Kenyan runner Eliud Kipchoge run 1:59:40.2 in a specially paced time trial in October 2019 (53, 56). Likewise, there have also been substantial advances in world-best performances by female and masters-level athletes during this time (57–59).

**FIGURE 2.**
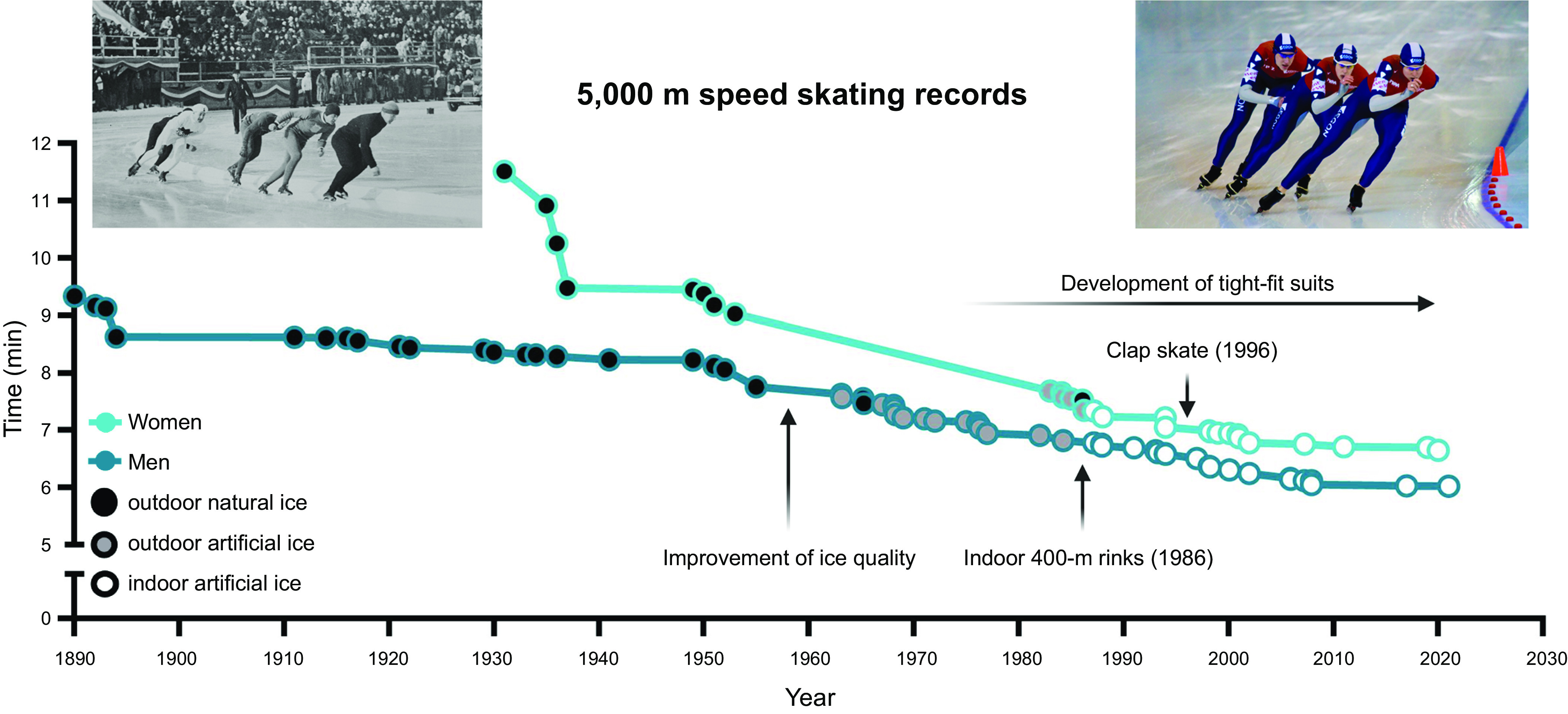
Innovations that contributed to the progress in the development of world records over time (light blue for women, dark blue for men). Speed skating is one of many cases in which the progression of world records is driven by innovations (63). For example, the invention to improve ice quality (natural vs artificial ice) by refrigerated ovals (first 1958), spraying tiny droplets of water to smoothen the surface (first 1960), followed by the ice resurfacer “Zamboni” (Olympics 1960) and eventually indoor rinks all contributed to new records. Additionally, the development of gear such as tight-fit suits to improve aerodynamics and the invention of the clap skate that enabled a longer contact with the ice as well as further enhanced aerodynamics due to the crouched posture pushed the progress in world record development (http://www.speedskatingstats.com/index.php?file=records). Image on *left* was taken at the 1932 Winter Olympics and is from Henriksen & Steen (public domain, via Wikimedia Commons); image on *right* was originally posted to Flickr by adrian8_8.

**FIGURE 3.**
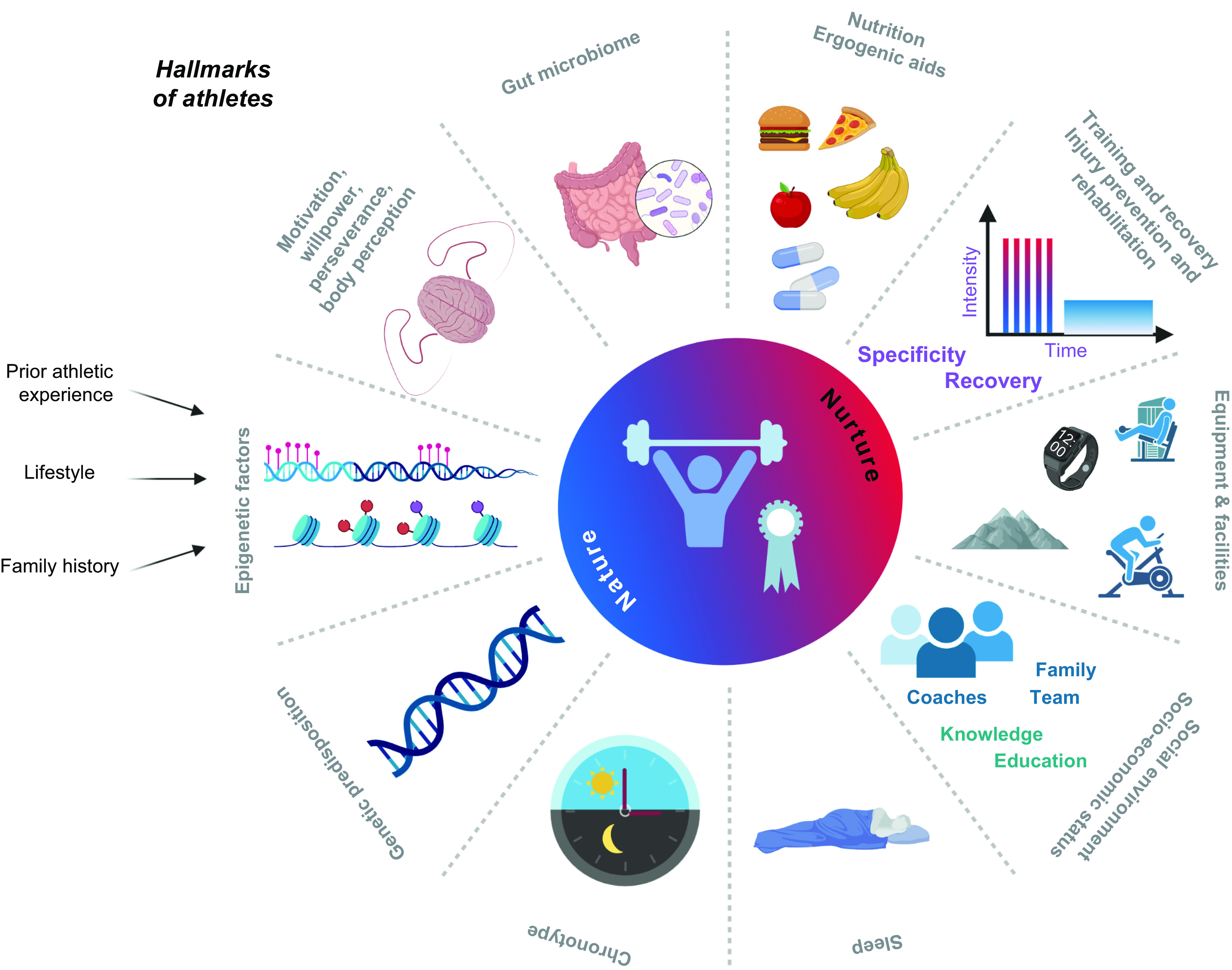
Elite athletic performance is determined by the complex interaction of intrinsic and extrinsic factors. Undisputedly, genetic predisposition, even though poorly defined and understood, contributes to athletic prowess and trainability. In fact, the “right” genes might even be a prerequisite for elite, world-class performance. The epigenetic landscape is at least in part inherited but, in contrast to the genome, can also be influenced by behavior, including prior athletic experience, nutrition, and other lifestyle factors. A higher-than-average motivation and drive, the willpower to overcome obstacles, adversities, and setbacks, perseverance, and the willingness to forgo activities common for non-athletic peers are essential. These factors as well as daily training are shaped by body perception and prior athletic experience, including a multidisciplinary/multisport practice in youths. Most likely, nutrition, ergogenic aids, and gut microbiomes mutually interact in an intimate manner, collectively affecting trainability and performance. Optimal training strategies not only comprise personalized planning but should also integrate adequate consideration of recovery and injury prevention and, if the situation arises, rehabilitation. State-of-the-art equipment and facilities are part of a permissive environment, which is also strongly shaped by socio-economic status and social interactions with coaches, medical and other staff, team members, parents, siblings, friends, and rivals. This network of supporting people helps to optimize knowledge and education for proper planning and implementation. Finally, peak performance also relies on proper and personalized sleep patterns, matched to the individual chronotype. The use of doping might confer performance enhancements in the short term but is linked to long-term health detriments and is counter to the ethos of a fair and clean sport. Figure created with BioRender.com, with permission.

Technological innovations in sport now drive performance enhancements at the elite/professional level, as witnessed in track and field (60), swimming (61), cycling (62), and speed skating (FIGURE 2) (63), with such advances filtering down to amateur athletes, epitomized by the widespread access to new footwear that improves running economy (64, 65). The use of novel technologies, such as fitness trackers, step counters in cell phones, or other wearables, reveals behavioral aspects of physical activity linked to performance outcomes at both a recreational and an elite level. Such technologies can inform training design as well as the impact of specific interventions on health and performance outcomes (66). In the final analysis, however, progress in athletic performance is multifactorial, encompassing gene-environment interactions (67–70), advances in infrastructure, training paradigms, and design (71), nutrition and ergogenic aids (72, 73), as well as techniques facilitating recovery and regeneration, social and economic factors, prior athletic experience and physical activity background (74), and, in an unknown number of athletes, the use of sophisticated doping strategies (FIGURE 3) (58, 75). The range in performance capabilities, the ongoing improvements in athletic records, and the accomplishments of older individuals at the masters level (57) in octogenarians (76) or even centenarians (77) allude to the vast continuum of the adaptive response of muscle tissue and other organs to a sedentary lifestyle or exercise training. In this review, we provide a synopsis of the training strategies of elite athletes, the bidirectional dialogue between science, coaches, and athletes in training design and implementation, and the inherent and acquired differences between world-class athletes and the general population (sect. 2). Such concepts are linked to a discussion of the cellular, morphological, and functional training-induced adaptations in athletes (sect. 3). In sect. 4, our understanding of the molecular mechanisms that underpin the responses to acute exercise is outlined, although these insights have largely been obtained in non-athletes and/or animal models and their translation to elite performance remains to be validated (78). In contrast to several previous reviews, we address these issues for both endurance- and resistance-based exercise training. Wherever possible, direct links between training strategy, cellular adaptation, and molecular mechanisms are discussed in an attempt to integrate these features. Finally, we provide a discussion on whether all individuals can become gold medal athletes (sect. 5).

## 2. OPTIMIZING TRAINING ADAPTATIONS TO ENHANCE ATHLETIC PERFORMANCE

### 2.1. Principles of Exercise Training: Specificity, Progressive Overload, Reversibility

**FIGURE 4.**
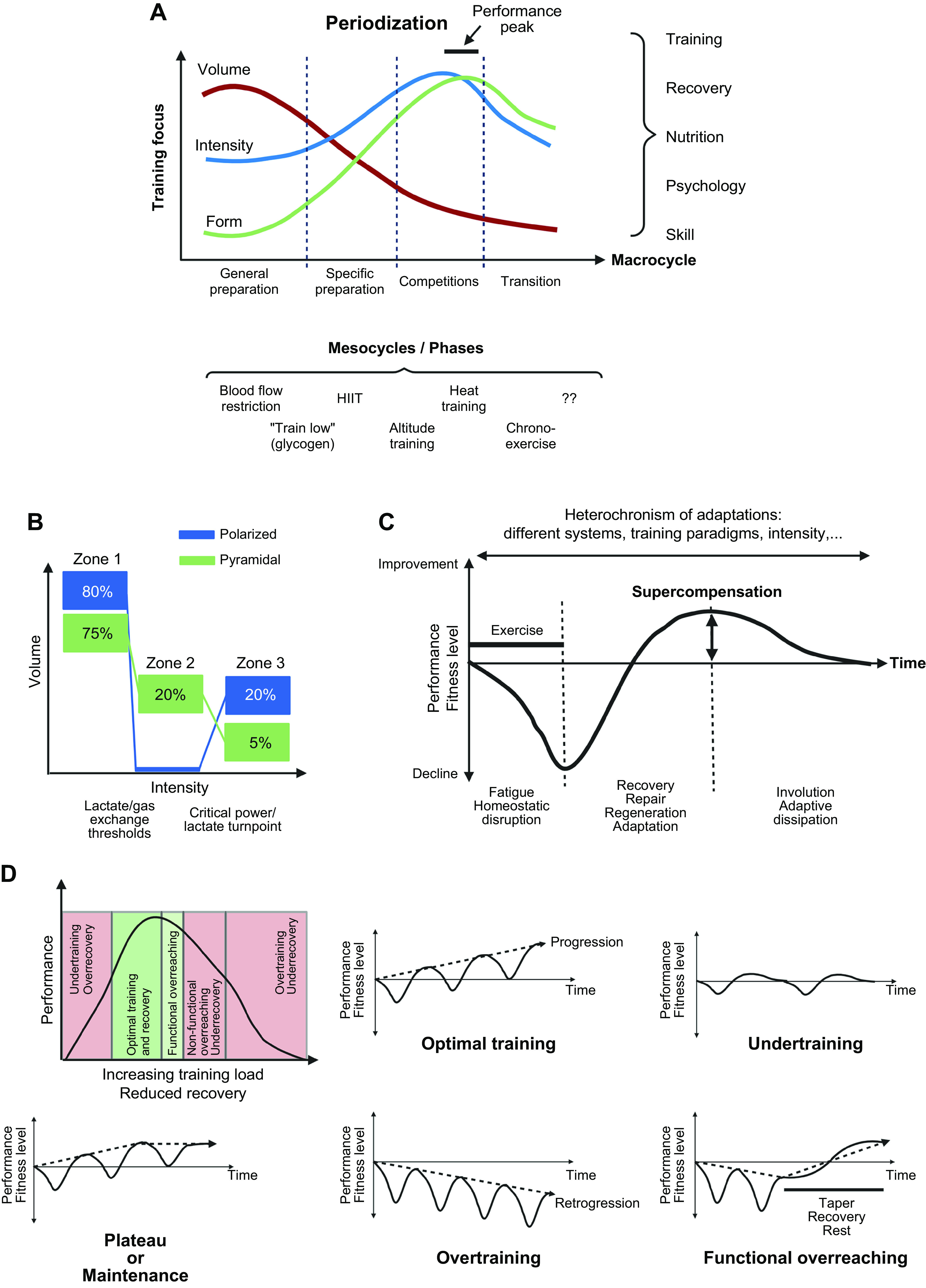
Training principles and strategies. *A*: to achieve peak performance at the time of competition, training volume, intensity, and form/specificity have to be adapted in different cycles/phases. Specific paradigms, e.g., high-intensity interval training (HIIT), “train low,” and others, are likewise periodized and matched to the prevailing volume/intensity/form requirements. Importantly, the periodization of training has to be matched to that of nutrition (e.g., low glucose vs. carb loading), recovery, psychological aspects, and skill acquisition. *B*: within shorter cycles, e.g., weekly planning, polarized or pyramidal partitioning of training volume at different intensities (e.g., defined by lactate/ventilatory thresholds between *zones 1* and *2* and the critical power/lactate turnpoint between *zones 2* and *3*) helps to improve performance and reduce overtraining. *C*: training adaptation is initiated by a homeostatic disruption induced by exercise. After exercise cessation, recovery and repair mechanisms not only result in a return to baseline but trigger adaptive mechanisms, optimally in a supercompensatory manner, which should help to protect muscle better from future perturbations. However, in the absence of continued stimuli, i.e., detraining, this supercompensatory response is abolished by an adaptive dissipation. The amplitude and temporal aspects of this curve are strongly influenced by the training paradigm and related parameters. Moreover, within the same system, biochemical processes, cell types, or tissues can react in a different manner (heterochronism of adaptation). *D*: performance gains are controlled by the balance between training load and recovery. A suboptimal planning can result in either undertraining with little or no gains or overtraining, in which performance decreases (retrogression) and the risk for injuries increases. In proper conditions, a functional overreach helps to maximize progression and overcome training plateaus. Figure created with BioRender.com, with permission.

A reductionist view of training for elite sport performance identifies a range of interdependent adaptations that enable an athlete to sustain the highest rate of energy production for the duration of their event, optimize economy of motion, defend cellular homeostasis, and delay the onset of fatigue (9, 54, 55, 79, 80). In addition to undertaking workouts that promote these adaptations, an athlete needs to attain the optimal physique and technical skills specific to their event(s). To achieve these goals, elite athletes engage in periodized training techniques involving long-term systematic planning for major events and undertake prolonged, intense workouts fueled by optimal nutritional practices, while building resilience against illness and injury (55, 81, 82). Coaches integrate a series of workouts that individually target important competition performance traits into a periodized training program composed of short (7–21 days) microcycles and longer (3–8 wk) mesocycles, culminating in targeted competition peaks within a season or year (macrocycle) (FIGURE 4). There is a firm belief that the training-induced changes in skeletal muscle resulting from the high-volume, high-intensity training undertaken by elite athletes over several years is largely responsible for the observed improvements in performance over time.

Elite athletes present a narrow range of values in many morphological, biomechanical, physiological, metabolic, perceptual, psychological, and other traits, depending on their specialized event (83). Although there are multiple and varied approaches to optimize adaptation to enhance sporting performance based on a multitude of mechano-biological descriptors, the general principles of exercise training focus on three main concepts: progressive overload, specificity, and reversibility. These principles of training can be applied to individuals with a wide range of abilities because the physiological response to specific stimuli is largely predictable. However, the magnitude of response of one athlete to a standardized training protocol may differ substantially from that of another because of innate genetic predisposition, environmental factors, access to training facilities and sport science support, socio-cultural and economic factors, and the interactions between these components (FIGURE 3). The question of whether all individuals respond to exercise training (i.e., demonstrate a measurable improvement in a specified physiological outcome measure) is discussed subsequently.

The principle of progressive overload states that once an athlete has adapted to a given training load, the subsequent training stimuli must be progressively increased to perturb the homeostasis and thereby promote further adaptation (FIGURE 4). Overload can be quantified according to the volume of training (how much), the intensity (how hard), and the frequency (how often), with the magnitude of adaptation dependent on the interaction between these variables, the prevailing fitness level of the athlete, and their genetic ceiling. In addition, cellular and whole body homeostasis can be amplified by exposure to altitude, heat, or altered fuel availability (55). Such approaches are based on the premise that by imposing greater “metabolic stress” and provoking extreme disturbances to homeostasis, intracellular responses in skeletal muscle (and other tissues and organs) will be maximized, thereby invoking superior training adaptation and enhancing one (or more) of the factors underpinning performance (84). Several training strategies are currently practiced by competitive athletes in the belief that they amplify adaptation and lead to improved performance capabilities. Here, we describe a selection of training strategies that have high uptake by elite athletes and have plausible biological mechanisms that might explain current practices (85–87).

The principle of specificity states that any training-induced adaptations that accrue to an athlete are unique to the type of exercise mode performed; this is most evident when contrasting the divergent phenotypes that result after undertaking either prolonged endurance- or strength/resistance-based training (86, 88). The principle of specificity states that the closer the training routine is to the requirements of competition, the greater the likelihood of successful outcomes. For this reason, the foundation of any training program should reflect the desired training adaptation necessary to enhance sports-specific performance. The principle of specificity should operate with regard to not only the modality of training but also the intensity and speed/power output at which an athlete performs training (discussed below). The principle of reversibility states that there will be a decline or complete loss of training-induced adaptations when an athlete reduces or stops training for a substantial time (i.e., several weeks up to several months). Reductions in both training volume and intensity diminish many of the adaptations that accrue from daily or twice-a-day training, with such a response leading to concomitant performance decrements. The time courses of loss of adaptations after both well-trained endurance athletes and recreational sportspersons stop daily training are rapid: declines in whole body maximal and submaximal responses to exercise occur during the first 7–21 days of inactivity, becoming somewhat stable after 2 mo of detraining (89–93). In athletes who predominantly train for strength and power, and depending on the type of strength test performed, there is a limited decline in muscular strength during short-term (up to 21 days) inactivity, but decay rates increase substantially after 4 wk and longer (89, 93–95). It is important to highlight that the principle of reversibility differs from a competitive “taper” before a major event/competition: during a taper, the volume and frequency of training are deliberately reduced but the intensity is maintained or even increased, resulting in a performance enhancement of 1–2% (96, 97).

### 2.2. Intensity vs. Volume to Optimize Training Adaptation

#### 2.2.1. High-intensity, low-volume vs. low-intensity, high-volume training to maximize endurance training adaptation.

Recently, there has been renewed scientific inquiry along with widespread public interest in the potential for high-intensity interval training (HIIT) to induce physiological adaptations that are similar or even superior to a traditional, continuous endurance-based exercise prescription for health and performance (98–100). HIIT broadly refers to exercise that is characterized by relatively short bursts of vigorous activity interspersed by periods of rest or low-intensity exercise for recovery. A common classification subdivides this type of training into *1*) sprint interval training [SIT, supramaximal efforts performed at power outputs/speeds > peak oxygen uptake ($\dot{\text{V}}{\text{O}}_{2\mathrm{peak}}$), for 30–60 s, with 1- to 3-min rest or active recovery], *2*) high‐intensity interval training (HIIT, comprising near-maximal efforts performed at the power output/speed that elicits $\dot{\text{V}}{\text{O}}_{2\mathrm{peak}}$ for 1–4 min, with 1- to 2-min rest or active recovery), and *3*) moderate-intensity continuous interval training (performed at power output/speed that elicits between 85% and 90% of $\dot{\text{V}}{\text{O}}_{2\mathrm{peak}}$ performed for 5–10 min, with 1-min rest or active recovery). In untrained and recreationally active individuals, both short-term SIT and HIIT are potent stimuli to induce physiological remodeling similar to that attained after traditional prolonged endurance training, despite markedly lower total exercise volume and training time commitment (101, 102).

The notion that interval training is a new, groundbreaking scientific approach to physical conditioning, especially for athletic performance, needs to be placed in historical context. Coaches and athletes have appreciated the value of this form of training since the early twentieth century, with many notable cases in which a range of different work to rest intervals were trialed, tested, and refined to prepare for competition (99). Interval training was widely used by a Finnish coach, Lauri Pikhala, who worked with many champion runners including Paavo Nurmi and Hannes Kolehmainen. Between 1920 and 1930, Nurmi was the most dominant distance runner in the world, winning a total of nine Olympic gold medals. The foundation of Pikhala’s training methods focused on running a high number of repetitions (20–30 efforts) at close to the athlete’s race pace interspersed with short (<60 s) rest intervals. Subsequently a German physician and coach, Woldemar Gerschler, working with cardiologist Herbert Reindel, fine-tuned a similar interval training approach focusing on the manipulation of the work:recovery periods, based on an athlete’s heart rate. An athlete would run over a distance fast enough to elicit a heart rate close to 180 beats/min, after which they rested until the heart rate dropped to ∼120 beats/min; at this time, the next work bout was performed. Gerschler and Reindel proposed that the rest or recovery interval was the most important aspect of their approach because it was during this phase that the heart adapted, allowing it to grow larger and stronger (99). In the 1960s, the New Zealand running coach Arthur Lydiard advocated a shift away from high-intensity interval-based training to high-volume, continuous training for endurance performance. Lydiard advocated running as much as 160 km/wk during the preseason conditioning or “base” phase, with both middle- and long-distance runners undertaking similar volumes of work (103). Although there was a perception that such a high volume of training could only be performed at low intensities (i.e., high volume, low intensity), this was not the case: running during this phase of conditioning was prescribed at speeds that corresponded to an athlete’s best 16 km race pace (for middle-distance athletes) or best marathon pace (for long-distance runners). This conditioning phase could last from as short as 8 wk to half a year. Lydiard’s athletes had major success over two Olympiads (Rome 1960 and Tokyo 1964), winning medals across a wide range of distances including triple Olympic gold medalist Peter Snell (800 and 1,500 m), John Davies (bronze medal 1,500 m), Murray Halberg (gold medal 5,000 m), and Barry Magee (bronze medal, marathon).

Despite these successful coach-driven approaches to conditioning for elite athletes, it was not until the 1960s that the first scientific publications on the physiological bases of training for human performance appeared, and even today the scientific literature on the unique effects of specific training interventions on the performance of highly trained athletes is sparse. Indeed, although the foundation of all training programs for the enhancement of sport performance is the volume, intensity, and frequency of exercise, the relative importance of these interdependent variables has not been established for many of the key physiological adaptations to training, nor their impact on performance outcomes (104, 105). This is because training prescription is infinitely variable, with countless permutations around the core tenets of the general principles of training (FIGURE 4). Adding to the complexity of training prescription is the multiplicity of the physiological/technical demands of many athletic events, with many requiring components of both endurance and strength/power, as well as different modes of exercise (i.e., swimming, cycling, and running in the triathlon). Potential “interference effects” between endurance- and strength/power-based training regimens are discussed below.

There has been spirited scientific debate as to whether training volume or training intensity promotes the greatest adaptation in skeletal muscle (104, 105), with this dialogue focusing predominantly on exercise-induced changes in mitochondrial content, typically assessed by quantifying the maximal activity of citrate synthase, the first step of the tricarboxylic acid cycle, or skeletal muscle respiratory capacity (see sects. 3.4.1 and 4.5). Although higher intensities of exercise generally elicit greater increases in mitochondrial content than lower exercise intensities per unit of time or work (104), such a narrow perspective ignores any functional outcomes, such as athletic performance. Perhaps more to the point, the data used to support one or the other position (i.e., volume vs. intensity overload) have come from studies that employed untrained or recreationally active subjects participating in short-term interventions (2–6 wk) undertaking one-dimensional training programs consisting of either HIT or continuous, submaximal endurance-based training. It is not clear how these results can be extrapolated to elite athletes with a prolonged history of periodized training that includes a variety of workouts with different goals, performed within well-defined training cycles, at volumes, frequencies, and absolute exercise intensities/power outputs that far exceed those capable of being attained by their less genetically gifted counterparts. In this regard, a recent study reported reductions in mitochondrial respiration in skeletal muscle in response to 4 wk of intensified HIT in moderately trained individuals (106). The impairment in mitochondrial function occurred during the week of heaviest training load but was dissociated from both mitochondrial activity and mitochondrial protein abundance, which both peaked at that time (106). Despite the transient impairments to mitochondrial respiration, performance parameters all increased after the intensified HIT regimen. Furthermore, the training undertaken by the participants in that study consisted exclusively of maximal HIT (106) and can only be tolerated by highly trained athletes for more than a few successive days (107).

**FIGURE 5.**
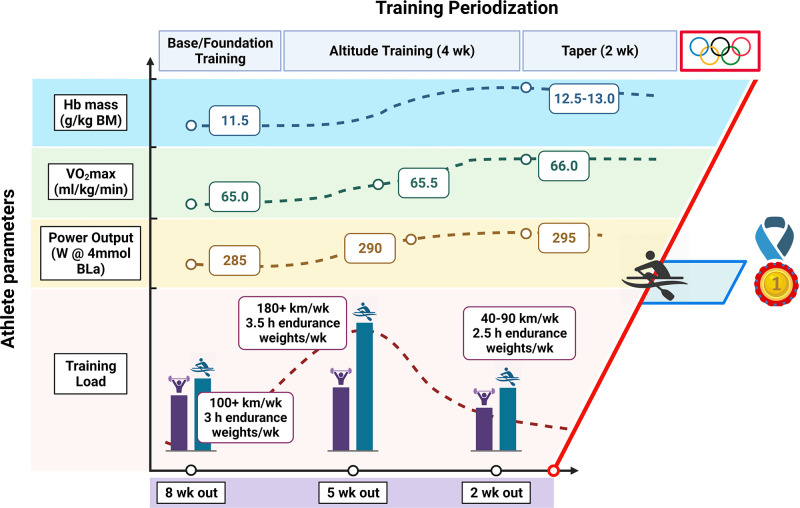
Periodization of training for an elite athlete. Schematic representation of periodization of training along with physiological data collected during preparation for the 2016 Rio Olympic Games for a gold medal-winning female rower. BLa, blood lactate; BM, body mass. See glossary for other abbreviations. Figure created with BioRender.com, with permission.

Since the classic model of training periodization was first proposed over four decades ago (108), there has been widespread discussion about how best to implement training stimuli to optimize adaptation and athletic performance (109). Although several long-term periodization approaches have been described (110), controlled studies comparing the impact of these different protocols on performance outcomes are lacking. As noted, anecdotal testimonies from top athletes and their coaches (111), case studies of elite performers (112, 113), and reports of small cohorts of top athletes from specific sports (53, 114, 115) provide insights into the training practices of elite performers, but such studies merely document what successful athletes did; they do not reveal what made those athletes successful or prove that the program they followed was optimal (116). Indeed, there may have been many athletes who followed similar programs who were not successful, fell ill, suffered injury, or dropped out of the sport completely. Notwithstanding these limitations, detailed analyses of the training methods of elite athletes enable sport scientists to examine relationships between training inputs and variables directly or indirectly related to performance (FIGURE 5). This information can also provide a basis for hypothesis testing with respect to training load and physiological adaptation. There have been multiple empirical descriptions of the distribution of training intensity in highly trained/elite athletes competing in endurance-based sports (110, 114, 117–121). Depending on the specific loading characteristics of the sport (i.e., weight bearing vs. non-weight bearing), international athletes competing in endurance events typically train for between 500–600 h (distance running) and up to 1,000 h per year (rowing, swimming, cycling, triathlon), performed during 400–800 training sessions (122–124). This training volume is undertaken for a minimum of 11 mo a year, with the overall goal of achieving peak performance throughout a specified time frame (usually 4–6 wk) in the competitive season. However, there is significant variability between sports, with professional cyclists frequently racing ∼100 days and riding in excess of 30,000 km during any 12-mo period (125, 126). Longitudinal data suggest that the development of a world-class endurance athlete may take up to a decade of specific training, with highly successful athletes often following a 2- or 4-yr cycle of preparation for world championships or Olympic events (110, 125). To maximize adaptation and reach one’s genetic potential, champion athletes must therefore be able to tolerate prodigious training loads. However, a high training volume alone does not guarantee sporting success.

A quarter century ago, Mujika et al. (127) studied the relationships between training variables and performance variations over the season in a group of elite swimmers. They reported that training intensity, rather than volume or frequency, was the key variable inducing a training adaptation that led to subsequent performance improvements. These workers also observed a training intensity distribution that placed emphasis on volume-overload training conducted at submaximal intensities for most of a season, with the inclusion of supramaximal high-intensity sprint workouts nearer to a competition. A decade later, this approach would be described as a “polarized training intensity distribution” by Seiler and colleagues (121) as distinct from a pyramidal training paradigm (FIGURE 4). Since then, there have been several reports that elite athletes follow both approaches to their competition preparation (128). Coetzer et al. (129) reported that elite distance runners with superior race performances trained at a higher average intensity than a group of sub-elite runners: the sub-elites spent 13% of their total weekly training volume running at speeds eliciting >80% of $\dot{\text{V}}{\text{O}}_{2\mathrm{peak}}$, whereas the elite runners spent significantly more time (36%) at this higher intensity. These observations agree with others (118, 119) who have observed that elite Kenyan distance runners complete a greater volume of training as fast-paced “tempo” runs and short-interval training compared to their non-elite counterparts. Guellich and colleagues (130) reported that elite endurance athletes from a range of sports including rowing, running, cycling, and cross-country skiing perform only a small portion of their training at competition/race-pace intensities, with the bulk of their workload comprising low-intensity, high-volume workouts and exposure to extreme HIT sessions.

It has been hypothesized that a polarized approach to training, in which 75–80% of total training volume is performed at low intensities with 10–15% performed at supramaximal intensities, may be the optimal training intensity distribution for elite athletes who compete in intense endurance-based events (131). However, this practice has recently been questioned and debated (116, 132–134). Alternative approaches to “polarized training” have been proposed, such as pyramidal or “threshold” training intensity distributions. At present, and to the best of our knowledge, there are no studies that demonstrate that adherence to a polarized training program produces superior outcomes compared with the pyramidal training programs athletes typically practice or other possible training models (116, 132–134). Indeed, polarized training per se seems totally incompatible with the principle of training specificity, a cornerstone of any training program. Although it is tempting to attribute the superior performances of elite athletes from a range of endurance sports to the adoption of a specific training regimen (i.e., polarized training, HIT), the principle of individuality dictates that the same training program will not equally benefit all those who undertake it. Furthermore, the molecular and cellular mechanisms that underpin performance enhancement after polarized and various other training interventions are not well understood. Directly linking exercise-induced molecular signaling events in skeletal muscle to defined metabolic responses and specific changes in gene and protein expression that occur after diverse training regimens may provide clues as to why certain training methods (i.e., polarized training, HIT) are such potent interventions both for promoting health outcomes and enhancing athletic performance.

#### 2.2.2. High-intensity, low-volume vs. low-intensity, high-volume training to optimize resistance training adaptation.

Analogous to endurance-based training, periodization is frequently used to promote muscle hypertrophy and strength gains in response to a program of resistance training (135). Indeed, when resistance training volume is similar, periodized training protocols induce greater gains in strength [i.e., one-repetition maximum (1RM)] than non-periodized resistance training, at least in trained individuals (135). The process of skeletal muscle fiber hypertrophy and the concomitant gains in strength/power (discussed in sects. 3.2, 4.3–4.5, and 4.8) are the result of the confluence of a net positive muscle protein balance, with the addition of satellite cells to muscle fibers a possible mechanism. Muscle hypertrophy only occurs when net positive muscle protein balance is maintained over several weeks/months and when the rate of muscle protein synthesis (MPS) exceeds that of muscle protein breakdown (136). Resistance training volume can be defined as the number of sets × repetitions, sets × repetitions × load (expressed as a percentage of 1RM), sets × repetitions × load (kg), load × sets × repetitions for each exercise, or number of sessions × repetitions × sets (137). Resistance training intensity is typically defined as a percentage of maximal strength (%1RM). Resistance training frequency represents the number of resistance training sessions performed in a specified time period (i.e., per week) and for each muscle group. The frequency of resistance training sessions is important when considering resistance exercise prescription, as the recovery time between sessions must allow for muscle adaptation (i.e., net protein synthesis). The number of training sessions provides an indication of the total resistance training work over a program’s duration, whereas including load describes the total work of a single training session. Other parameters (e.g., load, number of repetitions and sets, range of movement, time between sets, time under tension, and volitional muscle failure) provide a comprehensive description of resistance training programs (138), even though the effect of manipulating these variables on athletic performance remains unclear (139).

To maximize muscle hypertrophy, the American College of Sports Medicine (ACSM) recommends resistance training intensities corresponding to a load of ∼70–80% 1RM for 8–12 repetitions (140). Although such loading is unlikely to be undertaken by elite athletes competing in strength/power events, such advice is largely based on the observation that higher loading induces greater force development, an increased muscle electromyography activity (141), and a greater recruitment of muscle fibers. Evidence to support a dose-response relationship between external loading and maximal rates of MPS comes from the work of Kumar et al. (142), who showed that a plateau in MPS was reached at intensities approaching 90% of 1RM. However, results from other studies suggest that maximal rates of MPS can also be achieved by low-intensity, higher-volume loading. Burd et al. (143) studied 15 recreationally active males who performed four sets of unilateral leg extension exercise at different exercise loads and/or volumes: 90% of 1RM until volitional failure (90FAIL, ∼5 repetitions), 30% 1RM work-matched (WM) to 90%FAIL (30WM, 14 repetitions), or 30% 1RM performed until volitional failure (30FAIL, 24 repetitions). Low-load, high-volume resistance exercise (30FAIL) was equally effective at increasing rates of MPS as high-load, low-volume resistance exercise (90FAIL), eliciting increases in rates of myofibrillar protein synthesis similar to those induced by the 90FAIL protocol in the postexercise recovery period. Furthermore, only the 30FAIL protocol sustained higher rates of MPS 24 h after exercise. Although these data from a single bout of resistance training are intriguing, there is support for the concept that measures of acute postexercise MPS are qualitatively predictive of the chronic training-induced phenotypic changes driven by repeated resistance exercise stimuli. In a study from the same laboratory, Mitchell et al. (144) studied 18 untrained males who completed 10 wk of unilateral knee extension resistance training. Each leg of a participant was randomly assigned in counterbalanced fashion to one of three possible unilateral training conditions: one set of knee extension performed to voluntary failure at 80% of 1RM (80%-1); three sets of knee extension performed to the point of fatigue at 80% of 1RM (80%-3); or three sets performed to the point of fatigue with 30% of 1RM (30%-3). Each participant trained both legs and was therefore assigned to two of the three possible training conditions. The strength of this design is that both limbs are exposed to the same nutrient and hormonal milieu and therefore any phenotypic changes can be ascribed solely to the training stimulus. There were significant training-induced increases in muscle volume [measured by magnetic resonance imaging (MRI)], but these were not different between the training protocols. These results are in accordance with previous acute measurements of muscle protein synthetic rates and demonstrate that a lower load lifted to failure results in muscle hypertrophy similar to a heavy load lifted to failure. An important feature of this study was that the training program was underpinned by adequate nutrition (i.e., sufficient amino acid availability) to support the increases in MPS that occur after each training session. These results support earlier findings demonstrating that significant increases in muscle fiber area can be achieved after 16 wk of isometric training at 30% of maximal voluntary contraction (MVC) (145).

As is the case for most studies that have examined various endurance training protocols, most investigations of different strength/resistance training programs have been undertaken with recreational and/or moderately trained male college students. How such findings translate to elite athletes who are likely to have reached an upper limit in muscle hypertrophy and strength gains after many years of training is unclear. Elite athletes competing in events that require strength/power will also be undertaking additional forms of training to maximize muscular force output such as plyometrics, which involves rapid and repeated stretch/contractions of the muscle of the lower limb (146–148), or hypoxic/blood flow-restricted training (149–151). This makes it difficult to determine the precise contribution of any single intervention to improvements in muscle hypertrophy and strength. Inherent variability in the individual response to resistance training is also a factor to consider in any training protocol (discussed below). In summary, there is currently little consensus on how the variables related to resistance training (training load, volume, and frequency, muscle time under tension, lifting cadence, contraction mode, and interset rest interval) are most effectively periodized to maximize both MPS and improvements in strength and other functional measures (135, 139, 152, 153).

### 2.3. Exercise Interference Effects and Concurrent Training Responses

The inverse relationship between muscle fiber size and oxidative capacity highlights the principle of the specificity of training when comparing muscles of endurance and strength/power athletes (154, 155). Accordingly, simultaneously training for both endurance and strength results in a compromised adaptation compared with training for either exercise modality alone, at least in previously untrained individuals. This phenomenon was first described by Hickson (156), who reported impaired strength development in training naive males when they incorporated both strength and endurance workouts versus single-mode exercise into a short-term (10 wk) training program. Hickson (1980) coined this the “interference effect,” and since that seminal observation, a number of animal and human studies have been conducted in an effort to elucidate a molecular basis to explain this outcome (discussed in sect. 4.5.1). Of note was that training‐induced gains in aerobic capacity in that study (156) were not compromised by concurrent strength and endurance training. In fact, in contrast to the impaired strength gains observed when endurance training is undertaken simultaneously with resistance training (156), there is potential for combined strength and endurance training to amplify endurance performance (157).

The study of “concurrent training” has received less scientific enquiry than single-mode training for endurance or strength/power. Indeed, studies of concurrent training interventions pose several unique experimental challenges. The inability to match total work as well as the type of stimulus and/or exercise mode makes comparisons between the results of studies of concurrent training problematic. Differences in experimental design and dependent variable selection also limit any mechanistic insights in those studies that have determined only performance-based outcomes. Finally, the majority of studies of concurrent training to date have focused exclusively on acute molecular responses in moderately trained individuals, employing modest workloads; the training practices of elite/professional athletes undertaking concurrent training far exceed those reported in the literature for less well-trained subjects and are likely to induce complex molecular profiles (88, 158). Over the past two decades, the mechanisms that generate the adaptive response to both endurance- and strength-based exercise training have undergone intense investigation (9, 55, 85–87, 159–166). There are multiple stimuli associated with endurance- and resistance-based exercise and various signaling kinases that respond to these different perturbations, in concert with numerous downstream pathways and targets of these kinases. These events involve the increased expression and/or activity of key proteins mediated by an array of signaling events, pre- and posttranscriptional processes, regulation of translation and protein expression, and modulation of protein/enzyme activities and intracellular localization (9, 55, 85, 87, 159). These molecular processes are described in detail in sect. 4. Finally, there are complex spatial and temporal interactions between the various elements that ultimately combine to produce the integrated response to an exercise challenge that, when repeated over months and years, results in functional improvements in performance and alterations in phenotype.

Although it is convenient to classify athletic events as either “endurance-based” or “strength-/power-based,” with skeletal muscle from endurance- and strength-trained individuals representing diverse adaptive states in response to selective activation and/or repression of signaling pathways that underpin these adaptations (9, 88, 159, 160, 166), such a one‐dimensional perspective ignores the fact that the majority of athletic disciplines require a combination of both muscular endurance and strength/power for successful outcomes. As such, both endurance- and resistance-based training are frequently undertaken concomitantly as part of a periodized training program. These practices encompass several scenarios: *1*) a single training session during which an athlete performs both endurance‐ and resistance‐based exercise; *2*) two independent training sessions undertaken by the athlete on the same day, in one of which the focus is endurance adaptation (i.e., performed in the morning) and in the other strength/power adaptation (i.e., performed in the afternoon/evening); or *3*) when an athlete incorporates both types of training on different/alternate days as part of a periodized training program (88). Currently, little is known about the effects of concurrent training in elite athletes on performance progression, and it is conceivable that the degree of interference may be discipline- and training paradigm-specific (1, 167). For example, in sports where endurance as well as high peak power/forces are required (such as in 2,000 m rowing), athletes aim to maximize both muscle mass and oxidative capacity. Indeed, the peak power of Olympic rowers is positively correlated with thigh muscle volume but negatively correlated with $\dot{\text{V}}{\text{O}}_{2\text{max}}$ (168). Similarly, sprint and endurance performance are inversely related in highly trained cyclists (169).

### 2.4. Altitude and Hypoxic Training to Enhance Adaptation

Of all the practices currently used to enhance training adaptation and elite athletic performance, “altitude training” or exposure to hypoxic environments is the most widespread (55, 97). The stimulus for a new era in research of high-altitude training practices was the 1968 Olympic Games held in Mexico City at an elevation of 2,240 m above sea level. In the middle- and long-distance track events, runners who were born and trained at altitude were dominant: in the men’s 10,000 m, the first five runners resided and trained at altitude. The world record holder at the time for both the 5,000 and 10,000 m events going into the Mexico Games, Australian Ron Clarke, who was born and trained at sea level, collapsed after finishing 6th in the 10,000 m and had to be administered oxygen to recover. Since those Olympics, male and female athletes from Kenya and Ethiopia have dominated middle- and long-distance running events, with elite athletes and coaches steadfastly believing in the benefits of hypoxia-induced adaptive responses to optimize performance (97). This is despite the paucity of scientific evidence supporting an altitude-induced performance-enhancing effect (55, 170, 171). The mechanisms that underpin the adaptive response to reduced oxygen availability are discussed below.

#### 2.4.1. Into thin air: altitude training strategies to enhance endurance performance.

There are several common approaches that athletes adopt with regard to altitude training, involving several days to several weeks of exposure to some form of altitude or hypoxic challenge (172). Regardless of the different approaches used to induce hypoxic living/training conditions, the underlying physiological basis for altitude training is that the reduced barometric and partial pressure of oxygen results in lowered oxygen availability causing an increase in erythropoietin (EPO) production in the kidney that stimulates erythropoiesis and thereby leads to enhanced hemoglobin (Hb) mass. As acute exposure to hypoxia over several hours does not improve aerobic or anaerobic performance, these studies are not discussed here (173). The original altitude training strategy involved athletes spending up to 6 wk living and training at a moderate altitude (2,000–2,500 m) and returning to sea level just before a major sea-level competition (“live high, train high,” LHTH). The LHTH approach boosts EPO and Hb mass, which results in an increase in $\dot{\text{V}}{\text{O}}_{2\text{max}}$. Such adaptations usually persist for 1–2 wk upon return to sea level, with the athlete participating in several major competitions during this period. A limitation of the LHTH strategy is that training intensity is often compromised, which is in line with the linear reduction in $\dot{\text{V}}{\text{O}}_{2\text{max}}$ with increasing altitude (∼6–8% reduction per 1,000 m) (174). A second strategy involves athletes continuing to reside at sea level but training at altitude (“live low, train high,” LLTH). Adaptations resulting from LLTH are mainly confined to the trained musculature (i.e., skeletal muscle mitochondrial volume density), with little effect on EPO or Hb mass. As with the LHTH approach, the intensity of training is typically reduced with LLTH. A third protocol, and the one that is most widely used and has received widespread interest among scientists, coaches, and athletes, is the “live high, train low” (LHTL) approach, whereby athletes reside at altitude for several weeks but return to sea level to undertake the majority of their training sessions. Compared with LHTH or LLTH approaches, the LHTL approach permits athletes to maintain their absolute training loads (volume and intensity) while concomitantly gaining the physiological adaptations that accrue with exposure to chronic hypoxia. Indeed, when competitive runners completed 4 wk of supervised training as either LHTL, LHTH, or LLTH, performance of a 5 km time trial at sea level was improved only in the LHTL athletes despite similar gains in the athletes’ $\dot{\text{V}}{\text{O}}_{2\text{max}}$ in all intervention groups (175). No muscle biopsies were taken in that investigation, so it was not possible to determine whether the different altitude-training regimens resulted in changes in hypoxia-mediated signaling pathways or if there were changes in major training-induced signaling proteins. A model pioneered by the Australian Institute of Sport (AIS) requires that athletes gain exposure to altitude/hypoxia by either living in a custom-built altitude house under conditions of simulated altitude (14 h/day) or using altitude tents or intermittent hypoxic exposure with hypoxic breathing devices (176). However, even though altitude paradigms increase Hb mass (172), the purported performance gains from living at simulated moderate altitude and training at low altitude have been questioned (177, 178). Therefore, whether training in hypoxia while living in normoxia or living under hypoxic conditions while training at sea level (or low altitudes) is superior to living and training in normoxia for enhancing performance of elite athletes near sea level is unclear and warrants further investigation. There are also many challenges when assessing the effect of altitude exposure on performance in elite athletes (179). For example, the scientific gold standard design of a double-blind, placebo-controlled, crossover trial has seldom been conducted in studies of altitude training in elite athletes. A recent systematic review, albeit incorporating individuals with a wide range of athletic abilities, concluded that placebo and nocebo effects can exert a small to moderate effect on sports performance (180). Yet despite equivocal scientific evidence to support a performance-enhancing effect of altitude/hypoxic training practices, elite endurance athletes and their coaches continue to believe that some form of altitude training will confer a performance advantage when competing at sea level. Guidelines and measures to improve altitude acclimatization, tolerance, and safety have been reviewed elsewhere (181). Interestingly, preconditioning with hyperbaric oxygen has also been proposed to enhance performance, however with similar equivocal underpinnings (182).

#### 2.4.2. Resistance training under hypoxic conditions.

Acute hypoxia has been proposed to potentiate resistance training-induced hypertrophy by activating satellite cell-dependent myogenesis rather than an improvement in net protein balance. To test this hypothesis, van Doorslaer et al. (183) recruited 19 physically active male subjects who performed 4 wk of resistance training (6 sets of 10 repetitions of a 1-leg knee extension exercise at 80% 1RM 3 times/wk) in either normoxic [fraction of inspired oxygen ($\text{F}{\text{I}}_{{\text{O}}_{2}}$): 21%; *n* = 9] or hypoxic ($\text{F}{\text{I}}_{{\text{O}}_{2}}$: 13.5%, *n* = 10) conditions. At the end of the intervention, the strength gain was higher in individuals who trained under hypoxic compared with normoxic conditions, despite no changes in muscle thickness and the rate of MPS. Although these results suggest that training under hypoxic conditions may be a potent intervention to increase muscle strength, at least in the early phase of training, additional studies in well-trained athletes incorporating long-term protocols are urgently needed to determine whether hypoxic resistance training can further maximize strength gains. Other protocols with potential additive training effects due to reduced local muscle oxygen availability and exacerbated vascular shear stress that leverage hypoxic stimuli (i.e., blood flow restriction) are currently being investigated (150, 151, 184) yet hampered by the heterogeneous responses to ischemic preconditioning (185).

### 2.5. The Lowdown on Training with Reduced Muscle Glycogen Stores

A growing field of interest that has directly risen from a better understanding of the molecular bases underlying training adaptation is how nutrient availability has the capacity to modify the regulation of many contraction-induced signaling networks in skeletal muscle (sects. 3.4.2 and 4.5) (9, 186–193). The interaction between exercise training-induced responses and nutrient availability has long been recognized (194), and today it is well accepted that carbohydrate-based fuels are critical for prolonged, intense training and in the competition setting where optimal endurance performance is desired (195). However, this premise does not address the issue of whether training adaptation is driven by a surplus or lack of substrate (i.e., carbohydrate). During the past decade, there has been a growing appreciation that commencing selected training sessions with reduced muscle glycogen stores may promote training adaptation and enhance endurance performance (196–198). Acutely manipulating substrate availability (by either altering the composition and/or timing of meals before training/competition or depleting endogenous fuel stores by exercise) rapidly alters the concentration of blood-circulating substrates and hormones that modulate several receptor-mediated signaling pathways. The release of cytokines and growth factors from contracting skeletal muscle in response to the altered hormonal milieu also stimulates cell surface receptors and activates many intracellular signaling cascades (described in sect. 4). These local and systemic factors cause marked perturbations in the storage profile of skeletal muscle (and other insulin-sensitive tissues) that, in turn, exert pronounced effects on resting fuel metabolism and patterns of fuel utilization during exercise. When repeated over weeks and months, such nutrient-exercise interactions have the potential to alter numerous adaptive processes in skeletal muscle that ultimately drive the phenotype-specific variability observed between individuals (55). However, linking these molecular events to direct downstream effectors has proven elusive (199). Perhaps more to the point, training adaptation requires an increase in the steady-state levels of exercise-induced proteins, and it was not until the pioneering study of Hansen and colleagues (187) that the notion that endurance training undertaken with low muscle glycogen levels could augment adaptation gained scientific credibility. These workers tested previously untrained individuals before and after a 10-wk intervention in which both the left and right legs of the same individual were subjected to specific work-matched training protocols in which one leg was trained once daily while the contralateral limb trained twice every second day. As intended, the twice-a-day training protocol decreased muscle glycogen content after the first bout of exercise such that the second exercise session of the day was commenced with lowered (but not totally depleted) muscle glycogen content. The activity of mitochondrial enzymes along with resting muscle glycogen concentration were all increased to a greater extent when half the training sessions were executed with low glycogen availability. Exercise time to exhaustion (a proxy for performance) involving a one-legged “kicking” task was elevated markedly for both legs after training but was twice as long for the limb that trained with low compared to high glycogen. The strength of this study was the design that controlled for both systemic and local effects. However, the authors acknowledge that the controlled laboratory setting, coupled with the training status of their subjects, may not permit the results to be extrapolated to competitive athletes. Several studies subsequently verified the finding that, in well-trained athletes, chronic (3–10 wk) training programs in which selected workouts were deliberately commenced with low muscle glycogen concentration increased the expression of genes and the abundance of proteins involved in carbohydrate and/or lipid metabolism while promoting mitochondrial biogenesis to a greater extent than when all workouts are undertaken with normal or elevated glycogen stores (molecular mechanisms underlying these observations are discussed in sect. 4.5.2) (199–201). Surprisingly, such adaptations accrued notwithstanding a reduction of 7–8% in the athletes’ self-selected training intensity (200, 201). Yet despite augmented adaptations at the muscle level, studies that have examined the “train low” glycogen model in well-trained athletes have often (201–204), but not always (197, 198), failed to show a performance benefit (for review, see Ref. 205). Such a disconnect between changes in selected molecular mechanistic variables (e.g., increases in the phosphorylation status of signaling molecules and/or increases in the expression of genes and proteins involved in mitochondrial biogenesis) and whole body functional outcomes (changes in training capacity or athletic performance) is hard to reconcile. However, it may well be that elite athletes with a prolonged history of training have already maximized many of the cellular pathways involved in energy provision and that proteins in these and other contraction-induced pathways that are upregulated with the train low glycogen protocol are not rate limiting for performance.

There is a scarcity of studies that have examined the effects of commencing resistance training with low muscle glycogen stores. Nevertheless, some evidence exists suggesting that reduced glycogen availability may upregulate cellular pathways regulating mitochondrial biogenesis after a single bout of exercise (206), even though engaging in resistance training with low muscle glycogen does not affect rates of MPS (207). These results imply that commencing a bout of strenuous resistance exercise with low muscle glycogen availability attenuates neither anabolic signaling nor rates of myofibrillar protein synthesis. In summary, despite no clear evidence of a performance-enhancing effect from the results of several well-controlled laboratory-based studies that have tested various train low (glycogen) strategies, many athletes who compete in endurance-based events continue to incorporate such practices into their training programs. In contrast, there appears no reason for athletes undertaking resistance training regimens to adopt low-glycogen workouts into their daily schedules. A challenge for future investigations is to directly link some of the acute exercise-induced molecular signaling events in skeletal muscle that take place in response to the greater metabolic loading imposed by various training interventions (i.e., altitude and low glycogen) to defined performance-related outcomes that occur after elite athletes undertake such practices.

### 2.6. A Time to Train, a Time to Compete?

Since the awarding of the Nobel Prize in Physiology or Medicine in 2017 for the discovery that the molecular clock is the primary mechanism underlying circadian rhythms, there has been a dramatic increase in the number of scientific publications regarding circadian biology and its impact on various aspects of human behavior, including sporting performance. Circadian rhythms are ∼24-h (*circa diem*) oscillations in biological and metabolic pathways. The circadian clock is cell autonomous and present in most human tissues and organs and is organized in a hierarchical manner, with the hypothalamic suprachiasmatic nucleus (SCN) functioning as the “master clock” with “fine-tuning” by clocks in peripheral tissues (208–211). Although light is the dominant zeitgeber (time giver) for the SCN oscillator, which in turn orchestrates rhythms in the peripheral organs/tissues at appropriate phases, both the timing of exercise (212–216) along with the scheduling of meals (217–220) can impact circadian behavior (molecular underpinnings are discussed in sect. 4.9).

Differences in the time of day for peak performance for strength and anaerobic power as well as oxidative capacity and endurance performance have been reported in many, albeit not all, human studies (221–224). However, there are large interindividual differences in circadian rhythms, and the time of day for peak performance is affected by many additional factors including time since awakening, timing of precompetition meals, sleep quality, body temperature, hormone levels, psychological habituation, motivation, and prior muscle fatigue (225–227). Accordingly, the effect of the time of day of training on performance needs to be placed in the context of an athlete’s chronotype. An individual’s predisposition toward a preference for either morning or evening can be classified into early chronotypes (ECTs), late chronotypes (LCTs), or those in between (intermediate chronotypes, ICTs) (228). ECTs, sometimes referred to as “larks,” have significantly earlier sleep-wake cycles compared with LCTs (or “night owls”), who function better later in the day. These differences are not only observed in sleep-wake cycles but also multiple physiological (229), behavioral (228), and genetic (230) oscillations that occur every 24-h period. The implications for competition performance are not entirely clear. Diurnal performance profiles have been studied between ECTs and LCTs to determine whether there is significant variation when individual aspects of circadian timing are considered. These investigations show clear differences in performance profiles between ECTs and LCTs, with LCTs exhibiting greater variation in diurnal performance profiles, particularly in the morning (231). Interestingly, performance peaks can be shifted by different measures such as active and passive warm-up, caffeine, or training-competition time-of-day synchronization (225). Moreover, individual shifts in chronotypes or time-of-day performance are observed (i.e., in older athletes with a higher prevalence of “morningness” in training scheduling and work rates) (221).

The impact of exercise training at different times of the day has been well studied in animal models and healthy moderately trained humans, with the primary outcome typically being a measure of exercise capacity, often a laboratory-based task designed to mimic performance, or a metabolic surrogate (232–234). However, studies investigating the timing of exercise training in elite athletes and the subsequent effect on performance outcomes are scarce. Once again, we are left to generalize from interventions in healthy, almost exclusively male, non-elite subjects until such gaps in the literature are filled. There are several reports of greater increases in muscle mass and strength after training late in the afternoon versus early morning (221, 233–235), which is in line with the generally higher peak forces attained in the afternoon/early evening (236). Consistent with the enhanced reliance on fatty acid oxidation in a fasted state in the early morning in humans (237), there is a more robust metabolic impact of exercise in the fasted state (at the beginning of active phase in rodents) than in the fed state (at the beginning of the rest phase) (214). Regardless, the results are likely to have limited translational value for elite athletes who typically undertake several workouts within any 24-h period supported by round-the-clock eating patterns necessary to meet the demands of training (81).

While elite athletes are informed of the venues, dates, and times of major international competitions several years in advance, the nations selected to host the Olympic Games and World Championships often adjust competition times to accommodate and coincide with prime-time viewing hours for North American television audiences. At the recent Tokyo Summer Olympics, the entire swimming program was “flip-flopped” such that qualifying heats and semifinals (normally held in the morning) were scheduled for the evening and all finals were swum in the morning. As circadian oscillations affect physiological, psychological, and molecular mechanisms resulting in varying physical performance capacities over the day, both the timing and relative size of these effects are important for optimizing sport performance at the elite level. To determine the extent to which elite athletes are affected by circadian fluctuations in physical performance, Lok et al. (238) assessed data from four Olympic Games (Athens 2004, Beijing 2008, London 2012, and Rio de Janeiro 2016). The authors analyzed swimming performances, as these races are less likely to be influenced by confounding environmental effects (i.e., temperature, humidity, wind speed) and have little reliance on equipment that could induce variation within and between athletes. Additionally, the water temperature in the pool is required to be within a narrow range across Olympic venues, providing a “clean” signal of daily variation in physical performance (238). Their analysis revealed that performance in Olympic swimmers was significantly affected by the time of day, with best performance occurring in the late afternoon/early evening. The amplitude of the effects of time of day was 0.37%, and in 40% of the finals this effect was larger than the time difference between gold or silver medal finishing times. Furthermore, time-of-day effects exceeded the time difference between the silver and bronze medals in 64% of the finals and the time difference between bronze and fourth place in 61% of the finals (238). These data indicate that despite athletes incorporating both morning and evening workouts, endogenous circadian clocks still exert a time-of-day effect on elite swimming performance. Whether the application of circadian or time-of-day principles can optimize training and improve performance of these elite athletes remains to be determined.

### 2.7. Training Strategies and Paradigms: Good, Bad, or Indifferent?

The identification of training strategies that consistently enhance performance remains challenging because of the multiple interdependent factors contributing to athletic success. Consensus emerging from observational studies reflects the current practices in long (239), middle distance (146), or sprint (240) disciplines, but these are likely to be modified with technological advances and insights from coaches and “science-driven” initiatives, such as the “sub-2 hour” marathon project. However, training intervention studies are often limited to low participant numbers, with a reliance on a restricted pool of young, male, college-educated, recreational or untrained cohorts. Extrapolation to other demographics, including women, underrepresented ethnicities, or elite athletes is problematic. For example, significant sex differences exist in the response to both acute exercise and chronic training adaptation (241–243), and the effects of reproductive status, endogenous and exogenous hormones, and the menstrual cycle are underappreciated not only in research studies but also in training program design and application (244). Importantly, sex differences extend to many other training-related factors, including muscle mass and strength, injuries, and even training participation rates (245). On occasion, understudied approaches can lead to detrimental outcomes, as observed for the transient hype surrounding the so-called benefits of cold-water immersion, whole body cryotherapy, and other passive recovery strategies that in certain contexts can adversely affect recovery or performance outcomes (246, 247). New training strategies are often based on observations of athletic performance in extreme conditions, such as the high altitude of the 1968 Olympic Games held in Mexico City or the high temperatures that were expected for the Tokyo 2020 Olympic Games (held in 2021 because of the coronavirus pandemic). The former contributed to the widespread study and adoption of high-altitude training, whereas the latter was a primer to explore heat training as a modality to improve performance not only in hot environments but also in mild or cold temperatures. The potential mechanisms for enhanced performance in thermoneutral environments after heat exposure, improved thermotolerance, enhanced heat dissipation, expanded plasma volume, elevated hemoglobin mass, and other adaptations triggered by heat exposure, are discussed in sect. 4.6.1 (248, 249). Safety is an obvious concern for such an intervention, necessitating close monitoring of core body temperature and cardiac-related parameters (250). In most cases, many of the reservations about specific training strategies stem from an inadequate understanding of the systemic, organ/tissue, cellular, and molecular events that occur in response to an acute exercise bout and how such information translates into long-term adaptation. In sects. 3 and 4, we summarize the current knowledge of the physiological, cellular, and molecular underpinnings of muscle plasticity triggered by both endurance- and resistance-based exercise.

## 3. PHYSIOLOGICAL AND CELLULAR ADAPTATION TO EXERCISE TRAINING: FUNCTIONAL RESPONSE

The cellular, tissue/organ, and whole body adaptations that occur when exercise bouts are repeated over months and years drive the phenotypic changes observed in highly trained athletes. Such adaptations include alterations in energy flux and metabolism, fiber type transformations, enhanced mitochondrial and capillary density, and muscle hypertrophy, highlighting the enormous plasticity of skeletal muscle (TABLE 1). Although endurance training predominantly induces numerous metabolic adaptations that match muscle energy supply to demand and improve economy of motion, athletes engaging in sports that require high peak forces demonstrate marked changes in muscle morphology and cross-sectional area (CSA). When training adaptation is maximized in the face of favorable genetic predisposition, extraordinary performances can be achieved, such as the first sub-2-h marathon by Eliud Kipchoge in 2019 (unofficial record of 1:59:40.2), the 100 m time of 9.58 s in 2009 by Usain Bolt, or the current world record holder Hafþór Júlíus Björnsson, who was able to deadlift 501 kg. These performances highlight the remarkable potential of skeletal muscle to generate huge amounts of energy (adenosine triphosphate, ATP) for a sustained period in order to run at a speed of 21.2 km/h for ∼2 h, to rapidly contract muscles to be able to run at speeds exceeding 37 km/h for several seconds, or to lift 500 kg. In this section, we discuss the adaptations observed in elite athletes that allow such extraordinary efforts.

**Table 1.** Reference values of sedentary individuals and elite endurance athletes

	Sedentary		Elite Athlete		References
	Men	Women*	Men	Women*	
$\dot{\text{V}}{\text{O}}_{2\text{max}}$, mL/min/kg	<45	<40	∼70–85	∼60–75	(122, 251–265)
$\dot{\text{V}}{\text{E}}_{\text{max}}$, L/min	120–140	∼95	165–185	∼125	(261, 263, 266–273)
Stroke volume, mL/beat					(251, 253, 265)
At rest	∼65	∼55	∼110	∼70	
Maximum	∼100	∼70	150–200	∼125	
Cardiac output, L/min					(251, 253, 265, 274–276)
At rest	∼5–6	∼3.5–4.5	∼5–6	∼3.5–4.5	
Maximum	∼20	∼15	∼30–40	∼25	
Lactate threshold, % $\dot{\text{V}}{\text{O}}_{2\text{max}}$	∼60	∼60	75–85	75–85	(80, 122, 264, 277)
Fiber type, % type I	40–50	40–50	>60	>60	(169, 254, 256, 278–284)
Capillary-to-fiber ratio	1.5–2	Similar or slightly lower	2.5–3	insufficient data	(169, 254, 255, 278, 285–288)
Mitochondrial volume density, %	4–5	Similar or slightly lower	7.5–9	insufficient data	(254, 255, 285, 286, 289, 290)

Sedentary men and women are between 20 and 30 yr of age. All values of muscle tissue originate from vastus lateralis biopsies. $\dot{\text{V}}{\text{E}}_{\text{max}}$, maximal exercise-induced pulmonary ventilation; $\dot{\text{V}}{\text{O}}_{2\text{max}}$, maximal oxygen uptake. *Less data are available for female athletes and sedentary control subjects.

### 3.1. Oxygen Transport and Maximal Oxygen Uptake

**FIGURE 6.**
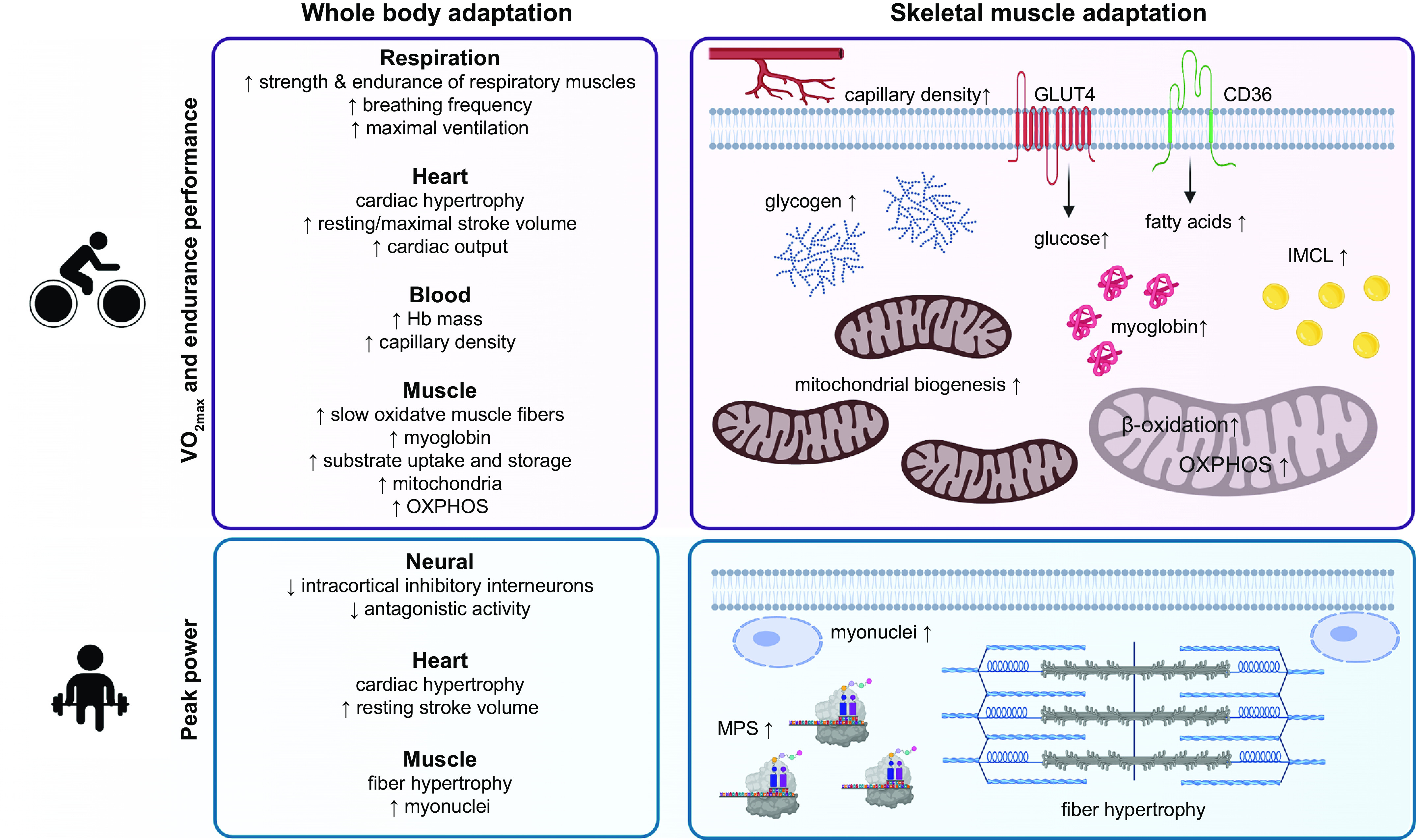
Whole body adaptations that contribute specifically to higher peak power or endurance performance. Although mainly neural and muscular adaptations improve peak power, for endurance performance various organs and tissues show major changes. To maximize $\dot{\text{V}}{\text{O}}_{2\text{max}}$ and thereby endurance performance, changes in respiratory and cardiovascular function as well as adaptations in skeletal muscle are required. In skeletal muscle, the high mitochondrial density, elevated substrate (i.e., fatty acids and glucose) uptake and storage, myoglobin content, and increased vascularization all contribute to the elevated performance of endurance athletes. Strength training-induced adaptations include increased muscle protein synthesis (MPS) resulting in fiber hypertrophy and optimally myonuclear accretion. CD36, platelet glycoprotein 4; GLUT4, glucose transporter type 4; IMCL, intramyocellular lipids. See glossary for other abbreviations. Sport icon vectors were created by ibrandify/Freepik. Image created with BioRender.com, with permission.

Exercise of prolonged duration and/or high intensity presents a major challenge to whole body homeostasis and is associated with extensive perturbations in numerous cells, tissues, and organs that are caused by, or are a response to, the increased metabolic activity of contracting skeletal muscles. To meet this challenge, multiple integrated responses are rapidly engaged to blunt the acute homeostatic threats generated by exercise-induced increases in muscle substrate turnover and oxygen demand (9). When repeated over time (i.e., exercise training), there is a coordinated process of adaptation that can be broadly categorized as either “central” (nervous, respiratory, and cardiovascular systems) or “peripheral” (skeletal muscle). However, such a simplistic classification does not completely characterize the interdependent nature of these processes. For example, pulmonary oxygen diffusion, Hb levels, cardiac output, vascularization of the muscle, as well as oxygen extraction and utilization by the muscle during oxidative phosphorylation (OXPHOS) all contribute to the endurance training-induced increase in maximal oxygen uptake ($\dot{\text{V}}{\text{O}}_{2\max}$) (FIGURE 6) (291). $\dot{\text{V}}{\text{O}}_{2\max}$ therefore is a measure of the combined capacities of the central nervous system to recruit motor units, the pulmonary and cardiovascular systems to deliver oxygen to contracting muscles (including erythrocyte number and heme loading), along with the ability of those muscles to extract and use oxygen via oxidative metabolic pathways (9). At rest, whole body oxygen consumption is ∼3.5 mL/kg/min, with ∼25% of this being taken up by skeletal muscle (251). In untrained humans, $\dot{\text{V}}{\text{O}}_{2\max}$ is ∼10- to 15-fold greater than resting values (i.e., 30–50 mL/kg/min). In elite endurance-trained athletes, $\dot{\text{V}}{\text{O}}_{2\max}$ can be twofold higher compared with non-athletes, with the highest $\dot{\text{V}}{\text{O}}_{2\max}$ values being 96 and 80 mL/kg/min for male and female endurance athletes, respectively (252, 292, 293). The lower $\dot{\text{V}}{\text{O}}_{2\max}$ values for women are reflected in the female world records for endurance events, which are typically 10–12% slower (294). Such differences are mainly due to the lower absolute and relative muscle mass in women and the lower Hb levels (294). Differences in $\dot{\text{V}}{\text{O}}_{2\max}$ are also observed based on the demands of the sport and the associated mode of training. Elite male cross-country skiers and rowers engage a larger proportion of their muscle mass (upper and lower body) than cyclists or distance runners during training and exhibit higher $\dot{\text{V}}{\text{O}}_{2\max}$ values. The training-induced increases in $\dot{\text{V}}{\text{O}}_{2\max}$ are largely confined to athletes competing in endurance-based events: power/strength-trained individuals often have $\dot{\text{V}}{\text{O}}_{2\max}$ values similar to non-athletes (266). Therefore, parameters affecting $\dot{\text{V}}{\text{O}}_{2\max}$ are differentially impacted by the training prescription.

In most contexts, the limits of maximal oxygen consumption are multifactorial and not attributable to any single parameter (295). However, in general, exercise performance is not limited by the respiratory system, as its capacity exceeds demand during maximal exercise (296). However, functional training-induced changes still occur in athletes, including a greater strength and fatigue resistance of the respiratory muscles resulting in higher maximal voluntary ventilation (MVV) and forced vital capacity (FVC) (266, 296–298). Maximal tidal volume is similar in athletes and non-athletes, with the higher maximal exercise-induced ventilation brought about by an elevated breathing frequency (266). Hb levels are similar in highly trained endurance athletes and untrained individuals (266) and are only elevated by altitude training interventions, discussed in sect. 2.4. (299). However, because of a training-induced increase in blood volume, total Hb mass may be higher (300, 301). In addition to the increased strength of respiratory muscles, endurance athletes have cardiac hypertrophy that is characterized by greater left ventricular mass and relative wall thickness resulting in a higher maximal and resting stroke volume and a corresponding lower resting heart rate (253, 266). Although maximal stroke volume plateaus at ∼40–50% of $\dot{\text{V}}{\text{O}}_{2\max}$ in untrained individuals, stroke volume increases until volitional exhaustion, contributing to the augmented $\dot{\text{V}}{\text{O}}_{2\max}$ in elite endurance athletes (253). In these individuals, maximum stroke volumes of 200 mL/beat have been reported, indicating that a cardiac output of up to ∼35–40 L/min could be reached, a figure almost double that observed in non-athletes (251, 274). Whereas left ventricular mass and resting stroke volume are similar in power and endurance athletes, $\dot{\text{V}}{\text{O}}_{2\max}$ is not elevated in power athletes, likely because of the increased maximal stroke volume and oxygen pulse per stroke volume after endurance but not resistance training (266). Finally, vascularization of skeletal muscle also contributes to $\dot{\text{V}}{\text{O}}_{2\max}$ (154). Capillary density in all fiber types is ∼50% higher in elite endurance-trained compared with nontrained individuals (254, 278), and athletes with superior muscle vascularization are even more fatigue resistant compared to athletes with similar $\dot{\text{V}}{\text{O}}_{2\max}$ values (80). The endurance training-induced vascularization occurs rapidly, with 6 wk of intense training being sufficient to elevate capillary density and capillary-to-fiber ratio (molecular mechanisms driving this adaptation are described in sect. 4.5.3) (255, 302). In contrast, capillary density in power/strength-trained athletes does not increase with training and may be lower than that in untrained individuals because of the fiber hypertrophy (278, 303). Collectively, central adaptations occur at multiple levels and play an important role in the high $\dot{\text{V}}{\text{O}}_{2\max}$ in elite athletes.

### 3.2. Neuromuscular Control and Force Generation

In contrast to the high $\dot{\text{V}}{\text{O}}_{2\max}$ required for optimal endurance performance, many sports require high power generation, including sprint events (running, swimming, cycling, and rowing) and weightlifting, powerlifting, and throwing events. Maximal performance in elite strength/power athletes is ∼15–20% lower in females than in males (304), because of differences in lean body mass between men and women (168, 305). In line with the lower lean mass, fat mass of female athletes is about twofold higher compared to men with similar body mass (304). With increasing age, differences in maximal performance in terms of world records in female and male masters athletes become greater, and records are ∼30–50% lower in women, mirroring the sex differences observed in untrained and recreationally trained individuals (304).

Voluntary muscle contraction is a complex task requiring a highly coordinated interplay on multiple levels including supraspinal structures, spinal cells, afferent feedback and efferent input, and the motor unit (306). The motor unit consists of the soma, dendrites, and axon of the motor neuron as well as the innervated muscle fibers. The force-generating capacity of the muscle is determined by the number of activated motor units, the discharge rate (also described as firing frequency or rate coding) of the motor neuron, and the size and contractile properties of the activated muscle fibers. To initiate muscle contractions, the central nervous system sends commands to the motor neurons located in the ventral horn of the spinal cord (307). Motor neurons integrate the signal from a number of different regions and nuclei in the cortex and brain stem, interneuron circuitries, as well as the peripheral sensory input from afferent fibers located in the muscle spindles and Golgi tendon organ into an action potential. The action potential propagates along the axon of the motor neuron to the innervated muscle fibers and, through acetylcholine receptor activation and excitation-contraction coupling (ECC), results in mechanical output by the muscle. In comparison to central nervous system synapses, the neuromuscular junction (NMJ) has a very high safety factor, and a nerve action potential results in an end-plate potential (EPP) of a local depolarization of ∼30–40 mV, which is higher than required to elicit an action potential in the muscle. Several morphological and functional parameters also contribute to this high safety factor (308–310): first, an extraordinarily large size of the synapse, ∼100- to 200-fold bigger compared with central nervous synapses in the mouse, and thus ample interaction surface; second, a high density of voltage-gated Ca^2+^ channels in the active zones, coupled to a high concentration of acetylcholine in a synaptic vesicle; third, the number and density of acetylcholine receptors, and the concentrated localization at the crest of postsynaptic folds, adjacent to voltage-gated Na^+^ channels (Nav1.4) in the corresponding troughs; and fourth, the strong enzymatic activity of acetylcholinesterase in the synaptic cleft for rapid removal of acetylcholine and thereby prevention of repeated activation of individual acetylcholine receptor channels in response to a single action potential in the motor neuron. Together, these properties lead to an “all-or-none” activation (as first described by Henry Pickering Bowditch in 1871 for cardiac muscle, later expanded to skeletal muscle), meaning that once the stimulus threshold for an action potential in the motor neuron is reached (based on the integration of different incoming signals), an action potential and contraction in the muscle fiber is inevitably triggered. The frequency of activation (rate coding) of the muscle fiber is important for the generation of force (311). In a single muscle twitch, Ca^2+^ reenters the sarcoplasmic reticulum and fiber relaxation becomes complete. A more frequent stimulation results in wave summation, and thus greater force, ultimately maximizing in a tetanus, in which twitches overlap and no relaxation can occur. In rodents, exercise-induced NMJ remodeling has been observed affecting morphology and function of this synapse (312, 313). For example, endurance training boosts the amount of neurotransmitter released per action potential, concomitantly with an upregulation of acetylcholinesterase (313, 314). Furthermore, an enlargement in the interaction surface is achieved by a modulation of the number and length of nerve terminal branches, coupled to an elevation in the total area occupied by presynaptic neurotransmitter vesicles and postsynaptic acetylcholine receptors (314). Similar adaptations are observed in genetic mouse models of endurance training, with corresponding changes in neuromuscular transmission properties (315). Notably, however, size, complexity, and fragmentation of murine and human NMJs can differ substantially (316). Data describing training-induced NMJ plasticity can therefore only be extrapolated to humans with caution, in particular since corresponding interrogations in humans are lacking.

**FIGURE 7.**
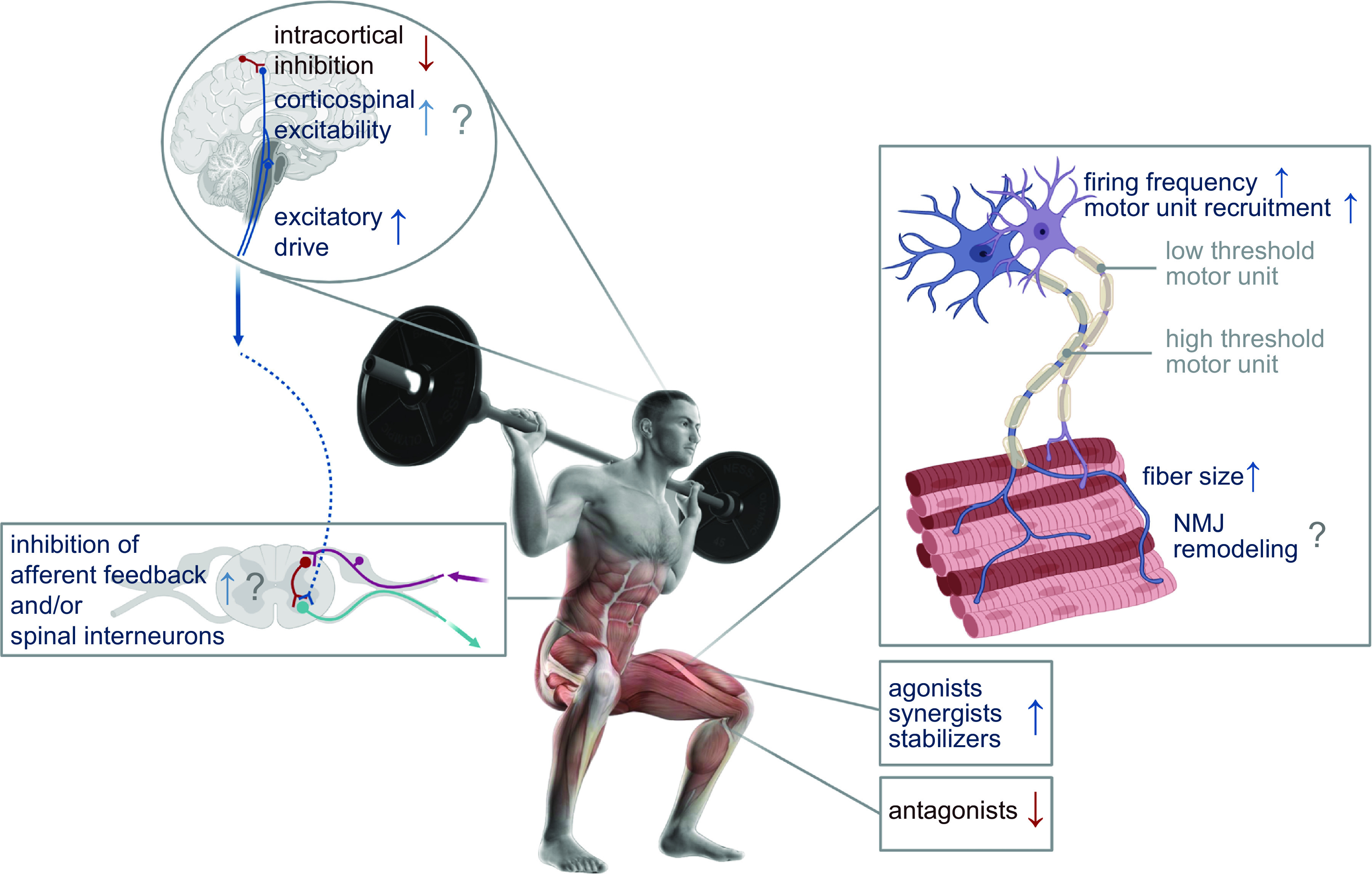
Neuromuscular adaptation to training. The number of activated motor units, their firing frequency, as well as size and contractile properties of the muscle fibers determine total force-generating capacity. In trained individuals, neural adaptations include an increased excitatory drive that can lead to an elevated firing frequency and higher number of activated motor units. In addition, the enhanced activation of agonists, synergists, and stabilizers together with the reduced coactivation of antagonists contribute to the increased force production after training. For many of these adaptations only data in rodent models exist, and/or controversial findings in humans have been reported. NMJ, neuromuscular junction. Illustration of person was created by kjpargeter/Freepik. Image created with BioRender.com, with permission.

Whereas hand muscles have fewer motor units than large limb muscles, the number of motor units of different limb muscles varies and is not always related to muscle size (317). In contrast, the average innervation number (number of muscle fibers innervated by a single motor neuron, also called motor unit size) strongly correlates with muscle size (317). Innervation numbers, even within one muscle, can range from tens to thousands, and thereby enable diverse actions such as fine-tuning of the movement or high force generation, respectively (317, 318). Whereas slow type I muscle fibers (discussed in sect. 3.3) are mostly part of motor units with a low innervation number, motor units with a high innervation number often connect to fast type II fibers (317). The motor neurons of these different motor units exhibit considerable morphological and functional differences (FIGURE 7). For example, motor neurons innervating type II muscle fibers in general have larger somas and more dendrites as well as a larger axonal diameter sizes, the latter enabling faster conductance velocity (306, 319). The physical dimensions of the motor neuron somas contribute to the determination of the recruitment threshold (320). Thus, the larger surface area and high number of ion channels in fast motor neurons result in a lower input resistance compared with the small surface area with fewer ion channels of slow motor neurons (320). According to Ohm’s law (*V* = *I* × *R*), the same synaptic input thus induces greater changes in the membrane potential of small motor neurons (with a higher resistance) compared with large motor neurons (with a lower resistance). Consequently, small motor neurons reach the firing threshold with less synaptic input compared with their larger counterparts. This orderly recruitment was shown in animal preparations by Henneman (320), and according to Henneman’s size principle small motor neurons innervating slow type I muscle fibers are recruited first, subsequently followed by larger motor neurons innervating type IIA and finally IIX fibers (306). This leads to a gradual and smooth increase in muscle force (graduation of contraction) and a predominant activation of slow and fatigue-resistant small motor units until these are overwhelmed by strong, powerful movements necessitating the recruitment of fast-twitch, high peak force-generating fibers (306). Although the size principle holds true for specific laboratory conditions, physiological systems that include excitatory and inhibitory inputs are much more complex, and it is debatable whether motor units are always recruited in a graded manner (319, 321). Recent evidence suggests that during slow, ramping movement orderly recruitment occurs, whereas high-frequency, sinusoid types of contractile activity are not following the size principle (322). Moreover, motor unit recruitment is affected by the length of the muscle (322). This implies that, during ballistic training, recruitment is observed in a more selective manner corresponding to the functional movement according to the neuromechanical matching principle (319, 321). However, even during slow ramping movement, as might be encountered in weightlifting, fast motor units can be recruited with submaximal load (138, 323, 324). In fact, a number of studies have demonstrated that even low-load exercises such as 30% 1RM result in recruitment of type I and II fiber if completed until failure, inducing a hypertrophic response similar to high-load training (138, 323–325). This finding might be explained by the observation that with increasing muscle fatigue more motor units are being recruited to meet the same force output, even when lower loads are used (138, 323, 324). Therefore, the recruitment of slow and fast motor units is dependent not only on the applied load and generated force but also on the fatigue state, contraction velocity, and length, as well as potentially other parameters that are extracted from skeletal muscle in different exercise protocols and paradigms. Some of these factors, such as velocity and length, might induce selective rather than the orderly recruitment of motor units postulated in Henneman’s size principle. In summary, motor unit engagement, recruitment, and fatigue are still poorly understood. Moreover, the plasticity of this structure in training and the specific adaptations in elite athletes are largely unknown. Of note, based on the transcriptional profile, even more distinct motor neuron pools exist than the classically defined three types, fast fatigable, fast fatigue-resistant, and slow (326). The functional relevance of this more fine-grained specification remains unknown.

#### 3.2.1. Neural adaptations.

Increased force generation can be achieved by neural adaptation, muscle hypertrophy, and/or intrinsic changes in contractile properties (strength/power per unit of muscle mass). MVC is substantially elevated in resistance training before any increase in muscle CSA, suggesting that neural adaptations mainly contribute to the strength gains in the initial phase, followed by structural changes within the muscle. The activity of intracortical inhibitory interneurons is lower in trained individuals, whereas data on corticospinal excitability are equivocal and likely unaffected by training (327–329). The reduction in intracortical inhibition in response to strength training induces a higher excitatory drive, which may contribute to elevated strength in these individuals. With augmenting force generation, the number of active motor units as well as their activity in terms of discharge rate increase (318, 330). Accordingly, the higher descending neural input in strength-trained individuals could explain the elevated discharge rate and adequate activation of motor units, culminating in the observed enhanced voluntary activation and maximal force (318, 331, 332). In addition, the elevated neural drive might be important for explosive power, as a fast recruitment and a high discharge rate of motor units are important for the rate of force development (330). The lower recruitment threshold observed after strength training suggests that, in addition to the changes in neural drive, intrinsic properties of motor neurons may be altered by training (331). The timing of the action potentials discharged by concurrently active motor units of strength athletes appears to exhibit greater synchronization than that in untrained individuals, even though it is debatable whether these adaptations in intramuscular coordination contribute to strength gains (333). Intermuscular coordination is also improved at several levels. Besides the recruitment of agonists, stabilizers/fixators, and neutralizers/synergists, reducing the coactivation of antagonists significantly contributes to the maximal voluntary activation and force generation in highly trained athletes (332–334). Whether these training adaptations evoked by intermuscular coordination are primarily mediated by disinhibition of supraspinal signals, altered activity of Renshaw cells and other spinal interneurons, and/or adaptations in afferent feedback, such as decreased stretch inhibition by the proprioceptive system, is unclear. Nevertheless, the importance of early neural and neuromuscular adaptations and the concomitant optimization of intra- and intermuscular coordination before structural changes of the muscle in resistance training adaptation is irrefutable (FIGURE 7) (329, 335, 336). The corresponding neural changes in endurance training are less well characterized but could contribute to improved running economy, decreased fatigability, and other interrelated parameters (337–339).

#### 3.2.2. Muscle hypertrophy.

Powerlifting or hammer throw athletes rely on the generation of instantaneous maximal peak forces, whereas sports that involve short-duration sprints require high contractile velocity of muscles over several seconds. Nonetheless, an increase in muscle mass to generate high contractile forces is a common goal for these athletes. Indeed, the muscle volume of the lower limbs of both sprinters and other strength-trained athletes is higher compared with endurance athletes and untrained individuals (340, 341). The training-induced gain in muscle CSA is not evenly distributed along the length of the muscle fiber but occurs predominantly in the midbelly region of the muscle, which explains why the percent increase in CSA can exceed that of muscle volume (335, 342). In humans most muscles are pennate, in which the increase in physiological CSA of the muscle, reflecting the radial growth of the myofiber, can diverge from the increase in anatomical CSA. To optimize the limited space of the aponeurosis, a larger CSA is usually accompanied by a steeper pennation angle, which is greater in highly trained strength athletes compared with untrained individuals (343) and strongly correlates with muscle thickness (344). In contrast, in elite sprint athletes (100 m sprinters and sprint cyclists), muscle thickness is increased without changes in the pennate angle (345, 346). As these observations in elite athletes are all cross sectional, it remains to be determined whether the architectural differences contributing to peak performance are the result of long-term training adaptation or genetic predisposition. Despite these findings, the contribution of the pennation angle and other architectural properties to muscle functionality and power generation remains contentious (347, 348).

Of note, hypertrophy of myofibrillar and sarcoplasmic compartments has been described, and the relative impact on muscle mass and strength gains remains equivocal, similar to the importance of “conventional” hypertrophy with a proportional increase in myofibrillar protein content and tissue growth compared with “unconventional” hypertrophy, for example achieved by myofibrillar packing preceding an increase in fiber size (349). Nevertheless, in most cases resistance training induces radial growth of the muscle, resulting in a higher CSA (mechanisms underlying this response are described in sects. 4.1 and 4.3–4.5) (342, 350). The expansion of myofibrillar protein resulting in fiber hypertrophy might contribute the most to enhanced force-generating capacity of a muscle fiber. Within the muscle fiber, ∼80% of the volume consists of myofibrils that are composed of sarcomeres, the contractile units of the myofibril (351). The thin actin and thick myosin filaments constitute the two major active components of the sarcomeres responsible for muscle contraction. Upon Ca^2+^ binding to troponin C (TnC), tropomyosin conformation changes to expose the myosin-binding site on the actin filament. The thick myosin filaments, the force-generating elements of the sarcomere, bind to actin and induce the sliding of actin filaments along the myosin, resulting in muscle shortening. The addition of sarcomeres in parallel rather than in series causes an increase in fiber diameter (342). In line with the high potential of type IIA fibers to increase CSA and force generation (352), hypertrophy predominantly occurs in type IIA fibers in elite strength-trained athletes (303, 350). Besides radial growth, inclusion of additional sarcomeres in series leading to increased fascicle strength has been reported (342). Limited data are available regarding the longitudinal growth of the muscle in response to resistance-based training, although there is evidence that fascicle length can increase (335, 343). For example, a longer fascicle length is observed in elite sprinters (345, 346), which is positively correlated with performance times (343, 353, 354). The longer fascicles could contribute to a greater shortening velocity of a pennate muscle and thereby enhance sprint performance (353). However, the total number of sarcomeres in series in a muscle fiber and the effects of training are difficult to determine in humans, and the few studies in rodents revealed mixed results (342). Moreover, recent evidence indicating a mesh-type network of branching sarcomeric structures instead of individual sarcomeres existing in separated tubes further complicates the interpretation of changes in sarcomere numbers in series and in parallel (355).

Despite the fundamental contribution of radial muscle growth to maximal power output, the relationship between force generation and muscle CSA is not linear, emphasizing the contribution of other factors (356). One possibility could be that muscle quality rather than size is enhanced, resulting in a higher specific force (force per CSA) (352). For example, the specific force of type I fibers has been shown to increase in response to resistance exercise (352). Additionally, changes in fiber type distribution could enhance specific force capacity, since both the force-generating capacity per myosin head as well as the fraction of attached myosin heads are higher for fast myosin heavy chain isoforms (357). Despite these changes, the increase in overall muscle strength is superior compared with the integration of single-fiber strength gains, indicating that optimal strength gains occur when both neural as well as muscular adaptations take place (352).

The increase in myofibrillar proteins is often, but not always, accompanied by elevation of the number of myonuclei, potentially to optimize the hypertrophic response. The syncytial nature of muscle cells has been hypothesized to be due to the limited capacity of (myo)nuclei to provide transcripts for a certain volume of the cytoplasm, defined as the myonuclear domain (358). According to this hypothesis, the upper limit of the myonuclear domain is determined by the maximal transcriptional capacity of myonuclei. Once this ceiling is reached, the number of myonuclei was thought to be increased by the fusion of satellite cells to muscle fibers to maintain a relatively constant DNA-to-cytoplasm ratio (reviewed in Ref. 358), a concept that has subsequently been refined and corrected (359). Moreover, in humans hypertrophy has also been reported in the absence of myonuclear accretion (360, 361). Furthermore, although the addition of myonuclei is greater when hypertrophy exceeds 22% of size gain, it also occurs when hypertrophy is <10% (362). According to a recent meta-analysis, a definitive “hypertrophy threshold” required for myonuclear accretion remains tenuous, and thus the threshold hypothesis appears problematic (362). Nevertheless, cross-sectional data from athletes often show hypertrophy to be positively correlated with myonuclear number (363). For example, in elite powerlifters, both the size of the muscle fibers as well as the number of myonuclei are higher compared with non-athlete control subjects, and the gradient of the correlation curve suggests that the myonuclear domains are higher in large type II muscle fibers of powerlifters compared with those of untrained individuals (364). The use of anabolic steroids and testosterone in powerlifters results in a disproportional increase in muscle size and number of myonuclei, thereby leading to a larger myonuclear domain (365). Although these data indicate a certain degree of myonuclear domain plasticity that could be explained by the reserve capacity of the myonuclei to boost transcriptional output (366), in most cases the myonuclear domains are still within the reported range, also highlighting individual heterogeneity.

In summary, strength-trained athletes have a pronounced increase in muscle volume largely underpinned by the specific hypertrophy of type IIA fibers and accompanied by an increase in pennate angle, resulting in substantial gains in force-generating capacity. In addition to the elevated muscle volume, the fascicles of sprint athletes are elongated, contributing to higher shortening velocity of the muscle and greater power generation. Although neuromuscular adaptations together with muscle hypertrophy explain the enhanced muscle performance in highly trained athletes, these adaptations differ among athletes in a discipline-dependent manner. For example, the musculature of strength-trained athletes has a larger CSA and can produce greater maximal forces, whereas power-trained athletes have slightly lower peak forces but display a faster rate of force development (341). These differences are most pronounced within the first 50 ms of a contraction (341, 367). The enhanced explosive (i.e., high shortening velocity) and maximal forces in athletes result in remarkable power output. In male power athletes, mean peak power, as assessed by a countermovement jump, ranges between 50 and 65 W/kg, with maximal values up to 85 W/kg for men and ∼70 W/kg for women (252, 303, 368, 369). The acceleration power attained at the start of a 100 m sprint was estimated to be 2,392 W or 30.3 W/kg for men and 1,494 W or 24.5 W/kg for women (252). It was calculated that Usain Bolt reached a power output of 2,750 W (29.3 W/kg) during his 100 m world record in 2009 (252). In contrast to these remarkable power metrics for strength/sprint-trained athletes, power output (as measured by jump performance) remains largely unchanged in endurance-trained athletes (266). It is clear that the adaptive response to training stimuli is event-specific in terms of muscle hypertrophy and neuromuscular adaptation, resulting in distinct performance characteristics between different sporting disciplines.

### 3.3. Fiber Type Distribution

**FIGURE 8.**
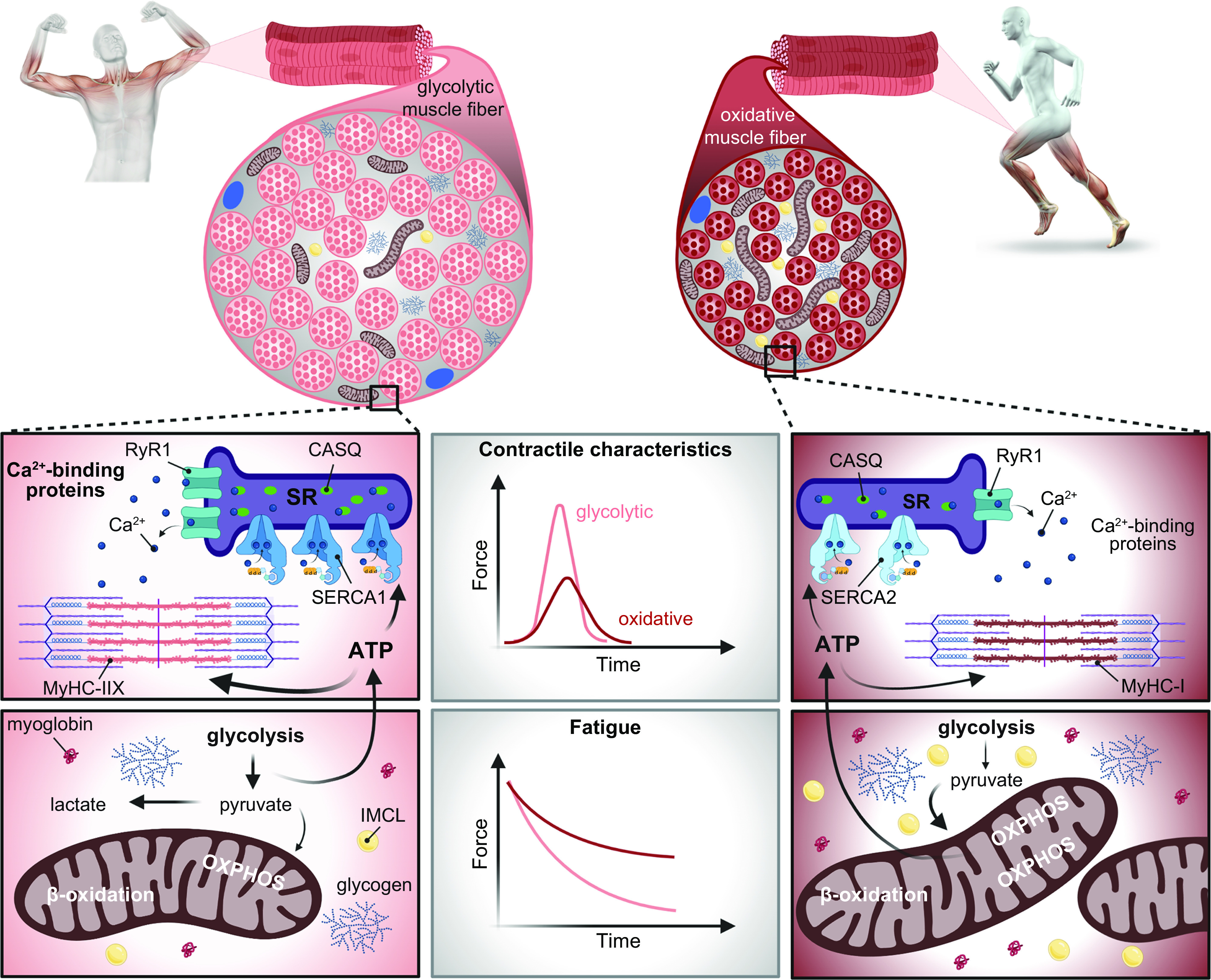
Contractile and metabolic properties of a strength/power-trained and an endurance-trained athlete. Characteristics of fast and powerful muscles of strength/power athletes that are accompanied by a more fatigable muscle as compared with endurance-trained muscles with elevated oxidative capacity that are more fatigue resistant. ATP, adenosine triphosphate; CASQ, calsequestrin; IMCL, intramyocellular lipids; MyHC, myosin heavy chain; OXPHOS, oxidative phosphorylation; RyR1, ryanodine receptor 1; SERCA, sarcoplasmic/endoplasmic reticulum Ca^2+^-ATPase; SR, sarcoplasmic reticulum. Illustrations of people were created by kjpargeter/Freepik. Image created with BioRender.com, with permission.

Distinct properties of the muscle further contribute to the performance of elite athletes, such as fiber type distribution. In addition to distinct innervation and recruitment (as described in sect. 3.2), intrinsic properties of muscle fiber types diverge in a multifaceted manner (155, 357, 370–372). Muscle fiber types can be classified according to their predominantly expressed isoform of the myosin heavy chains, which are the molecular motors of the myofibrils and vary in the relative actin-activated ATPase activity that correlates with contraction velocity (357). Differences in fiber type distribution are accordingly observed in muscles of endurance- or strength-trained athletes and considerably contribute to sports-specific performance. The three myosin heavy chain isoforms expressed in human muscle, type I (encoded by the *MYH7* gene located on chromosome 14, 14q11.2), type IIA (encoded by *MYH2*, on chromosome 17, 17p13.1), and type IIX (encoded by *MYH1*, adjacent to *MYH2* on chromosome 17, 17p13.1), have distinct mechanical properties conferring differences in contractile velocity and force production. Type IIX fibers generate the highest force and have the fastest shortening velocity, resulting in high peak power, and are classified as “fast-twitch” fibers (357, 373). The enhanced force-generating capacity is attributable not only to the larger size of type II fibers but also to intrinsic differences (i.e., higher force-generating capacity of fast myosin heavy chain isoforms and a larger fraction of attached myosin heads), resulting in a higher specific force of type II fibers, which is observed in untrained individuals as well as elite athletes (357, 374–376). In addition to greater power-generating capacity, fast type II muscle fibers exhibit shorter half-relaxation time due to differences in Ca^2+^ transient kinetics (357). In response to an action potential, Ca^2+^ release in fast murine muscle fibers is threefold higher compared with slow fibers, likely because of the greater abundance of the Ca^2+^ release channel ryanodine receptor 1 (RYR1) (357). The inhibitory effect of intracellular Mg^2+^ concentrations on Ca^2+^ release is lower in slow muscle fibers, possibly contributing to the higher fatigue resistance of these fibers, as Mg^2+^ levels rise during fatigue (357). Fiber type-specific differences in Ca^2+^ transient related to the faster decline in cytoplasmic concentrations are determined by the sequestration of Ca^2+^ to binding and buffer proteins such as troponin C (TnC), parvalbumin, and calmodulin, as well as the reuptake of Ca^2+^ into the sarcoplasmic reticulum (SR) by the sarcoplasmic/endoplasmic reticulum Ca^2+^-ATPase (SERCA) pumps (357). As the TnC isoform expressed in fast muscle fibers has four Ca^2+^-binding sites compared with the three in the isoform expressed in slow fibers, Ca^2+^ binding is enhanced in fast muscle fibers. Moreover, the faster uptake of Ca^2+^ by the SR is determined by the increased SR volume and surface area as well as the higher density of SERCA pumps in fast fibers. Of note, the SERCA isoform expressed in fast fibers (SERCA1 vs. SERCA2 in slow fibers) is more sensitive to changes in ADP concentration, which rises during muscle fatigue. As a result, Ca^2+^-pump and -leak rates of SERCA1 are more affected by metabolic stress (i.e., more reduced and more increased, respectively) compared with SERCA2 in slow fibers (357). In addition, calsequestrin (CASQ) that binds Ca^2+^ within the SR is found in greater abundance in fast compared with slow fibers, thereby providing an increased capacity to bind free Ca^2+^. Taken together, differences between Ca^2+^ transient and cross-bridge kinetics in slow and fast muscle fibers contribute to the distinct contractile properties (FIGURE 8). Accordingly, it is not surprising that the energy demand of these fiber types is different during maximal isometric contraction. At rest, energy expenditure in muscle is relatively low, ∼0.008 mM/s of ATP turnover, and mainly used for the Na^+^-K^+^-ATPase in the sarcolemma as well as protein synthesis (357). However, as muscles start to contract, the energy demand is substantially elevated by the myosin ATPases of the molecular motor for cross-bridge cycling (∼70% of overall ATP consumption) and by the SERCA pumps for Ca^2+^ reuptake (∼30% of ATP consumption). Hence, during maximal isometric contractions ATP turnover rates in human type I, IIA, and IIX fibers can increase up to ∼1,000-fold to 1.7, 4.7, and 7.2 mM/s, respectively (357). These fiber-specific differences underscore the remarkable capacity of fast muscle fibers to turn over ATP and enable the generation of high peak power.

To meet the distinct energy demands of different sport activities, slow and fast muscle fibers exhibit divergent metabolic properties. Type I fibers are highly oxidative, whereas type IIX fibers are more glycolytic (357), with the fast type IIA fibers of an intermediate phenotype containing high amounts of oxidative as well as glycolytic enzymes. In line with the considerable energy demand of fast glycolytic fibers, glycogenolytic activity is elevated in these fibers, and during short maximal contractions ATP generation via glycolysis is double that of slow muscle fibers (357), with a corresponding rapid rate of fatigue. In contrast, rates of fatty acid oxidation via the β-oxidation pathway are two- to threefold higher in slow fibers, with the lower ATP demand of these fibers met by mitochondrial respiration over prolonged periods (357). The increased oxidative capacity of type I fibers is determined by the higher mitochondrial volume, accounting for 6% of the fiber volume in type I compared with 4.5% and 2.3% in type IIA and IIX fibers, respectively, as well as the greater density of the mitochondrial cristae and enzymatic activity (357). Additionally, the elevated oxygen demand in slow fibers is met by a higher capillary density and 50% increased myoglobin content compared with fast fibers (154, 357). Along with the capillary-to-fiber ratio, the percentage of type I fibers thus is a strong predictor of endurance capacity (377). The capillary density not only provides greater oxygen delivery and energy substrates but also promotes rapid removal of by-products of sustained contractile activity (i.e., ammonia or lactate). Triglyceride stores are substantially higher in type I fibers compared with type II fibers (∼0.5% of the fiber volume in type I fibers compared with <0.1% in type IIX fibers), whereas conflicting results have been reported regarding glycogen stores (279, 357, 378). Although fiber type-specific differences in glycogen content are not always observed between fast and slow muscle fibers (379, 380), several studies report that glycogen concentration in type II fibers is 15–30% higher compared with type I (357, 381–385). It is unclear whether this is due to differences in methodology or whether the training status has an impact on these findings, since the glycogen content is similar in type I and type II fibers in elite athletes (386).

Collectively, the fiber type-specific differences in contractile and metabolic properties reflected by the inverse relationship between force generation and oxidative capacity of type I, IIA, and IIX fibers result in low force-generating, fatigue-resisting fibers and high force-generating, fatigable fibers, respectively. These properties emphasize the important contribution of fiber type distribution to endurance- or strength/power-based activities. In untrained males, the vastus lateralis comprises ∼40–50% type I fibers (254, 256, 278, 387), though the relative fiber type distribution depends on the site of biopsy (388). Elite endurance athletes typically present with >60% type I fibers, with extremes of >90% (169, 254, 256, 279, 280, 389). These cross-sectional data, however, fail to provide evidence of whether prolonged endurance training induces a shift in fiber distribution, or if elite endurance athletes are successful because of innate fiber type patterns.

In contrast to endurance athletes, well-trained strength and power athletes tend to have a fiber type distribution that resembles that of untrained individuals, at least in terms of overall glycolytic (type II) compared with oxidative (type I) fibers, with some extremes toward a lower abundance of type I fibers (278, 303, 368, 390). A significant shift from IIX to IIA fibers has been reported in strength- and power-trained athletes, with the absolute area of fast muscle fibers substantially larger because of specific hypertrophy of type IIA fibers (278, 303). In other studies, a preservation of type IIX fibers in response to sprint or plyometric training has been found, together with a shift from type I to type IIA (372). Accordingly, sprinters seem to have a lower proportion of slow type I fibers (281, 391). As for elite endurance athletes, it is unclear whether differences in fiber type distribution in power athletes and sprinters are due to preexisting fiber type patterns or whether the training-induced shift is affected by different training paradigms. It also remains perplexing how a fiber type shift is brought about in terms of the temporal and spatial coordination: is there a simultaneous shift in metabolic and contractile properties combined with changes in the motor neuron phenotype, or does one follow the other? Collectively, it is evident that the unique characteristics of type I, IIA, and IIX fibers regarding oxidative and force-generating capacities are instrumental for sport-specific demand and thereby contribute to the achieved peak performances in athletes.

### 3.4. Energy Metabolism and Oxidative Capacity

In skeletal muscle, the three main sites for ATP consumption are the Na^+^-K^+^-ATPases of the sarcolemma for membrane excitability, the SERCA pumps of the SR membrane for Ca^2+^ reuptake, and the myosin ATPases for cross-bridge cycling. Because of the increased elevation in ATP utilization by myosin ATPases and SERCA pumps associated with intense contractile activity, the high ATP demand is a major bioenergetic challenge to the contracting myofibers (357). Given that intramuscular ATP stores are relatively small, metabolic pathways responsible for ATP restoration must be rapidly activated such that ATP levels closely match demand. During sprint events lasting <20 s, the creatine phosphate system and anaerobic glycolysis are the main pathways engaged in ATP resynthesis (392). Although the contribution to ATP provision during a 10-s sprint is similar between the creatine phosphate system and anaerobic glycolysis (53% and 44%, respectively), this is substantially changed during a 30-s sprint (23% and 49%, respectively, with 28% from mitochondrial respiration) at a time when phosphocreatine stores are mostly depleted (392, 393). In contrast, to meet the elevated and sustained energy demand for prolonged exercise (several minutes to several hours), large amounts of ATP are generated by aerobic mitochondrial respiration, with the metabolism of glucose, fatty acids, ketone bodies, and lactate resulting in a higher energy expenditure (392). In extreme endurance events such as the Ironman triathlon (3.8 km swimming, 180 km cycling, 42.2 km running), rates of energy expenditure 8- to 10-fold above resting metabolic rate (RMR) can be sustained for 10–15 h (394). To enable such performances, adequate oxygen uptake, delivery, and extraction concomitant with skeletal muscle ATP generation using large amounts of fatty acids must be tightly coordinated.

In addition to the higher $\dot{\text{V}}{\text{O}}_{2\max}$ values observed in elite endurance-trained athletes, these individuals also have greater efficiency or economy of motion (i.e., a lower oxygen cost at any given work rate or speed of movement) combined with a greater fractional utilization of $\dot{\text{V}}{\text{O}}_{2\max}$ (i.e., the ability to use a greater proportion of their higher $\dot{\text{V}}{\text{O}}_{2\max}$). Collectively, these attributes are associated with increased rates of fatty acid oxidation, a slower rate of depletion of muscle glycogen stores, and enhanced lactate kinetics (395, 396). For example, maximal rates of whole body fatty acid oxidation in elite athletes are twofold higher at submaximal workloads compared with untrained individuals (0.6 g/min vs. 0.3 g/min) (254) and can be reached at much higher intensities (both relative and absolute) compared with untrained individuals (∼45–60% vs. ∼35% of $\dot{\text{V}}{\text{O}}_{2\max}$). A “retooling” of the muscle by adaptation to a high-fat diet while undertaking vigorous training can more than double these rates in world-class endurance-trained athletes to values exceeding 1.5 g/min at work rates of ∼70% of $\dot{\text{V}}{\text{O}}_{2\max}$ (397). During exercise exceeding 80% of $\dot{\text{V}}{\text{O}}_{2\max}$, the availability or rate of appearance of fatty acids in the circulation limits the oxidation of fat-based fuels by skeletal muscle (398). In line with a superior ability to oxidize fat- and spare carbohydrate-based fuels during exercise, the onset of blood lactate accumulation, fixed at a concentration of 4 mM (sometimes arbitrarily defined as “lactate threshold”) (255), is observed at both higher relative and absolute exercise intensity in endurance-trained athletes compared with untrained individuals (80, 257). Therefore, in addition to the higher $\dot{\text{V}}{\text{O}}_{2\max}$ and improved capillary density, training-induced adaptations in skeletal muscle (i.e., the ability to oxidize fat-based fuels and spare muscle glycogen along with a concomitant reduction in lactate appearance) enable elite endurance athletes to sustain high absolute work rates or speeds and resist fatigue for prolonged periods (80, 267, 395). These adaptive changes are discussed in sects. 3.4.1 and 3.4.2.

#### 3.4.1. Mitochondrial adaptations.

During prolonged submaximal exercise with adequate oxygen availability, ATP is synthesized by OXPHOS in the electron transport chain located in the cristae of the inner mitochondrial membrane. To optimize mitochondrial bioenergetics and meet the increased energy requirements during prolonged exercise, highly coordinated adaptive processes enhance the quality as well as the quantity of mitochondria. For example, mitochondrial number and morphology are altered by replication of mitochondrial DNA (mtDNA), synthesis of mitochondrial proteins, transport and incorporation of nuclear-encoded proteins into the corresponding substructures and supercomplexes of mitochondria, and dynamic fusion and fission events (399). Mitochondrial dynamics are essential to maintain quality and are sensitive to physiological and pathological stimuli (400). Whereas fusion leads to elongated mitochondrial tubular networks (401), fission induces fragmentation of the mitochondria allowing the sequestration of damaged or dysfunctional organelles by mitophagy (discussed in sect. 4.6.2) but also mitochondrial biogenesis (402). More specifically, fission at the periphery of the mitochondria enables the degradation of damaged components of the network, whereas fission at the midzone of the mitochondria seems to be instrumental for the proliferation of mitochondria, which cannot be generated de novo (403). The fusion of the outer and inner membrane of the mitochondria is regulated by the transmembrane proteins mitofusion 1 (MFN1) and MFN2 and optic atrophy 1 (OPA1), respectively (401), and important proteins involved in the fission process include dynamin-related protein 1 (DRP1), mitochondrial fission 1 (FIS1), and mitochondrial fission factor 1 (MFF1) (399, 402). How mitochondrial dynamics are altered in muscles of highly trained athletes is not completely understood (404), and most data are based on changes in the abundance of proteins regulating fission and fusion dynamics (289, 405). It has been suggested that fission is elevated after acute exercise to promote the removal of damaged organelles, whereas fusion events are increased during the recovery phase (399, 400, 406), leading to the elongation of mitochondria (407). A functional mitochondrial reticulum is important for ATP generation as well as energy distribution within muscle cells (408). In line with these observations, mitochondria of oxidative muscle fibers are highly interconnected, forming substantially larger mitochondrial networks, whereas glycolytic fibers have more fragmented mitochondria (407, 409). Recently, it has been suggested that the two distinct subpopulations of mitochondria (subsarcolemmal and intermyofibrillar) are physically connected, building a large mitochondrial network (410, 411). These interconnected mitochondrial networks enable a rapid exchange of various factors between mitochondria to ultimately improve ATP production in a spatially coordinated manner (402). As demonstrated in murine muscles, the enrichment of complex IV in subsarcolemmal regions near capillaries facilitates the generation of the proton motive force in oxygen-rich areas whereas the higher abundance of complex V within the myofiber helps to generate ATP near the site of high energetic demand (412). Elongated mitochondrial networks are related to enhanced oxidative metabolism (400), whereas fragmented mitochondria are less efficient in generating ATP (402). This dynamic remodeling of mitochondria could make a substantial contribution to improved mitochondrial quality and function in trained muscle.

Closely linked to changes in mitochondrial dynamics, mitochondrial biogenesis plays a major role in the adaptive response to endurance training, with mitochondrial volume density being strongly correlated to $\dot{\text{V}}{\text{O}}_{2\max}$ (254, 285, 413). The exercise-induced pathways including Ca^2+^ signaling and metabolic, oxidative, and heat stress that are instrumental for mitochondrial adaptation are discussed in sects. 4.2.1, 4.4, 4.5.1, 4.5.2, and 4.6. In the vastus lateralis muscles of untrained individuals, mitochondria comprise ∼4–5% of the muscle volume, the highest being in type I and the lowest in type IIX fibers (255, 289, 300, 302, 414). In endurance-trained athletes, mitochondrial volume density is 50% higher and citrate synthase activity is elevated by 74% (254, 285). Accordingly, mitochondrial respiration is substantially enhanced in elite athletes (415). The exercise training-induced increase in mitochondrial volume density occurs in all fiber types, including IIX, and ranges between 10% and 60% depending on the training impulse (414, 416). In addition to the greater mitochondrial volume density, accompanied by an absolute increase in crista surface, crista density is also higher in skeletal muscle from elite athletes (290), resulting in a further elevation of muscle respiratory capacity. Thus, besides improving oxygen delivery to the mitochondria, muscles can enhance respiratory capacity to meet the high energy demand during exercise by increasing mitochondrial and crista density as well as optimizing the interconnected mitochondrial networks.

#### 3.4.2. Lipid and glycogen storage in muscle.

Skeletal muscle is a major site for both glucose (in the form of glycogen) and lipid (intramyocellular triglycerides) storage. Endurance, but not strength/power, training substantially increases the size of these depots: lipid content is approximately twofold higher in the trained musculature of endurance athletes, which, in part, facilitates the higher rates of fatty acid oxidation (417, 418). Although lipid droplet size is similar in muscle from trained and sedentary individuals, the total intramyocellular lipid pool is substantially higher in endurance-trained muscle. This is due to the combination of an increased number of lipid droplets along with a greater proportion of type I muscle fibers that have a greater capacity for lipid storage than type II fibers (417, 419, 420). These droplets can be categorized into subsarcolemmal or intermyofibrillar lipids, and whereas an elevated fraction of subsarcolemmal lipid droplets is associated with insulin resistance (421), endurance-trained individuals predominantly store lipids in the intermyofibrillar fraction, often in close proximity to mitochondria, which favors high turnover kinetics and confers insulin sensitivity (417, 422), a phenomenon termed the “athlete’s paradox” (423).

Glycogen, a branched glucose polymer, is found in three distinct subcellular compartments, with the majority (75–85%) located between the myofibrils (intermyofibrillar) near the SR and the mitochondria (424). Between 5% and 15% of the glycogen granules are located beneath the sarcolemma (subsarcolemmal) and 5–15% between the contractile filaments within the myofibrils (intramyofibrillar) (424). Whereas type I fibers have more subsarcolemmal and intramyofibrillar glycogen, type II fibers are enriched in intermyofibrillar glycogen (424). The fiber type-specific location of glycogen favors the functional characteristics of type I and II fibers (i.e., fatigue resistance and fast contraction time, respectively) (351). Intermyofibrillar glycogen content is associated with faster relaxation time and, at least in the type II fiber, is necessary to sustain the high rates of ATP turnover by the SERCA pumps (351). In contrast, intramyofibrillar glycogen is correlated with Ca^2+^ release from the SR and fatigue resistance (256, 425). Accordingly, in type I fibers of endurance-trained athletes, intramyofibrillar glycogen content is 60–65% greater, and in both fiber types subsarcolemmal and intermyofibrillar glycogen is increased by 60–65% and 20–25%, respectively, compared with non-athletes (351). During prolonged exercise to fatigue, muscle glycogen concentration decreases in all subcellular compartments, with a preferential depletion of intramyofibrillar glycogen (underlying molecular signals are described in sects. 4.1 and 4.5.1). As pre-exercise muscle (and liver) glycogen content is strongly correlated with prolonged (>90 min) submaximal exercise capacity, higher muscle glycogen in trained muscle plays an important role for maximal performance (386, 424, 426). In addition, glycogen stores are not only replenished more rapidly in trained muscle but also to a greater extent (“supercompensation”) compared with untrained muscles (described in sect. 4.8.3) (427, 428). The restoration of muscle glycogen can be accelerated by high carbohydrate availability in the first 3 h after exercise and can reach rates of synthesis of >10 mmol/kg wet muscle weight/h (428). Despite the capacity for high rates of fatty acid oxidation during submaximal exercise, when highly trained athletes compete in endurance events lasting up to 3 h carbohydrate-based, not fat-based, fuels are the predominant energy substrate for the working muscles. Accordingly, carbohydrate and not lipid availability becomes rate limiting for performance in this context (194).

### 3.5. Muscle Memory

Skeletal muscle mass, $\dot{\text{V}}{\text{O}}_{2\max}$, and other endurance training-induced adaptations rapidly decline upon cessation of a training stimulus (i.e., detraining, deconditioning), with many structural, metabolic, and performance-related parameters returning to pre-training values within weeks to months, even in athletes with a lifelong history of training. However, prior strength training seems to facilitate the regain of muscle mass, surpassing the gains that were achieved when training was commenced from the naive state (429). This phenomenon is referred to as “muscle memory” (430, 431) and, in part, is based on motor learning, intra- and intermuscular coordination, prior experience of body perception, resilience to give into pain and fatigue, and anticipation of exertion. In addition to these central mechanisms, there is evidence for a cellular memory in muscle fibers (432). According to the myonuclear domain theory, myonuclei should be lost during detraining-induced muscle atrophy. However, at least in animal models, the number of myonuclei remains elevated after a short period of disuse despite a loss in muscle mass (433). Thus, the greater myonuclear density and high transcriptional potential could facilitate muscle growth during retraining, contributing to muscle memory. In humans, this phenomenon has received little scientific enquiry, and it is not known whether muscle memory exists, or for how long accrued myonuclei might be preserved (361). A large interindividual heterogeneity of the number of myonuclei elevated after detraining has been reported, and in most cases the pre-training number of myonuclei is similar to that after detraining (434). Of note, athletes with a history of abuse of testosterone or other anabolic steroids, which boost myonuclear accretion, could still benefit from the elevated number of myonuclei even after doping cessation, leading to a potential unfair advantage in competitions long after bans have been served (435).

Although the enhanced growth rates of muscle mass during retraining suggest the presence of some kind of muscle memory (430), many of the training-induced adaptations in muscle are the result of the complex transcriptional response to repeated bouts of exercise, which necessitates accessible genomic regions for the transcriptional machinery. An open chromatin state is generally indicative of enhanced transcriptional activity (360), with the accessibility of chromatin associated with modifications of nucleotides in the DNA and posttranslational modification of histone proteins (histone code) (436, 437). Transcriptionally silent genes exhibit closed, condensed chromatin (heterochromatin), an enrichment of hypermethylated DNA (5-methylcytosine instead of cytosine), deacetylated histones, and methylation of distinct histone residues [e.g., histone 3 lysine residue 9 (H3K9) or H3K27]. Gene transcription requires a state of open chromatin (euchromatin), linked to demethylated DNA, acetylated histones, and the methylation of other histone residues (e.g., H3K4 or H3K26). Besides stable epigenetic markers that can be passed on to the next generation, DNA methylation also occurs as a dynamic process and is influenced by numerous stimuli including habitual level of physical activity, nutrient availability, and (psychological) stress (360, 435, 438). Accordingly, promoter regions of genes involved in metabolic pathways, myogenic processes, or oxidative stress responses are hypomethylated and more accessible in lifelong physically active compared with inactive men (439). Inversely, the promoter region of the peroxisome proliferator-activated receptor (PPAR) γ coactivator 1α (PGC-1α) is hypermethylated, and thus less accessible, after bedrest and associated with reduced mRNA expression (440). In recent years, a growing body of evidence suggests the involvement of altered chromatin landscape in response to exercise training as a possible contributor to muscle memory (360, 430). For example, after endurance or resistance training, widespread changes in DNA methylation status have been reported in muscle (438). Thus, exercise-responsive genes such as PGC-1α are hypomethylated before their induction in response to an acute bout of high-intensity endurance exercise (441). Despite these observations, the contribution of DNA methylation to training adaptation and muscle memory is controversial. In some studies, endurance training promoted the demethylation of genes involved in angiogenesis or oxidative metabolism, associated with increased gene transcription (442, 443), whereas in others there was little effect of HIIT or resistance training on DNA methylation (444). A recent study reported that despite divergent contractile stimuli (HIIT, endurance and resistance exercise), changes in DNA methylation and mRNA expression in skeletal muscle were largely confined to the late (4–8 h) recovery period and similar between the different exercise challenges (445). Many of the discrepancies between investigations can likely be explained by the timing of the biopsy (446), with time course studies suggesting that some DNA methylation changes are retained for up to 48 h after the last training bout (442, 443), returning to baseline levels after 72 h (444). It is not known whether DNA methylation changes are retained after detraining and thereby contribute to muscle memory. Finally, the difference of the transcriptional response of an untrained compared with a trained muscle and the involvement of epigenetic changes therein are currently unexplored.

In contrast to endurance training, the evidence for long-term epigenetic remodeling triggered by resistance training is more robust. In response to a training protocol of loading, unloading, and reloading, several CpG islands remained hypomethylated during unloading, likely contributing to the elevated transcriptional response of the associated genes during reloading (430). These results suggest that chronic changes in DNA methylation may contribute to the transcriptional memory (430), although only a small fraction of the differentially methylated genes displayed a distinct expression pattern (443, 447). Nevertheless, epigenetic regulation of a few regulatory genes might be sufficient to induce a faster and/or greater response to recurring challenges.

Collectively, our understanding of the epigenetic changes in a trained state and the contribution to muscle memory is rudimentary. Findings pertaining to epigenetic remodeling in human muscle are heterogeneous regarding the training protocols employed, the study design/methodology, and the caliber of subjects under investigation. Individuals described as “low responders” to a training intervention may show attenuated epigenetic modification compared with “high responders,” although further work is needed to corroborate this hypothesis (448). In elite athletes, data on epigenetic profiles are almost exclusively from circulating blood cells but not skeletal muscle (449, 450). However, the presence of polymorphisms of genes encoding proteins involved in DNA methylation in elite athletes implies a possible epigenetic predisposition (451).

### 3.6. How Are Physiological and Cellular Training Adaptation Brought About?

Many of the training-induced morphological, biochemical, physiological, and functional adaptations are the culmination of long-term (weeks to months) exposure to training stimuli (FIGURE 6). Many of the transcriptional changes that underpin these adaptations are the transient effect of repeated bouts of acute exercise that accumulate over time and result in new steady-state transcript and protein levels (87, 198), even though disparate outcomes for transcript and proteins can occur (452). For example, the transcription of many mitochondrial genes is transiently induced after a single bout of endurance exercise, leading to mitochondrial biogenesis, improved mitochondrial function, and elevated oxidative metabolism when the stimuli are repeated over time (87, 405). This response, however, is not uniform across all transcripts and the proteins they encode. For example, in contrast to the change in trained muscle, robust transcriptional regulation of myosin heavy chains is not observed after acute exercise bouts (453), whereas other genes show an attenuated expression after repeated exercise exposure (438). Few studies have simultaneously investigated contraction-induced changes in mRNA levels and subsequent training-induced changes in protein levels in human skeletal muscles following chronic interventions, and it is clear that exercise-induced increases in mRNA levels do not always precede increases in the proteins they encode (405, 452, 454). Clearly, several mechanisms regulate the training response/adaptation, and in the case of transcriptional networks there may be additive or attenuated responses over time. In sect. 4 we discuss our current understanding of the molecular mechanisms that are involved in the acute response of muscle to endurance or resistance exercise bouts.

## 4. ACUTE MOLECULAR MECHANISMS UNDERPINNING ENDURANCE- AND RESISTANCE-BASED EXERCISE

In this section, the sensors and major signaling pathways involved in the response of skeletal muscle to a single bout of endurance and resistance exercise are summarized. We review the downstream effects of these stimuli (e.g., transcriptional regulation and translational control) that promote muscle adaptations in response to two distinct training paradigms. In addition to the well-described pathways such as Ca^2+^-dependent pathways, AMP-activated protein kinase (AMPK), and mammalian target of rapamycin (mTOR) complex 1 (mTORC1) signaling reviewed above, we delineate the important roles of other transducers such as mechanosensing and transduction, for which there is a scarcity of data on athletic populations. A brief discussion of the common signaling pathways activated by both endurance and resistance training is provided, followed by a comprehensive overview of the molecular events that occur after an acute bout of exercise and underpin the differential responses to divergent contractile stimuli described in sects. 2 and 3.

**FIGURE 9.**
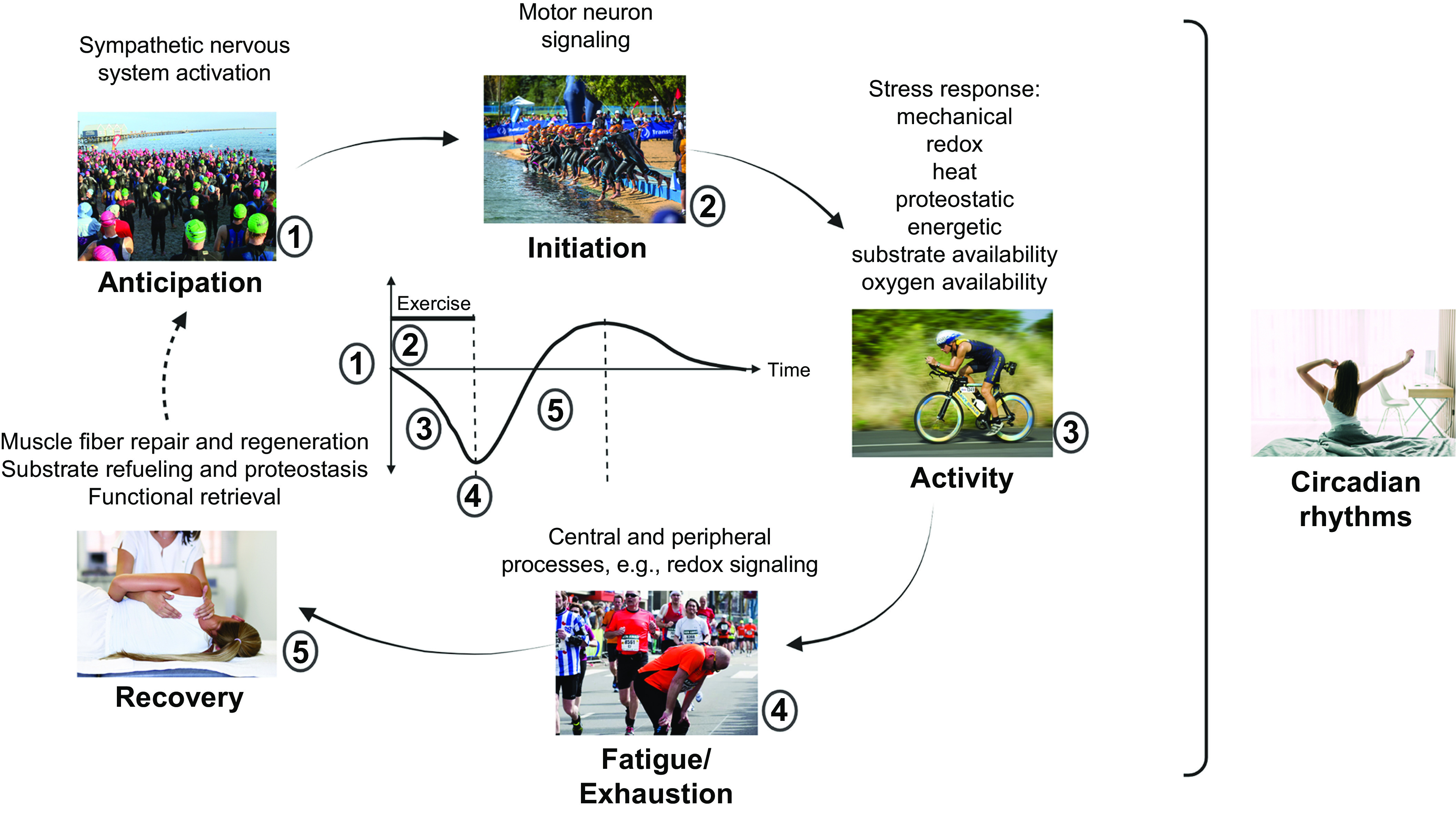
Temporal engagement of different pathways and processes in skeletal muscle in exercise. Anticipation of activity is linked to elevated sympathetic nervous system tone. Motor neuron firing triggers muscle contractions at the start of and during an activity. While contracting, muscle will be affected by different stressors and the related reaction/mitigation, for example, mechanical stress, reactive oxygen and nitrogen species (redox) production, heat, altered proteostasis and protein unfolding, metabolic changes, substrate availability, and oxygen provisioning. The exact temporal sequence of engagement of and interactions between these pathways are unknown. Different mechanisms contribute to muscle fatigue, exhaustion, and exercise cessation. Subsequently, muscle repair, regeneration, and refueling are important for functional retrieval. Many of these processes are modulated by circadian input. Images: triathlon anticipation from Wikimeda Commons (CC-BY-SA 3.0, creator: Wiech), triathlon start from Wikimedia Commons (CC-BY-SA 2.0, creator: IQRemix), triathlon cycling from PxHere.com (CC0 Public Domain), exhaustion from Wikimedia Commons (CC-BY-SA 4.0, creator: Wallco26), massage from Freepick.com (author: javi_indy), waking up from Freepik.com (author: diana.grytsku).

A single bout of exercise sets in motion a complex program of interconnected signaling events, along with the activation of numerous biochemical pathways and transcriptional networks that orchestrate the spatio-temporal responses to muscle contraction and coordinate a pleiotropic response in other tissues and organs to control energy substrate provisioning, oxygen availability, and heat dissipation (9). These perturbations are initiated by numerous inputs, which can occur in parallel, overlap, or be completely independent. Several critical regulatory “nodes” provide the hub for signal integration and subsequent control of transcription and enzymatic activity (9, 55, 455). Repeated bouts of exercise over several weeks or months result in a continuous modulation of this response and, over time, ultimately contribute to provoke chronic adaptations (456). Although the molecular mechanisms underpinning many of the chronic responses to exercise training remain undefined, numerous insights regarding acute exercise response have been described in recent years (9, 39, 41, 55, 163, 393, 455, 457). A caveat is that although the focus of this review pertains to data obtained from exercise-trained humans, many of our current mechanistic insights have originated from rodent and other in vivo and in vitro model systems. It is important to make a distinction between voluntary, whole body in vivo responses to exercise and those elicited by other experimental models. Ex vivo electrical stimulation of an isolated skeletal muscle, for instance, evokes an action potential and “contraction” and triggers intracellular pathways with putative roles in training adaptation. However, whole body, voluntary exercise induces a range of additional physiological responses that are critical for muscle performance (and movement). Accordingly, many effects observed in animals and isolated systems can differ from those seen in humans in vivo, and care should be taken when extrapolating responses from one set of conditions or a given experimental model to another (9). Here, we describe the signaling pathways and mechanistic events that are principally involved in the response to an acute exercise bout and culminate in the subsequent training adaptation. Mechanisms that are important in muscle atrophy and pathological situations are not discussed in detail, and because of space limitations we cite recent reviews that serve as starting points for further reading and collections of primary literature. The following sections are structured based on the putative engagement of the respective pathways in muscle contraction, from pre (anticipation)- to peri (start of activity, during the exercise bout)- to post (muscle fatigue and exercise cessation and finally recovery, repair, and regeneration)-exercise (FIGURE 9). It should be noted, however, that the exact temporal sequence of engagement and the interactions between and integration of these processes are not fully understood.

### 4.1. Neuroendocrine Signaling in the Anticipatory Phase and During Muscle Contraction

**FIGURE 10.**
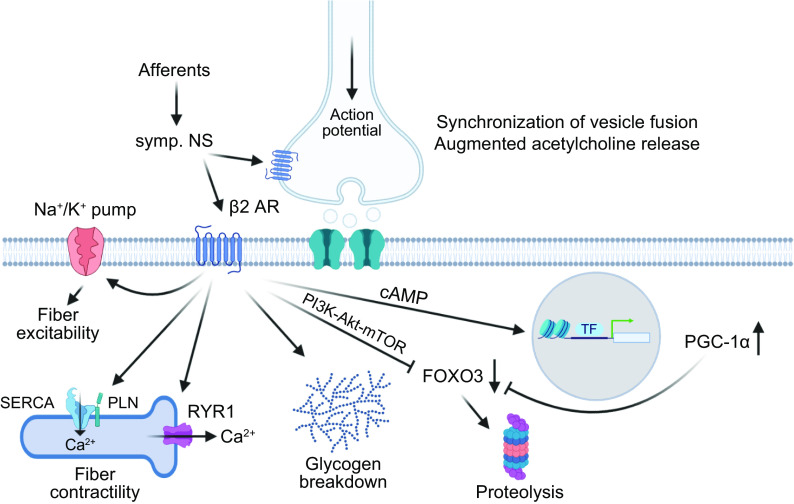
Neuroendocrine signaling by the sympathetic nervous system in exercise anticipation and muscle contraction. Sympathetic activation of the motor neuron and skeletal muscle cells results in modulation of fiber excitability and contractility, metabolic and proteostatic remodeling, and the activation of a transcriptional program. These events prepare muscle cells for upcoming contractions and help to maintain contractile activity upon engagement. β2 AR, β_2_-adrenoreceptors; Akt, protein kinase B; cAMP, cyclic AMP; FOXO3, forkhead transcription factor O3; mTOR, mammalian target of rapamycin; PGC-1α, peroxisome proliferator-activated receptor γ coactivator 1α; PI3K, phophoinositide 3-kinase; PLN, phospholamban; RYR1, ryanodine receptor 1; SERCA, sarcoplasmic/endoplasmic reticulum Ca^2+^-ATPase; symp. NS, sympathetic nervous system; TF, transcription factor. Image created with BioRender.com, with permission.

An acute bout of exercise represents a “one-off” stressor to whole body homeostasis, provoking widespread perturbations in numerous cells, tissues, and organs that are caused by or are a response to the increased metabolic activity of contracting skeletal muscles (9, 55, 455). Induction of the “fight or flight” response, including activation of the sympathetic nervous system in parallel with the motor system, responds to feedback from the exercise pressor reflex via group III/IV skeletal muscle afferents (458, 459). This reflex encompasses feedback that is evoked from mechanically (muscle mechanoreflex) and metabolically (muscle metaboreflex) sensitive afferents during contractions, leading to parasympathetic depression and sympathetic activation (460). Consequently, blood flow to skeletal muscle is increased as a result of elevated heart rate, blood pressure, and rate of ventilation. The exercise pressor reflex can be complemented by “central command,” in which stimulation of medullary and spinal circuitries by higher brain centers likewise evokes respiratory and cardiovascular modulation (460). Importantly, the sympathetic nervous system can also be engaged by anticipation and other emotional factors preceding motor activation. Exercise leads to a substantial increase in circulating catecholamines, a response that is greater in trained compared with untrained individuals exercising at the same relative intensity (458, 459). In part, centrally controlled modulation of the sympathetic nervous system is required for a systemic activation of events that support muscle contraction, such as increased pulmonary and cardiovascular output or various metabolic pathways to liberate energy substrates. Importantly, muscle tissue is also innervated by the sympathetic nervous system through the activation of β_2_-adrenoreceptors by catecholamines, with epinephrine having a higher affinity than norepinephrine for these receptors, and a higher density of β_2_-adrenoreceptors on type I compared with type II muscle fibers (458, 459). Besides affecting the microvasculature, the adrenergic system exerts other functions in this tissue, including direct effects on the neuromuscular system (FIGURE 10) (458, 459). Initially, adrenergic action on the presynaptic side of the NMJ helps synchronize neurotransmitter vesicle fusion and augment acetylcholine release. Then, β_2_-adrenoreceptor action on the muscle fibers activates the Na^+^-K^+^ pump and thereby fiber excitability, potentially attenuating fatigability. Muscle contractility, in particular twitch force and relaxation rate, is modulated by the adrenergic effect on Ca^2+^ release and reuptake via RYR1 and phospholamban, the latter of which is exclusively expressed in slow muscle fibers. Metabolically, β_2_-adrenoreceptor activation antagonizes insulin to stimulate glycogen breakdown and inhibit glycogen synthesis. Moreover, β_2_-adrenoreceptor agonism represses proteolytic processes, leading to a transient anabolic effect on muscle mass. As members of the G protein-coupled transmembrane receptor family, β_2_-adrenoreceptors engage numerous signaling pathways and effectors in muscle cells (461). The exchange of GDP for GTP at the α stimulatory subunit of guanine nucleotide-binding regulatory protein (Gαs) results in an activation of adenylate cyclase, the cyclic AMP (cAMP) signaling pathway, and ultimately elevated transcriptional activity of the cAMP response element-binding protein (CREB), which stimulates the modulation of additional control genes, including PGC-1α, or myogenic factors such as myoblast determination protein 1 (MyoD) (461). The inhibition of the Forkhead box 3 (FOXO3) by PGC-1α, and hence of protein degradation and fiber atrophy (462), is potentiated by the effect of the Gβγ subunit of the β_2_-adrenoreceptor, which modulates the activity of phosphoinositide 3-kinase (PI3K) and, via downstream activation of protein kinase B (PKB/Akt), exerts a negative effect on FOXO3 function as well as a positive modulation of protein synthesis by activation of mTORC1 (461).

### 4.2. Motor Neuron Activation of Muscle Fiber Contractions

**FIGURE 11.**
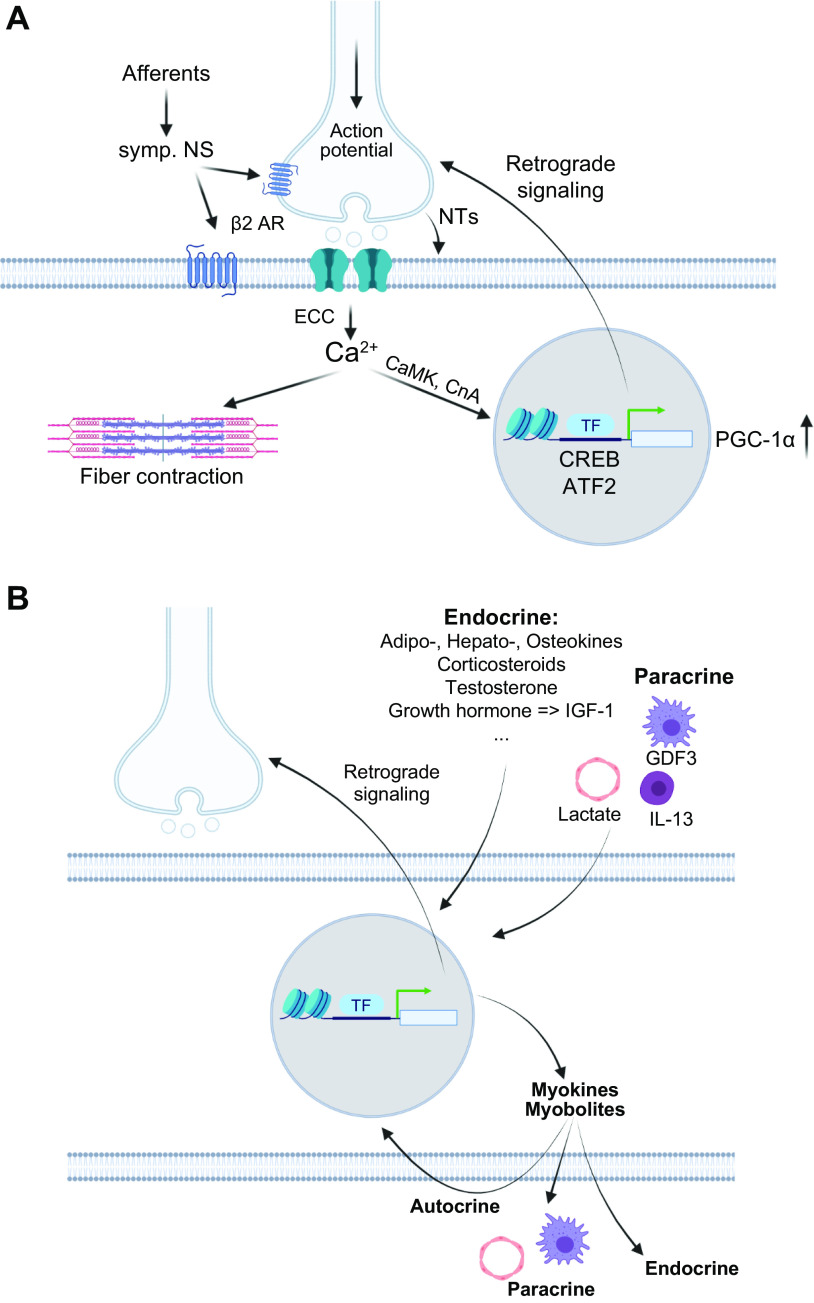
Motor neuron signaling and (neuro)endocrine effectors of contraction. *A*: motor neuronal signaling triggers excitation-contraction coupling and thereby evokes a rise in intramyocellular calcium (Ca^2+^), which enables fiber contractions, activates various signaling pathways, and modulates a transcriptional response, including retrograde feedback to the motor neuron. Motor neuron activity is modulated by sympathetic tone and includes various neurotrophic factors besides the neurotransmitter acetylcholine. *B*: exerkines, originating from tissues including muscle (myokines), liver (hepatokines), adipose tissue (adipokines), and bone (osteokines) as well as other hormones coordinate a systemic response to contractile activity. Many of these factors exert auto-, para-, and endocrine effects. In addition, signals can be propagated by exercise-linked changes in different metabolites (myobolites or myometabokines). β2 AR, β_2_-adrenoreceptors; ATF2, activating factor 2; CaMK, calcium/calmodulin-dependent protein kinase; CnA, calcineurin A; CREB, cAMP-responsive element binding protein; ECC, excitation-contraction coupling; GDF3, growth differentiation factor 3; IGF-1, insulin-like growth factor 1; IL-13, interleukin 13; NTs, neurotrophic factors; PGC-1α, peroxisome proliferator-activated receptor γ coactivator 1α; symp. NS, sympathetic nervous system; TF, transcription factor. Image created with BioRender.com, with permission.

Muscle fiber contraction can be initiated via different mechanisms: neuronal activity in the motor cortex for voluntary movement, sensory neuronal input for involuntary reflex contractions such as elicited in proprioceptive or vestibular control, or hypothalamic activation of thermogenesis-promoting neurons for shivering (463–465). Regardless of the origin of the signal and upstream circuitry, neuronal input converges on α-motor neurons in the ventral horn of the spinal cord (466). These motor neurons, their descending axons, and the innervated muscle fibers form a motor unit that transforms synaptic input into muscle contractions (318). Motor units differ in size, with one motor neuron interfacing with from a handful to many thousands of individual muscle fibers (318). Collectively, the motor units of one muscle are referred to as the motor unit pool (318). At the synapse between motor neurons and muscle fibers, the NMJ, an axonal action potential, results in the release of the neurotransmitter acetylcholine, which, after traversing the synaptic cleft, binds to nicotinic acetylcholine receptors that cluster at the entry points of subneural clefts on the muscle membrane (FIGURE 11*A*) (467, 468). Activation of these ligand-gated ion channels results in influx of Na^+^ and a process called ECC, leading to depolarization of the muscle membrane and, on the intracellular side of T tubules, the release of Ca^2+^ from the SR, which ultimately initiates and maintains muscle contractions (469). Thus, one of the first events to occur in response to a single bout of exercise is initiation of Ca^2+^-dependent signaling.

#### 4.2.1. Ca^2+^ signaling.

Besides binding to troponin C and initiating the interaction between actin and myosin, intracellular Ca^2+^ activates a multitude of signaling pathways in myofibers, modulating numerous physiological functions (470). For example, Ca^2+^ signaling is associated with the control of glycolysis, mitochondrial function, and the rates of protein synthesis and degradation (471). The different Ca^2+^ transients that are evoked by activation of muscle fibers by slow and fast motor neurons contribute to the specificity of different muscle fiber types (472, 473). Mechanistically, the protein phosphatase calcineurin A (CnA) and Ca^2+^/calmodulin-activated protein kinase II (CaMKII) are Ca^2+^-activated mediators involved in controlling gene transcription linked primarily to a slow fiber phenotype (471). In this context, CnA and CaMK activities converge on the cAMP-dependent binding protein (CREB) and activating transcription factor 2 (ATF2, also called CREB2), which are activated by phosphorylation and dephosphorylation of respective Ser/Thr sites, subsequently binding to the promoter of the PGC-1α gene PPARGC1A and inducing transcription of this coregulator protein, among others (474). Once synthesized, PGC-1α competes with histone deacetylase 5 (HDAC5) to coregulate myocyte enhancer factor 2 (MEF2) family members on its own promoter and, using this positive autoregulatory loop, ensures robust transcriptional expression (475, 476). Consistent with elevated levels in slow and exercised muscle fibers, PGC-1α mediates a broad remodeling of skeletal muscle, resulting in a slow-twitch, oxidative, fatigue-resistant phenotype (477) that also includes extramyofiber adaptations such as at the microvasculature (478) or the presynaptic side of the NMJ (315). Mice with a skeletal muscle-specific ablation of PGC-1α display abnormal glucose and insulin homeostasis (479), contraction-induced fiber damage, impaired endurance capacity, and other characteristics indicative of pathological inactivity (480).

The mechanisms that underlie the broad integration of a vast number of signaling pathway that are engaged in contracting muscle, and which mediate a tightly choreographed modulation of broad transcriptional programs by PGC-1α, are unclear. Gene expression from different promoters and transcriptional start sites (474, 481, 482), various isoforms (481, 482), context-specific posttranslational modifications (474, 482), and the RNA binding-dependent assembly of specific multiprotein-containing transcriptional complexes and DNA regulatory elements in sequestered nuclear condensates (483) could all contribute to a coordinated spatio-temporal control of PGC-1α-mediated network control (482, 484). Indeed, PGC-1α functionally and/or physically interacts with multiple transcription factors and coregulators that affect the exercise phenotype of skeletal muscle in a dynamic manner (482, 485, 486). Thereby, spatial specification, and the expression of synaptic genes in subsynaptic but not extrasynaptic myonuclei (487), or a temporal specification, such as the activation of catabolic and anabolic pathways like fatty acid β-oxidation and de novo lipogenesis to increase intramyocellular lipids (488), might be achieved (474, 489). Of note, Ca^2+^ signaling also activates a number of additional transcription factors with unclear epistatic relationship to PGC-1α, including family members of the nuclear receptor 4A (NR4A) family, of which NR4A2/NURR-1 and NR4A3/NOR-1 in turn regulate target genes involved in oxidative metabolism and improved muscle endurance (490, 491). As Ca^2+^ signaling is activated in response to both endurance- and resistance-based exercise, it is still unclear whether and how this pathway is affected in a training mode-specific manner. For example, training intensity and duration may be one of the factors that influence Ca^2+^ fluctuations in the muscle and thereby influence Ca^2+^-mediated signaling.

#### 4.2.2. (Neuro)endocrine factors, exerkines, and myokines.

Besides neurotransmitter-mediated activation, motor neurons and skeletal muscle fibers engage in a bidirectional cross talk through several secreted factors (FIGURE 11*B*). Some of these, such as motor neuron-derived agrin, are important for the development and stabilization of the NMJ (468). This synapse, however, also exhibits remarkable plasticity in the mature state, with exercise providing a strong stimulus to alter morphology and function (312). Neuregulin-1 is secreted by the motor neuron and activates signaling pathways in the muscle fiber that lead to the phosphorylation of PGC-1α and the GA-binding protein B [GABPB, also called nuclear respiratory factor 2b (NRF-2b)] subunit of the GABP transcription factor complex (487). These local events, which primarily affect subsynaptic myonuclei close to the NMJ, could provide the mechanistic basis of the spatial specification of PGC-1α to exclusively regulate the transcription of postsynaptic NMJ genes in these, and not extrasynaptic, nuclei (487). Intriguingly, active muscle PGC-1α triggers a remodeling process of the NMJ that extends to the presynaptic side with altered mitochondrial and synaptic vesicle numbers in the active zone and a quantal content reflecting slow NMJ function (315). These observations go beyond the current view of a unidirectional control of muscle fiber type by the respective activity patterns of motor neurons, in that elevation of muscle PGC-1α, observed in slow-type or exercised fibers, affects motor neuron function, at least at the NMJ. Neurturin could be a mediator of this PGC-1α-dependent retrograde signaling, acting on the NMJ (492) and even on motor neurons in the spinal cord (493). Another example of such a factor, brain-derived neurotrophic factor (BDNF), a signaling factor secreted from neurons but also cells and tissues such as skeletal muscle fibers (494), functions as a myokine, a hormonal entity produced and secreted by muscle cells. BDNF expression is elevated upon contractile activity, the protein secreted, and besides a potential effect on the motor neuron affects NMJ morphology and function postsynaptically in an autocrine manner (495).

Several other (neuro)endocrine factors have also been identified in the context of exercise. For many of these, particularly testosterone and other classical steroid and non-steroid hormones, regulation during an acute exercise bout is probably of little significance compared with the chronic effects such as restoration of substrate stores, muscle repair and regeneration, and in extreme situations overtraining/overreaching. For example, corticosteroids, glucagon, and leptin are elevated when blood glucose and/or muscle and liver glycogen concentrations are low and stimulate fatty acid oxidation in muscle by activation of AMPK (496). Testosterone, growth hormone, and the downstream target insulin-like growth factor 1 (IGF-1) are induced after different types of resistance training and exert anabolic effects by stimulating MPS and fiber repair (497). The regulation of these hormones in humans is variable, and little is known about the exact molecular mechanisms that mediate these adaptations. Indeed, gain- and loss-of-function models of the receptors of some of these hormones fail to reveal a clear picture regarding their function in exercise-induced muscle plasticity (486). Besides these classic hormones, a novel class of so-called “myokines” have been discovered and studied in recent years (498). Myokines, some of which are only produced in contracting muscle fibers, sometimes referred to as “exerkines,” elicit auto-, para-, and endocrine effects to coordinate local and systemic processes (499, 500). For example, when secreted as a myokine, interleukin (IL)-6 acts as a metabolic coordinator by promoting lipolysis in adipose tissue, gluconeogenesis in the liver, and, via activation of AMPK, glucose uptake and fatty acid oxidation in muscle (501). Paracrine effects of myokines are also instrumental for an adequate response of muscle tissue to exercise, such as the proangiogenic effects of IL-6 or the vascular epithelial growth factor (VEGF) on epithelial cells (501) or the activation of resident macrophage polarization by the B-type natriuretic peptide (BNP) and secreted phosphoprotein 1 (SPP1) (502, 503). Many of the endurance exercise-induced myokines are under the transcriptional control of PGC-1α (501, 504), whereas the regulation of those modulated by resistance training is less clear (504, 505). Paracrine signaling from different cell types to myofibers is also important for a normal exercise response, such as the exercise-induced secretion of IL-13 by type 2 innate lymphoid and other immune cells that promotes an oxidative phenotype in muscle (506) or growth differentiation factor 3 (GDF3) by macrophages (507), as well as endothelial cell-secreted lactate, both important for muscle regeneration (508). A complex dialogue between muscle and other cell types, mediated by myokines, hepatokines, adipokines, and osteokines, ensures proper and coordinated local as well as systemic adaptations to contractile activity, such as for the adiponectin-mediated activation of muscle AMPK (509, 510). Collectively, however, our understanding regarding hormones that affect skeletal muscle during contraction or throughout recovery and regeneration from exercise is rudimentary.

### 4.3. Mechanosensing and Mechanostress Mitigation

#### 4.3.1. Cell membrane mechanosensing.

**FIGURE 12.**
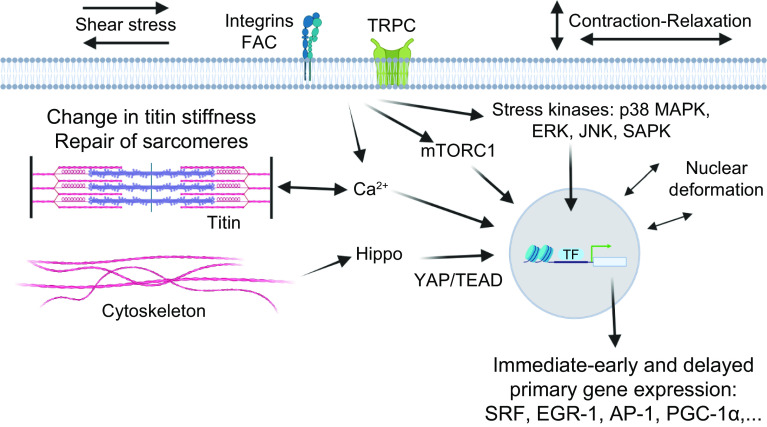
Mechanosensing and mechanostress mitigation in the contracting muscle fiber. Mechanical stress is sensed and translated by structures at the cell membrane and intramyocellular components in the cytosol, cytoskeleton, sarcomeres, or nucleus. As a consequence, resistance to shear stress is increased, stiffness and integrity of sarcomeric structures adapted, and a broad program of immediate-early and delayed primary genes initiated. AP-1, activating protein 1; EGR-1, early growth response gene 1; ERK, extracellular signal-regulated kinase; FAC, focal adhesion complex; JNK, c-Jun NH_2_-terminal protein kinase; mTORC1, mammalian target of rapamycin complex 1; p38 MAPK, p38 mitogen-activated protein kinase; PGC-1α, peroxisome proliferator-activated receptor γ coactivator 1α; SAPK, stress-activated protein kinase; SRF, serum response factor; TEAD, TEA domain transcription factor; TF, transcription factor; TRPC, mechano-gated Ca^2+^ transient receptor potential channels; YAP, yes-associated protein. Image created with BioRender.com, with permission.

Mechanical stress is exerted on muscle fibers at the initiation of and during exercise by passive stretching, sarcomeric contraction, and other stimuli (FIGURE 12) (511). The force-induced stretch and contraction/compression of muscle fibers in situ is not restricted to the longitudinal direction but extends across orthogonal, radial, and tangential axes (511). The ensuing shear, tension, and compressional stress and cellular deformation present a high potential for damage to the extracellular matrix (ECM), cell membrane, intracellular scaffolds, and other structures. Therefore, a complex system of mechanosensing exists to attenuate damage and initiate adaptive processes that confer protection against acute and subsequent insults. Broadly, mechanical stress is detected by different structures at the cell membrane or by intracellular sensors. Stretch-activated channels (SACs) belong to the first category in skeletal muscle, of which the mechano-gated Ca^2+^ transient receptor potential channels (TRPCs) are activated by mechanical stress (512). The expression and function of the mechanosensing cation channel Piezo1 in skeletal muscle is less understood, at least in intact fibers (513). TRPC activity results in Ca^2+^ influx, and the ensuing elevations in intramyocellular Ca^2+^ either directly affect target structures or, through the activation of distinct signaling pathways, result in the activation of calmodulin, CnA, MEF2, and nuclear factor of activated T cells (NFAT) (512). Dephosphorylation of NFAT by CnA enables a cytoplasmic-nuclear translocation and subsequent regulation of gene expression by this transcription factor, often together with MEF2 (512). NFAT binds to regulatory genes involved in muscle fiber type remodeling or hypertrophy, including induction of PGC-1α and, via a positive feedback loop, transcription of TRPCs, thereby sensitizing muscle fibers to future mechanoinsults (512). Non-genomic effects of intracellular Ca^2+^ include direct modulation of structural proteins such as titin or actin. Deformation of cytoskeletal structures thus mediates mechanosensing by receptors at the cell membrane, leading to a regulatory loop that reacts to changes in mechanical stress by adapting sensing and cell rigidity (512). Ca^2+^, for example, affects the assembly of actin filaments, thereby driving a cytoskeletal rearrangement that affects cell stiffness (512). Increases in intracellular Ca^2+^ also result in the activation of Ras guanine nucleotide-releasing factors (GRFs), which in turn activate the Ras GTPase (514). In this manner, the mitogen-activated protein kinase (MAPK) signaling pathway is engaged, leading to elevated activity of p38 MAPKs, extracellular signal-regulated kinases (ERKs), c-Jun NH_2_-terminal protein kinases (JNKs), and stress-activated protein kinases (SAPKs). The MAPK pathway integrates TRPC Ca^2+^-derived mechanosensing via transmembrane adhesion receptors such as integrins, integrin-associated proteins, focal adhesion kinase (FAKs), and other components of the focal adhesion complex (512, 515). The stress-activated kinases then execute the phosphorylation of enzymatic effectors and transcriptional regulators including PGC-1α (516), serum-response factor (SRF), JUN, and FOS, forming the activating protein 1 (AP-1) complex or early growth response gene 1 (EGR-1), and thereby inducing the expression of immediate-early and delayed primary response genes (517). This program includes several transcription factors needed for secondary response gene transcription, some of which are directly modified by stress kinase phosphorylation (518). Collectively, these early primary and secondary response genes promote processes related to cell survival, cytoskeletal rearrangement, and elevation of small heat shock proteins (HSPs) and other chaperones, thus mitigating potential harmful events in the contracting muscle fiber on different levels (511, 512, 518).

#### 4.3.2. Cytosolic mechanosensing.

Besides these mechanosensing systems originating at the cell membrane, at least two cytosolic signaling pathways are important. First, mTORC1 activity is increased in mechanically stimulated fibers (519, 520). This protein kinase is the major regulator of cellular protein synthesis and one of the key inhibitors of autophagy, thereby controlling cell size (521). In prohypertrophic states, mTORC1 is activated and contributes to the regulation of muscle mass (522) in a controlled manner. Dysregulation of muscle mTORC1 through ablation of the upstream inhibitor tuberous sclerosis complex (TSC) results in an atrophic phenotype (523). In contrast, inducible, skeletal muscle-specific ablation of raptor, an essential component of mTORC1, has little effect on muscle mass in sedentary mice (524). These findings suggest that mTORC1 is an important, but not the only, regulatory factor governing skeletal muscle hypertrophy (525). The prototypical activators of mTORC1 signaling are insulin and amino acids, in particular leucine, arginine, and glutamine, collectively representing an anabolic context with high nutrient availability (521). However, in muscle contraction and mechanosensing, other stimuli may be more important to activate mTORC1, although the specific exercise-stimulated mechanisms have not been identified (519). Candidate pathways include IGF-1 signaling or that of the muscle-specific IGF-1 splice variant mechano growth factor (MGF), whose activity is increased by mechanical stimulation and could engage mTORC1 via PI3K-Akt-dependent pathways. However, even though IGF-1 exerts clear anabolic effects in muscle, experimental evidence indicates that acute mechanosensing and activation of mTORC1 might be independent of PI3K and Akt (519, 520). mTORC1 activation could also be achieved by cross talk with established mechanosensing pathways such as intracellular Ca^2+^ engaging mTORC1 via activation of CaMK kinase α (CaMKKα) or phosphorylation of TSC and raptor by ERK kinases (519, 520). Furthermore, different membrane-bound phospholipases and diacylglycerol kinase ζ (DGKζ) are activated by stretch, in part via Ca^2+^, and then exert various functions related to the mechanoresponse (519). Phospholipases activate the Raf-ERK as well as PI3K-Akt pathways, thereby regulating two potential upstream regulators of mTORC1 (519). Moreover, phosphatidic acid produced by phospholipase D or DGKζ directly stimulates mTORC1 activity, most likely by binding to the 12-kDa FK506-binding protein (FKBP12)-rapamycin binding (FRB) domain of mTOR (519). Then, a mechanosensitive integrin-FAK-TSC2-Ras homolog enriched in brain protein (RHEB) axis could also converge on mTORC1 (515). Finally, several other mechanisms have been proposed to mediate the activation of mTORC1 upon mechanical stress and loading, including translocation of TSC2, the cellular redox state, in particular reactive nitrogen species (RNS), and amino acid availability. Regardless of the mode of upstream control, mTORC1 activity triggered by loading-induced mechanical stress results in an upregulation of protein synthesis and other hypertrophic programs and, in combination with PI3K-Akt activity, a reduction in catabolic processes and apoptosis (519).

In parallel to mTOR signaling, the Hippo pathway also contributes to mechanostress-induced muscle cell remodeling (526). The transcriptional coactivator Yes-associated protein (YAP) is controlled by the Hippo pathway and is activated upon deformation or rearrangement of actin and the subsequent regulation of various actin-associated proteins (526). After cytoplasmic-nuclear translocation, YAP coactivates TEA domain (TEAD) transcription factor family members to control the expression of genes linked to decrease in apoptosis and increase in muscle hypertrophy (526). Even though the prohypertrophic function of YAP is mTOR independent, the Hippo/YAP pathway may engage in cross talk with the Akt/mTOR pathway to promote muscle mass gains and with the transforming growth factor-β (TGF-β)/small worm phenotype/Mothers Against Decapentaplegic (SMAD) pathway to coordinate repair and regeneration, respectively (526).

#### 4.3.3. Structural mechanosensing at the cytoskeleton, sarcomeres, and cell nucleus.

Intramyocellular structural components also contribute to mechanosensing, mostly filamentous actin and the sarcomeric proteins myosin and, in particular, titin, a giant scaffold protein that is essential for forming the sarcomeric structure (527–529). Because of reversible extension of specific domains, titin acts as a molecular spring and determines the passive elastic properties of sarcomeres and muscle fibers and can even contribute to active force and tension generation during muscle contractions (530). During stretch, physical deformation of titin leads to the release of several ankyrin-repeat proteins including cardiac ankyrin-repeat protein (CARP), diabetes-related ankyrin-repeat protein (DARP), and ankyrin-repeat-domain protein-2 (ANKRD2) that act as transcription factors to initiate sarcomerogenesis and fiber repair leading to adaptive muscle remodeling and increased expression of structural proteins, including titin, desmin, and dystrophin (527, 528). At that time, a complex formed by the muscle LIM protein (MLP) is released from titin and interacts with NFAT signaling to induce prohypertrophic genes. The titin-cap (T-CAP) activates the E3-ubiquitin ligase mouse double minute 2 homolog (MDM2), which, together with the titin-associated Ca^2+^-dependent cysteine proteases calpain1 and calpain3 as well as the neighbor-of-BRCA1-gene-1 (NRB-1), promotes protein degradation and autophagy, hence a robust system of protein quality control to repair defective sarcomere structures (527, 528). In the active muscle, in addition to constituting one of the major signaling hubs for mechanosensing, titin represents a tunable spring, acquiring context-dependent stiffness and thereby conferring enhanced tension and an increased passive force to muscle cells (527, 528). Different posttranslational modifications of the titin protein and direct binding of Ca^2+^ affect the stiffness of this protein to improve sarcomere integrity during exercise and promote the effectiveness of accelerated contraction-relaxation cycles (527, 528).

Other intracellular structural components that are involved in mechanosensing and transduction include the cell nucleus (531, 532), often the most stiff element of the cell (533). The perinuclear cytoskeleton transmits forces to the Linker of Nucleoskeleton and Cytoskeleton (LINC) complex and various proteins of the nuclear envelope such as lamins and emerin. The LINC complex provides a mechanical connection between the cyto- and nucleoskeleton. Remodeling of the nuclear lamina by mechanical force can directly affect gene expression by changing the chromatin condensation state, histone modifications, gene repositioning, and the binding of transcription factors to accessible DNA regions. Inversely, the chromatin state and histone modifications might affect nuclear rigidity. Thereby, a bidirectional link between nuclear mechanosensing and transcriptional regulation is achieved (532, 533). How this is affected by exercise is unclear. Signaling induced by mechanosensing occurs as soon as the muscle contracts, but it is not known whether resistance- and endurance-type exercise affect mechanosensing in a similar fashion and thereby activate the same pathways. Furthermore, it is possible that specific interventions or types of contractions (i.e., eccentric vs. concentric contractions) may enhance stretch or shear stress and thereby promote mechanosensing-induced signaling pathways.

### 4.4. Oxidative Stress

**FIGURE 13.**
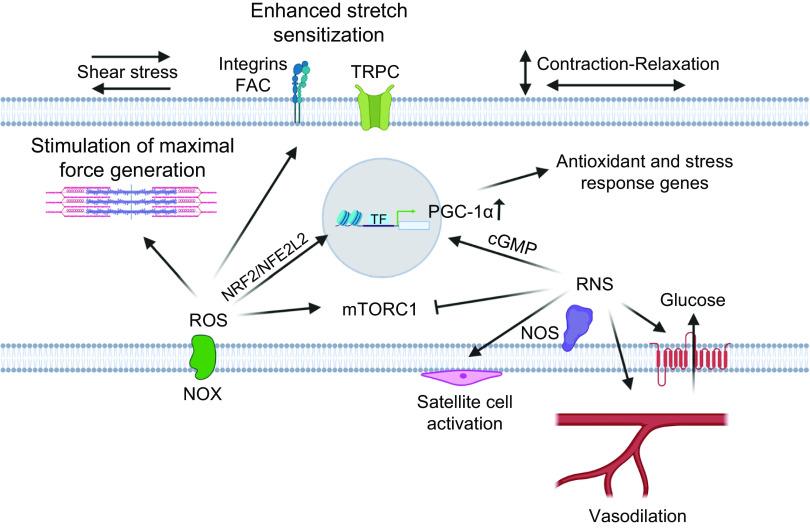
Redox stress by reactive oxygen and nitrogen species in muscle contraction. Redox regulation of muscle contractility and stress response by reactive oxygen (ROS) and nitrogen (RNS) species during muscle contraction. ROS and RNS are primarily produced by enzymes at the muscle cell membrane. Subsequently, a number of downstream effects are promoted, including an increase in energy substrate and oxygen availability, enhancement of force generation, improvement of the resilience against oxidative stress, and modulation of a transcriptional program for muscle remodeling. cGMP, cyclic guanylate monophosphate; FAC, focal adhesion complex; mTORC1, mammalian target of rapamycin complex 1; NOS, nitric oxide synthase; NOX, NADPH oxidase; NRF2/NFE2L2, nuclear factor erythroid-derived 2-like 2; PGC-1α, peroxisome proliferator-activated receptor γ coactivator 1α; TF, transcription factor; TRPC, mechano-gated Ca^2+^ transient receptor potential channels. Image created with BioRender.com, with permission.

Oxidative stress and the associated cellular responses are closely linked to mechanical stress and mechanosensing (FIGURE 13) (534). In contracting skeletal muscle cells, reactive oxygen species (ROS) are produced by NADPH oxidase enzymes NOX2 and NOX4 and to a lesser extent by mitochondria. Whereas NOX4 seems to be a more constitutive enzyme important for basal ROS production in muscle, NOX2 activity is highly regulated by specific agonists such as angiotensin II or various cytokines. At the onset of contraction, phospholipase A_2_ is activated by elevated intracellular Ca^2+^ to produce arachidonic acid from the cleavage of membrane phospholipids. Arachidonic acid in turn increases ROS production by NOX2 and mitochondria (534). Muscle stretch, compression, or osmotic shock thereby results in a rapid burst of intramyocellular ROS, which then serves as physiological signaling agent (535). In addition, NOX2 activity might be directly modulated by mechanosensing of the intracellular microtubule network (536). NOX2-derived ROS in turn affect TRPCs and, in a positive feedback loop, further sensitize muscle fibers to stretch. In a specific range of concentration, ROS are important to stimulate maximal force generation, even though the mechanistic aspects of this function are unclear. RNS could trigger adaptations similar to ROS in skeletal muscle. Nitric oxide (NO) is primarily produced by the type I neuronal nitric oxide synthase (nNOS) enzyme in the contracting muscle fiber (537, 538). Subsequently, NO affects several systems in myofibers, either by direct nitrosylation or by activation of NO-dependent guanylate cyclases and the ensuing increase in cGMP (536–539). NO has a broad-ranging impact on muscle cell metabolism by increasing glucose uptake or reducing the activities of creatine kinase and several glycolytic enzymes (536–539). Together with NO produced by epithelial (eNOS) and inducible (iNOS) NOS, positive effects on vasodilation and the activation of satellite cells are achieved. Finally, both ROS and RNS engage numerous signaling pathways and transcriptional regulators to promote a myocellular remodeling (540). MAPK signaling pathways are redox sensitive, and an activation of JNK, p38 MAPK, and ERK is observed upon elevated levels of ROS and RNS (537). In terms of transcriptional regulation, release of the redox-sensitive Kelch-like ECH-associated protein 1 (KEAP1) enables a cytoplasmic-nuclear shuttling of the transcription factor nuclear factor erythroid 2-related factor 2 (NRF2, also known as nuclear factor erythroid-derived 2-like 2, NFE2L2), which then binds to antioxidant response elements (AREs) on regulatory sites of various genes to promote a robust antioxidant response (541). NRF2/NFE2L2 also induces the expression of nuclear respiratory factor 1 (NRF1) and PGC-1α and increases mitochondrial biogenesis and substrate oxidation (541). NRF2/NFE2L2 gene expression is controlled by an AMPK-PGC-1α axis, implying a robust mutual regulation of NRF2/NFE2L2 and PGC-1α, with both PGC-1α and NRF2/NFE2L2 being activated by MAPK pathway effectors (541). Furthermore, PGC-1α regulates the transcription of several genes encoding antioxidant enzymes, at least in part, in an NRF2/NFE2L2-dependent manner (541). PGC-1α gene transcription is also controlled by NO via the activation of cGMP-associated signaling, potentially in a bimodal fashion (541). Upon redox-dependent oxidation of cysteine residues (540) and a resulting stabilization of the tumor suppressor gene p53, this transcription factor binds to and activates the promoter of PGC-1α to mediate some of its protective effects in muscle during exercise (542). Nuclear factor κB (NF-κB) and AP-1 are two additional transcription factors that can be activated in a redox-specific manner (537). In this context, NF-κB is a strong regulator of antioxidant genes, whereas AP-1 induces a more general stress response (537). Of note, both transcription factors interact with PGC-1α: AP-1, together with MEF2 family factors, controls PGC-1α gene expression, whereas PGC-1α binds to AP-1 to regulate some of its target genes (474, 537). In contrast, a mutual negative interaction between NF-κB and PGC-1α may help to regulate homeostasis between metabolism and inflammation in muscle (543). The cellular redox state also affects PI3K-Akt-mTOR signaling to control rates of protein synthesis and muscle hypertrophy (534, 539). First, ROS-mediated activation of Akt results in an increase in mTORC1 function (534), whereas NO exerts a negative effect on this pathway, presumably to keep hypertrophy in check (539). Both redox modalities merge in the production of peroxynitrite, formed by the reaction of superoxide with NO, which leads to an activation of mTORC1 (534). Thus, the redox balance modulates and fine-tunes muscle hypertrophy. Collectively, adequate regulation of ROS and RNS is instrumental to react to mechanical and other types of stress during exercise and acutely affects contractility (534). Furthermore, such a contraction-linked increase in ROS and RNS triggers a hormetic response, in which an upregulation of mitochondrial uncoupling proteins, antioxidant enzymes, and compounds blunts potential damage by future insults (534). Accordingly, higher levels of antioxidant enzymes such as superoxide dismutase (SOD) 1 and 2, catalase (CAT), and glutathione peroxidase (GPx), as well as non-enzymatic antioxidants such as reduced glutathione (GSH) that ameliorate redox homeostasis, are observed in the muscles of trained individuals (544–547). Based on the role of these processes, inhibition of the production of ROS and RNS with pharmacological and/or nutritional antioxidants could be detrimental in promoting an optimal cellular environment to maximize training adaptation (548). Even though excessive ROS production has been linked to tumorigenesis, cardiovascular diseases, hypertension, neurodegenerative disorders, and other chronic pathologies, exercise training substantially reduces the risk of these diseases, despite the contraction-induced acute elevations of ROS and RNS in skeletal muscle (534). These epidemiological observations highlight the importance of a well-balanced and coordinated redox production and detoxification system in muscle, without any apparent pathological consequences in muscle tissue or beyond (549).

### 4.5. Energy Homeostasis, Substrate and Oxygen Sensing

#### 4.5.1. Energy homeostasis and energetic stress.

**FIGURE 14.**
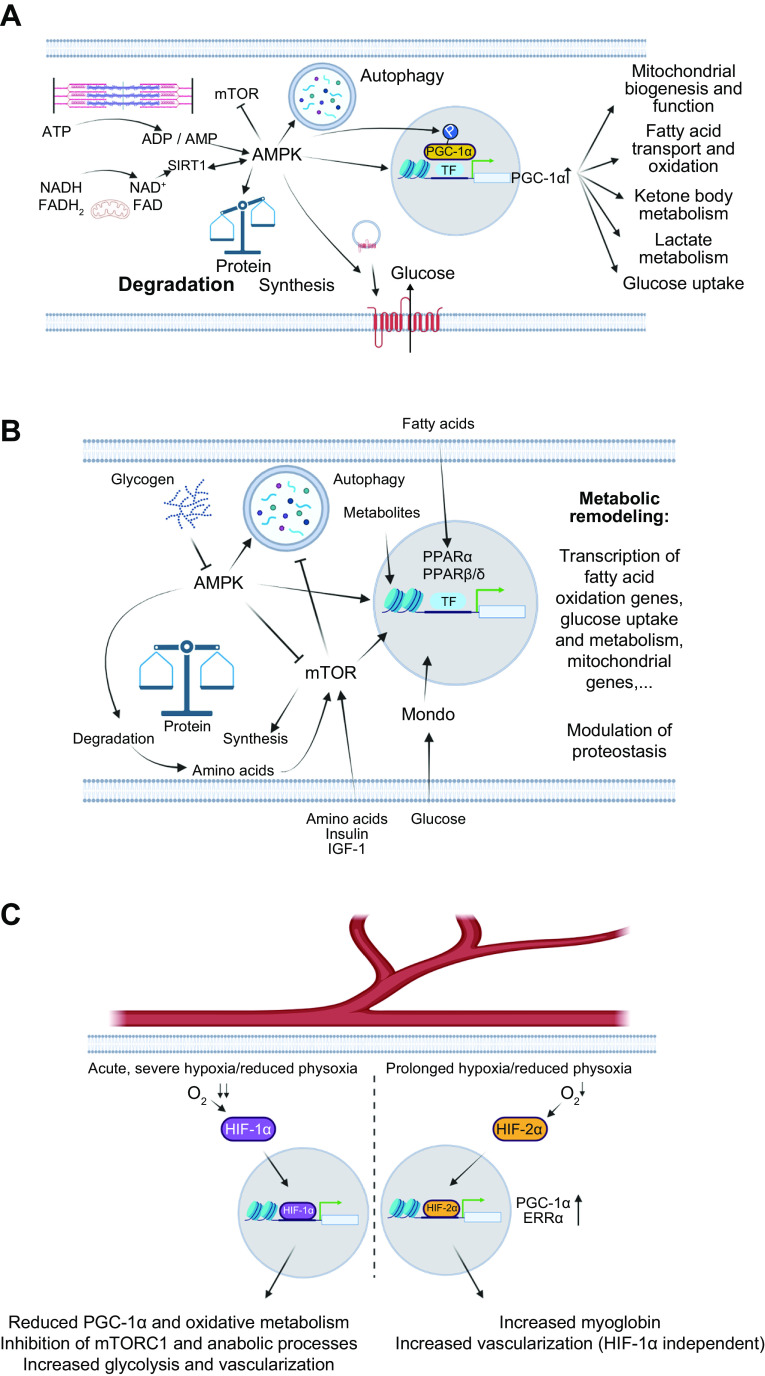
Energy homeostasis, substrate and oxygen signaling. *A*: metabolic stress signaling in muscle contraction to increase energy provisioning. A decrease in energy substrate availability leads to the activation of energy sensors that reduce anabolic processes consuming ATP and induce catabolic pathways to produce more ATP. This broad response comprises direct modulation of protein and enzymatic activities by posttranslational modification as well as the control of a broad transcriptional program. *B*: energy substrate signaling senses substrate levels and leads to metabolic partitioning. Thereby, muscle cell metabolism is coordinated with substrate availability and anabolism balanced with catabolism. *C*: reduced oxygen availability in skeletal muscle is sensed by hypoxia-inducible factor (HIF)-1α and HIF-2α in acute and chronic settings, respectively. HIF-1α rapidly reduces pathways that consume O_2_, while promoting anaerobic glycolysis to generate ATP. HIF-2α promotes muscle oxygen extraction and provisioning. AMPK, AMP-activated protein kinase; ERRα, estrogen-related receptor α; FAD: flavin adenine dinucleotide; IGF-1, insulin-like growth factor 1; mTOR, mammalian target of rapamycin; NAD, nicotinamide adenine dinucleotide; PGC-1α, peroxisome proliferator-activated receptor γ coactivator 1α; PPARα/-β/δ, peroxisome proliferator-activated receptor α/-β/δ; SIRT1, sirtuin 1; TF, transcription factor. Image created with BioRender.com, with permission.

Muscle cell contractions are strongly linked to major metabolic remodeling (FIGURE 14*A*). In many situations, the demand for ATP as a rapid energy source is only partially compensated by creatine phosphate system transfer and the subsequent increase in oxygen-independent anaerobic and aerobic metabolism of glucose, lipids, lactate, ketone bodies, and other energy substrates. This leads to a shift in the relative concentrations of ATP, ADP, and AMP (393). During recovery from strenuous exercise, and after optimal refueling, this catabolic energetic state will pivot into an anabolic state and muscle and liver glycogen as well as intramyocellular lipid stores will be replenished. In parallel to the shift in adenosine species, the relative abundance of nicotinamide adenine dinucleotide (NAD^+^) and NADH as well as other cofactors involved in myocellular redox reactions is affected by mitochondrial OXPHOS, lactate dehydrogenase activity, as well as other processes (550, 551). These fundamental metabolic changes triggered by the increased energetic demand of contracting muscle cells result in a complex modulation of signaling cascades and biochemical pathways that are crucial for training adaptation. For example, a shift in the ATP-to-AMP ratio toward AMP leads to the activation of a signal transduction pathway centered on AMPK, which in turn phosphorylates downstream protein kinase substrates (552, 553). In addition, AMPK activity is modulated by the cellular environment, in particular upstream kinases liver kinase B1 (LKB1) in exercise of short duration (i.e., seconds to minutes) and CaMKKβ during prolonged exercise bouts, or by signaling induced by various myokines such as IL-6, IL-15, BDNF, and leukemia inhibitory factor (LIF) (498, 501, 554). Although the precise role of contraction-induced AMPK signaling in the regulation of glucose uptake and fatty acid oxidation remains controversial (555), AMPK activation leads to a general catabolic response upon cessation of exercise, including augmented uptake of glucose and fatty acids, reduced rates of glycogen synthesis, elevated glycolysis, and improved mitochondrial activity (554). Furthermore, AMPK is a potent activator of autophagy and promotes protein degradation via the ATP-dependent ubiquitin-proteasome system to liberate amino acids for energy production in muscle and gluconeogenesis in the liver (554). The regulation of these two processes is tightly coordinated, with inhibition or activation largely determined by the relative activity of AMPK and mTOR and the balance between the catabolic and anabolic state of the muscle cell (556). For example, ketoacids resulting from the metabolism of the branched-chain amino acids valine, leucine, and isoleucine can be used to generate ATP via the tricarboxylic acid (TCA) cycle in a catabolic state, whereas branched-chain amino acids can also activate mTOR in the face of high energy availability (557). In the former setting, AMPK inhibits mTOR activity by phosphorylation of the mTORC1 upstream inhibitors TSC1 and 2 as well as the mTORC1 component raptor (521). Autophagy is directly stimulated by AMPK-mediated phosphorylation of the Unc-51-like kinase 1 (ULK1) (521, 554), whereas protein degradation is boosted by phosphorylation and thereby activation of FOXO3 (554), as well as transcriptional activation of FOXO1 and FOXO3 (558). In an anabolic context, insulin or amino acid signaling as well as lysosomal recruitment of mTOR strongly reduce autophagy and catabolic pathways so that protein synthesis and lipogenesis are enhanced (521). S6 protein kinase (S6K) and eukaryotic translation initiation factor 4E (eIF4E) are two important mTORC1 phosphorylation substrates that, together with increased transcriptional activity of RNA polymerases I and III, subsequently coordinate ribosomal biogenesis and protein synthesis (521, 559). Insulin signaling-dependent activation of Akt results in phosphorylation and nuclear exclusion of FOXO1 and 3, and thereby inhibition of protein degradation, as well as activation of glycogen synthase kinase 3β (GSK3β), which in turn phosphorylates eIF2B and β-catenin to promote protein synthesis (560, 561). The strong inhibitory effect of mTORC1 on autophagy is exerted by phosphorylation of ULK1 and transcription factor EB (TFEB), a key regulator of lysosomal biogenesis (521, 562). TFEB and TFE3 are activated by Ca^2+^/calcineurin signaling in contracting skeletal muscle, resulting in nuclear translocation and activation of autophagy, lysosomal, and mitochondrial gene expression, in part by functionally interacting with PGC-1α (562). Thus, overall, AMPK and mTORC1 exert opposite functions, promoting catabolic and anabolic processes, respectively, with a direct inhibition of mTORC1 by AMPK; this reciprocal control is important to regulate specific adaptations to endurance- and resistance-based training stimuli but might also be relevant in the context of concurrent training and the training interference effect (163, 521, 563). The molecular basis of the interference effect underlying the compromised strength gains observed when individuals simultaneously undertake both endurance and strength training programs (described in sect. 2) was investigated at the molecular level by Atherton and colleagues (564), who studied isolated rat muscles that were electrically stimulated with either low frequency to mimic endurance exercise (3 h at 10 Hz) or high frequency to mimic resistance training (6 × 10 repetitions of 3-s bursts at 100 Hz). They observed selective activation and/or downregulation of AMPK-PGC-1α or Akt-mTORC1 signaling pathways in response to these divergent loading patterns. Specifically, they reported that electrical stimulation that mimicked either endurance- or resistance-based training switched signaling to either an AMPK‐PGC‐1α- or Akt‐mTOR‐selective state. They termed this activity the AMPK‐Akt “master switch” and hypothesized that such selective activation of AMPK‐PGC‐1α or Akt‐mTORC1 signaling could explain specific adaptive responses to endurance or resistance training (564). However, Coffey et al. (565) demonstrated that in highly trained athletes prior training history attenuated the “exercise-specific” signaling responses involved in single-mode adaptations to training and that a degree of “response plasticity” was conserved at opposite ends of the endurance‐resistance adaptation continuum. Given that genotypes were originally selected to support diverse physical activity patterns obligatory for human survival and that modern-day success in many sporting endeavors requires a high endurance capacity coupled with superior explosive power, the conservation of multiple signaling networks to meet divergent physiological demands seems to make sound evolutionary and biological sense (1).

AMPK and mTOR can also synergize and converge in certain cellular contexts (563), potentially representing the temporal specification of these two proteins in the response to contractile activity of skeletal muscle, or adaptation to mixed or concurrent forms of training. For example, after training, AMPK improves insulin sensitivity of myofibers, thereby potentiating the activation of mTOR (554). Moreover, both AMPK as well as mTOR converge on the activation of PGC-1α to promote mitochondrial biogenesis and oxidative metabolism of energy substrates (474, 521, 554). The interaction between these two key metabolic regulators, and the implications for muscle cell plasticity, are therefore multifaceted and remain poorly understood. In addition to mTOR signaling, AMPK also engages redox-sensitive pathways including the NAD^+^-dependent protein deacetylase sirtuin 1 (SIRT1), with AMPK-mediated phosphorylation of PGC-1α preceding SIRT1-dependent deacetylation and subsequent activation in skeletal muscle (566, 567). Lysine deacetylation by SIRT1, SIRT3, and other NAD^+^ sensors as well as the counterregulatory effect by acetyltransferases such as general control non-repressed 5 (GCN5) are not restricted to enhancing and decreasing the activity of PGC-1α (568) but also other targets, including histones. Thereby, a tight coupling between the myocellular redox state and transcriptional regulation in exercise is facilitated (569, 570). Although the functional interaction between PGC-1α acetylation and AMPK-dependent phosphorylation has been proposed, the cross talk between phosphorylation events by other kinases, protein methylation, ubiquitination, sumoylation, or O-linked β-N-acetylglucosamination is less well understood (474, 482). Posttranslational modifications of proteins can affect different properties, including stability or turnover, localization, interactions with other protein binding partners, DNA recruitment, enzymatic activity, or structural conformation (571, 572). Indeed, once activated, PGC-1α interacts with many different transcription factors, including estrogen-related receptor α (ERRα), NRF1, NRF2/GABP, MEF2C, and MEF2D, to coordinate a complex, yet poorly understood transcriptional network encoding the biological program of endurance exercise adaptation encompassing vascularization, remodeling of the NMJ, or induction of a slow-type contractile phenotype (474, 573, 574). Moreover, PGC-1α initiates a coordinated transcriptional network encoding mitochondrial biogenesis and function, including TCA and OXPHOS, fatty acid uptake, transport, and β-oxidation, ketone body and lactate metabolism, as well as glucose uptake and use in the pentose phosphate pathway (474, 481, 575–577). Other transcription factors such as ERRγ or PPARβ/δ, for which the epistatic relationship to PGC-1α and the involvement in the exercise response are less clear, evoke similar gene programs in skeletal muscle (485). Finally, a multifaceted interaction between PGC-1α and other coregulator proteins affects the activity of PGC-1α in the control of skeletal muscle plasticity. For example, nuclear corepressor 1 (NCOR1) competes with PGC-1α for binding to ERRα and PPARβ/δ, thereby reducing PGC-1α target gene expression mediated by these interactions (486). In addition, transducers of regulated CREB-binding proteins 1, 2, and 3 [TORC1/2/3, also called cAMP-regulated transcriptional coactivators (CRTCs)] are coregulators that are strongly modulated by environmental cues and, in turn, induce PGC-1α gene expression (486). The diversity of interactions and hence the variety in PGC-1α-containing protein complexes with different coregulators and transcription factor binding partners likely contribute to the highly orchestrated and coordinated transcriptional network control exerted by this coactivator protein in skeletal muscle in exercise (474, 481–486, 578).

#### 4.5.2. Signaling mediated by substrates and metabolites.

Metabolites and energy substrates have additional effects on exercise adaptation in skeletal muscle, and even transient and subtle perturbation in cellular homeostasis can trigger broad downstream effects (FIGURE 14*B*) (84). For example, glycogen binds to a carbohydrate-binding module on the AMPKβ subunit to negatively affect AMPK activity (579), providing a molecular explanation for the enhanced training effect when individuals commence exercise with low muscle glycogen stores, as in “train low” (glycogen) protocols (81, 579). Mondo transcription factors are activated by binding of glucose, leading to a modulation of the expression of genes involved in glucose homeostasis by MondoA in muscle, while concomitantly reducing fatty acid oxidation by PGC-1α, thereby providing an important metabolic switch between glucose and lipid oxidation in contracting fibers according to the Randle cycle (580). Fatty acids are ligands for various nuclear receptors, including the PPARs, and liver X receptors (LXRs), leading to an increase in rates of lipid oxidation and lipogenesis in skeletal muscle, respectively (485). Furthermore, as noted above, amino acids are potent activators of mTOR and thus contribute to mTORC1-mediated control of muscle proteostasis that promotes fiber hypertrophy in response to resistance-based training (522). The requirement for mTORC1 in inducing exercise hypertrophy seems to depend on various factors, including temporal aspects or training stimuli, with considerable mTORC1-independent contributions (581). Moreover, amino acid supplementation to activate mTORC1 without concomitant resistance training is clearly insufficient to induce gains in muscle mass and strength (582). Succinate, a citrate cell cycle intermediate, accumulates in muscle, the interstitial space, as well as the circulation during exercise. Signaling triggered by succinate binding to succinate receptor 1 (SUNCR1) induces adaptations in the gene expression programs for axon guidance, neuronal projection, and muscle regeneration that collectively contribute to endurance exercise capacity (583). In addition to NAD^+^, other cofactors at the intersection of metabolism and transcription are likely affected by contractile activity in skeletal muscle, including flavin adenine dinucleotide (FAD) in the TCA cycle and OXPHOS, α-ketoglutarate in the citrate cycle, and acyl-CoA and acetyl-CoA in substrate oxidation and the citrate cycle (393). These cofactors are directly involved in epigenetic gene regulation by modulating histone and DNA demethylation and histone acetylation (584). Whether such an epigenetic coupling to metabolism exists in skeletal muscle during exercise is unknown, even though epigenetic mechanisms contribute to exercise-induced muscle plasticity (578, 585). Finally, the extensive metabolic remodeling in muscle and other tissues during and after exercise is likely to include many other metabolites with important signaling and regulatory functions in the training response (586, 587), collectively referred to as myobolites or myometabokines (588, 589). In addition to muscle-intrinsic effects, inter-tissue communication is also mediated by such circulating metabolites including β-aminoisobutyric acid (BAIBA) that help to coordinate the general systemic response (590). In a similar manner, altered muscle metabolic capacity can affect the circulating levels of endogenous metabolites and, in the case of aberrant levels of such metabolites, thereby contribute to a “detoxification” by lowering hyperketonemia (575) or the conversion of kynurenine into kynurenic acid, which is unable to cross the blood-brain barrier (591).

One of the first studies to propose a link between substrate availability and molecular signaling in exercising human muscle was that of Wojtaszewski and colleagues (592). They measured muscle signaling responses and substrate utilization during and after an acute bout of steady-state cycling in well-trained subjects under conditions in which exercise was commenced with either low or high muscle glycogen content. After exercise started in a low-glycogen state AMPKα2 and AMPKα1 activity was elevated to a greater extent compared to when the same exercise bout was commenced with high glycogen content (592). However, exercise commenced in the lowered glycogen state was associated with elevated catecholamine concentrations compared with the glycogen-loaded trial, making it difficult to determine whether fuel availability and/or humoral factors contributed to the observed boosted AMPKα2 and AMPKα1 activity. Subsequently, the results of several other investigations demonstrated that, compared with normal glycogen levels, commencing endurance exercise with reduced glycogen availability increases the phosphorylation of p38 MAPK and transcriptional activation of IL-6, pyruvate dehydrogenase kinase 4 (PDK4), hexokinase, and HSP72 (188). PGC-1α mRNA was also induced to a greater extent (8- vs. 3-fold) after highly trained cyclists performed a standardized bout of submaximal endurance exercise with low versus normal glycogen concentration (191). Even in response to resistance exercise, commencing exercise with low glycogen seems to promote mitochondrial adaptation, as demonstrated with increased phosphorylation of p53 and mRNA expression of PGC-1α (206). Although “train low” (glycogen) protocols boost the training response in well-trained athletes, this training paradigm failed to be superior to conventional protocols with regard to performance enhancement.

#### 4.5.3. Oxygen sensing.

If oxygen consumption exceeds oxygen availability and uptake, “physiological hypoxia” (or “reduced physoxia”) occurs, corresponding to a drop of mean oxygen tension from 30 mmHg to 2–3 mmHg in contracting skeletal muscle fibers (593–595). This drop is already observed at relatively low exercise intensities, implying that additional mechanisms might be contributing to oxygen availability for different structures in the muscle cells, such as myoglobin function or local intracellular oxygen levels and gradients (596–598). Hypoxia-inducible factor (HIF)-1α and HIF-2α are the major mediators of hypoxic stress (FIGURE 14*C*). Upon reduced oxygen availability, HIF-1α proteolysis by prolyl-hydroxylases 2 and 3 (PHD2/3) is alleviated and the HIF-1α protein stabilized (593). HIF-1α then controls gene programs involved in anaerobic glycolysis to sustain energy production in the absence of adequate oxygen supply and represses those programs that are oxygen dependent, such as mitochondrial OXPHOS. This is brought about by inhibiting PGC-1α expression and activity while promoting gene expression related to vascularization to improve oxygen supply via the myokine VEGF (593). Furthermore, the hypoxia-responsive gene DNA damage inducible transcript 4 (DDIT4) encoding the REDD1/RTP801 protein, an activator of TSC1/2, inhibits mTORC1 and downstream anabolic pathways, thereby limiting ATP-consuming processes (599). The paralog HIF-2α plays a permissive role in the acute hypoxic response compared with HIF-1α but a greater function in long-term adaptation in which HIF-1α activity is repressed (593). In this context, PGC-1α induces the expression of HIF-2α, ERRα, the AP-1 complex, and VEGF to promote angiogenesis and vascularization in a HIF-1α-independent manner (474, 600). With regard to training, the temporary hypoxia in muscle can be exacerbated by vasoconstriction of capillaries in muscle tissue, for example by vascular occlusion or peak contraction (149). Accordingly, the exercise-induced increase in mRNA expression of all four postulated PGC-1α isoforms is blunted when endurance exercise is performed with blood flow restriction (601). However, in the long term, training under hypoxic conditions may boost the adaptive response in the muscle. It remains to be determined whether such training paradigms are more effective in enhancing the performance of elite athletes than training under normoxic conditions.

### 4.6. Thermotolerance, Protein and Organelle Quality Control

#### 4.6.1. Heat stress.

**FIGURE 15.**
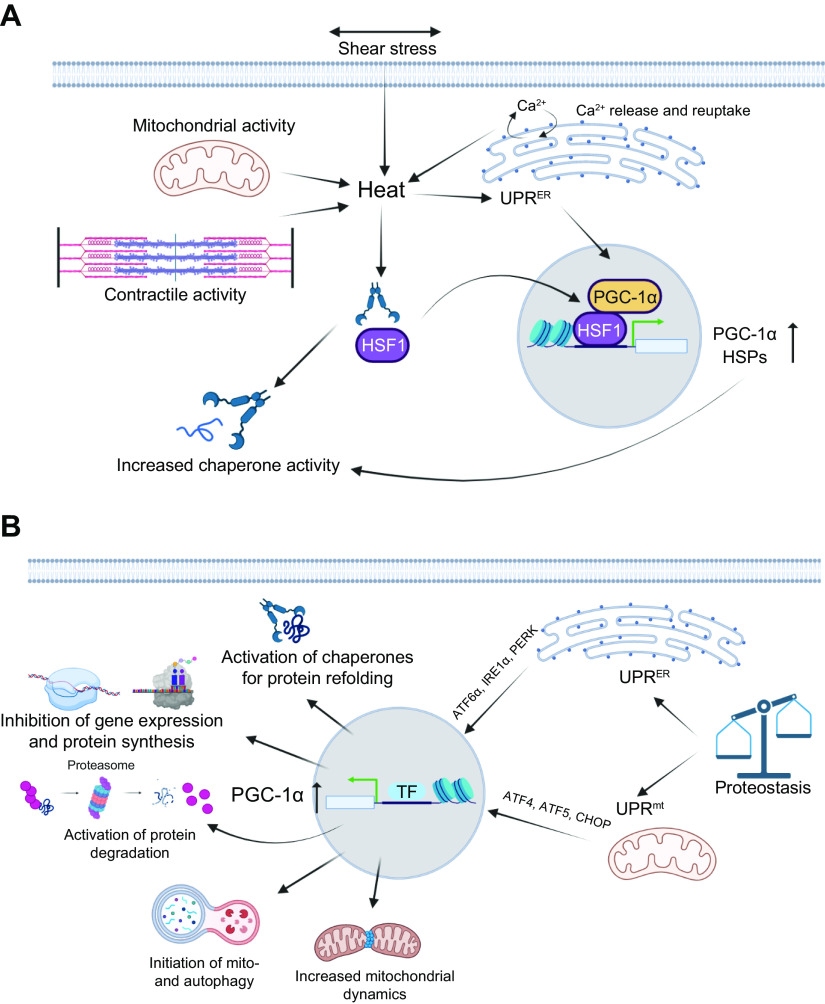
Heat and proteostatic stress. *A*: thermosensing and the heat stress response mitigate misfolding of proteins. Heat is produced by various processes in the contracting muscle fiber, including shear stress, mitochondrial activity, Ca^2+^ release and reuptake, and ATP metabolism in contraction cycling. Heat is sensed in the cell and a broad transcriptional program engaged to increase mitigating measures, e.g., chaperones to reduce thermally induced protein misfolding. *B*: proteostatic stress and the ensuing response pathways reduce protein load, misfolding, and organelle health. Dedicated pathways in the endoplasmic reticulum and mitochondria are engaged by proteostatic dysbalances, e.g., excessive protein accumulation or misfolding. At least in part, these two pathways converge to initiate a transcriptional program aimed at reversing protein misfolding, alleviating proteostatic stress by reducing gene expression and protein synthesis while enhancing protein degradation, and by ensuring organelle functionality. ATF4/5/6α, activating transcription factor 4/5/6α; CHOP, C/EBP homologous protein; HSF1, heat shock factor 1; HSP, heat shock protein; IRE1α, inositol-requiring enzyme 1α; PERK, RNA-dependent protein kinase-like ER eukaryotic translation initiation factor 2α kinase; PGC-1α, peroxisome proliferator-activated receptor γ coactivator 1α; TF, transcription factor; UPR^ER^, endoplasmatic reticulum unfolded protein response; UPR^mt^, mitochondrial unfolded protein response. Image created with BioRender.com, with permission.

Contractile activity is linked to the production of heat, with only a small fraction of chemical energy (∼25% depending on the type of muscular activity performed) being converted into external force production (FIGURE 15*A*) (602). Elevated muscle temperature has several advantages in terms of enzyme kinetics and activity and contributes to vasodilation and increased blood flow, permitting a better supply of oxygen and energy substrates, as well as more efficient removal of by-products. Nevertheless, excess contraction-induced heat production must be dissipated through vasodilation and sweating. Thermal stress can result in misfolding of proteins, impairing cellular function. To mitigate the potentially damaging effects of heat, exercise induces elevated activity and levels of HSPs, most notably HSP72 of the HSP70 family (603, 604). The upstream mechanisms of HSP activation in exercise are poorly understood and likely depend on various metabolic, biochemical, and physical factors as well as training status. Upon heat stress, HSPs are released from binding to heat responsive factor 1 (HSF1) and act as chaperones to refold misfolded proteins and control proteasomal degradation and autophagy (603, 604). HSF1 in turn promotes the transcription of PGC-1α, and together these two proteins induce gene expression of HSPs and PGC-1α in an autoregulatory loop (603, 604). This cross talk with PGC-1α mediates heat shock-boosted oxidative metabolism and mitochondrial function (603, 604). In addition to acting as chaperones and inducing an oxidative phenotype, HSPs exert various other effects in muscle. For example, by inhibiting JNK through direct interaction and upstream signaling pathways, there is a reduction in inflammation and enhanced insulin sensitivity elicited by HSP72 (603, 604). These effects can be potentiated by heat application to reduce muscle soreness, improve muscle function during recovery from damaging exercise, or enhance muscle mass gains in strength training, potentially triggering a hormetic response in which the heat shock response is pre-induced, thereby conferring earlier and/or greater protection (605). Paradoxically, cold-water immersion and other modalities to apply cold stressors after exercise also reduce delayed-onset muscle soreness (DOMS) and inflammation after a single application (606). However, unlike heat exposure, chronic cold therapy diminishes strength training effects by reducing muscle blood flow, attenuating mTORC1 signaling and ribosomal biogenesis, and increasing FOXO3 activity (606). Repeated cold therapy may also interfere with the activation of HSPs by exercise (606). Some current guidelines for the treatment of soft tissue injuries in sports therefore completely omit cold application (607).

#### 4.6.2. Endoplasmic reticulum and mitochondrial unfolded protein response.

HSPs also interact with the regular endoplasmic reticulum unfolded protein response (UPR^ER^), which is activated by accumulation of unfolded or misfolded proteins in the ER (FIGURE 15*B*) (605). During exercise, interaction between PGC-1α and cleaved ATF6α initiates the adaptive UPR^ER^ in muscle (608, 609). ATF6α, inositol-requiring enzyme 1α (IRE1α), and the RNA‐dependent protein kinase‐like ER eukaryotic translation initiation factor 2 alpha kinase (PERK) normally reside in the ER membrane and are engaged upon ER stress, such as in the context of dysregulated proteostasis triggered by resistance exercise bouts (608). Activation drives the release of BiP/glucose‐regulating protein 78 (GRP78), an ER chaperone that then binds to misfolded ER proteins. IRE1α, PERK, and ATF6α subsequently initiate signaling cascades aimed at constraining gene expression and protein synthesis and providing adequate energy availability to normalize proteostasis and alleviate ER stress (608, 609). Exercise-induced ER stress is diminished with repeated exercise bouts, indicating either better control of proteostasis or more efficient resolution of ER protein misfolding after training (608, 609). Like the ER, upon exercise muscle mitochondria also initiate an unfolded protein response (UPR^mt^) to mitigate dysbalanced proteostasis, protein import, and (re)folding, as well as OXPHOS complex assembly by activating mitochondrial chaperones and proteases (610). If overwhelmed, a retrograde signaling is engaged to boost the activities of the transcription factors ATF4, ATF5, and C/EBP homologous protein (CHOP), which in turn regulate the transcription of genes encoding mitochondrial chaperones and proteases, as well as the integrated stress response (610). Mitochondrial and cellular protein quality control are highly coordinated. Initially, cytosolic processes only permit transport-competent folding configurations of proteins to be imported into mitochondria, supported by the ubiquitin-proteasome system that degrades damaged, mislocalized, or improperly imported proteins at the outer membrane (611). Then, mitochondrial chaperones and proteases ensure proper folding and removal of misfolded proteins within mitochondria (611). The next step in escalation of damage results in a remodeling of the mitochondrial network by fission and fusion (402), removal of defective parts of mitochondrial by mitochondrion-derived vesicles or piecemeal mitophagy (612, 613), ultimately culminating in wholesome mitophagy (610, 614). Mitochondrial dynamics, the balance between fission and fusion, are instrumental not only for mitochondrial biogenesis but also for proper morphology of the mitochondrial network (615): fusion leads to larger mitochondria and increased ATP production, whereas fission, promoted in response to severe cellular stress, helps induce the degradation of dysfunctional mitochondria (402, 616). The cytosolic PTEN-induced kinase 1 (PINK1) is normally imported into the matrix of healthy mitochondria and rapidly degraded. Upon deterioration of the membrane potential or the loss of ATP supply, PINK1 accumulates in the outer membrane and phosphorylates the E3 ubiquitin ligase Parkin, which in turn ubiquitinates mitochondrial proteins to target the organelle for mitophagy. An acute bout of exercise might lead to elevated organelle turnover, through both mitophagy as well as general autophagy, by increasing mitochondrial localization of Parkin and activation of the AMPK-ULK1 axis, respectively (610, 614). However, the regulation and functional involvement of mito- and autophagy in acute exercise and chronic training adaptations remain poorly understood (617, 618), especially in elite athletes.

### 4.7. Exhaustion, Fatigue, and Event up to Cessation of Exercise

**FIGURE 16.**
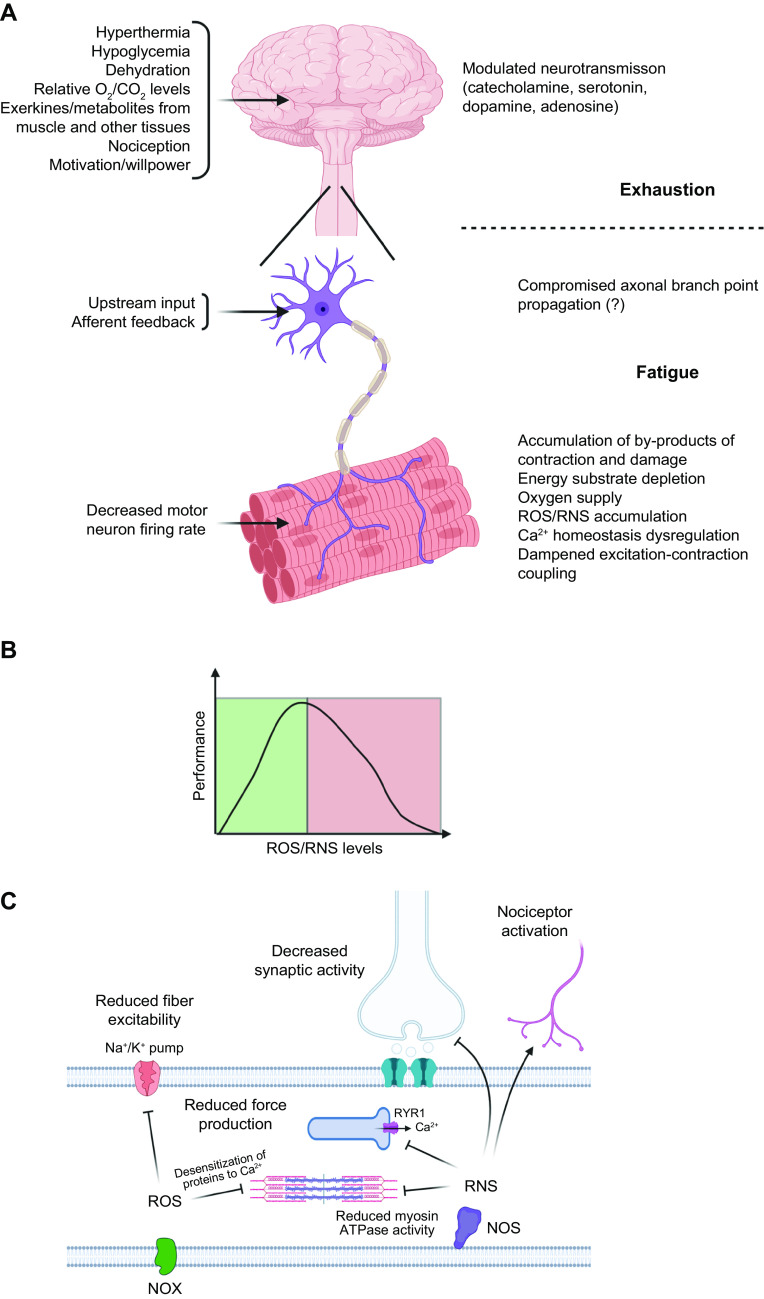
Exhaustion and muscle fatigue. *A*: central and peripheral contributors to exhaustion (volitional) and fatigue (involuntary). Altered neurotransmission in the brain ensures protection of this and other organs from hyperthermia, hypoglycemia, dehydration, shifted O_2_/CO_2_ levels, and further potentially detrimental processes in exercise. To a limited extent, these effects can be overcome by willpower and motivation. Peripheral factors of fatigue involve the motor neuron and muscle fibers. Although impairments in action potential propagation and neuromuscular junction transmission seem minor in healthy individuals, input from upstream brain regions and afferent feedback modulate motor neuron firing rate. Muscle-intrinsic contractility is affected by energy substrate and oxygen availability, accumulation of by-products of contraction and damage, elevation of reactive oxygen (ROS) and nitrogen (RNS) species, a dampening of excitation-contraction coupling including the Na^+^-K^+^ pump, and intramyocellular Ca^2+^ homeostasis. *B*: concentration-dependent, biphasic bell-shaped effect of ROS and RNS on muscle performance. During contractions, ROS and RNS sustain and enhance contractile activity. However, once levels exceed a poorly defined threshold, a different set of processes is engaged that limits performance. *C*: exceeding a certain concentration, ROS and RNS contribute to muscle fatigue by affecting fiber excitability and contractility, synaptic activation, and nociception. A modulation of intramyocellular Ca^2+^ homeostasis thereby plays a central role. Putatively, this “rheostat” helps to avoid overexertion and to minimize muscle tissue damage. NOS, nitric oxide synthase; NOX, NADPH oxidase; RYR1, ryanodine receptor 1. Image created with BioRender.com, with permission.

The completion of an exercise bout is characterized by several processes that contribute to exercise cessation. These can be classified and summarized as exhaustion, a process dependent on an increasing perception of subjective effort that can, to some extent, be influenced and overcome by the individual’s willpower and motivation (619). Physiological pathways resulting in a reduced ability and ultimately an inability of muscles to contract, independent of motivation and willpower, are referred to as fatigue, in which the continuation of contractile activity becomes impossible (619). These definitions already hint at the multifactorial and multiorgan components that contribute to exercise cessation (FIGURE 16*A*). Central factors include catecholamine, serotonin, dopamine, and adenosine signaling in different regions of the brain that evoke a state of mental fatigue (620). Some of these alterations in central neurotransmission might be caused by heat sensing to prevent hyperthermia of the brain, availability of energy substrates (in particular hypoglycemia) to prevent catastrophic contractile failure and inadequate supply for neuronal function, relative oxygen and CO_2_ levels, dehydration, pain/nociception, or metabolite signaling from contracting muscle fibers such as excess ammonia or increased ratio of free tryptophan to branched-chain amino acids in the circulation (619–621). Moreover, elevated cyto- and exerkine profiles in exercise could modulate various processes, including immune cell activity in the brain, and thereby also neuroenergetics and neurotransmission (622). These, and potentially other central mechanisms, most likely help to protect the brain and other organs from detrimental outcomes triggered by overexertion (623). Central fatigue can be overcome by direct peripheral stimulation of muscles. This is not the case for peripheral causes mostly pertaining to the motor unit, ranging from peripheral nervous system signaling to the muscle, ECC, energy supply, and contractility (619). Motor unit fatigue is not well understood, even though motor neurons are classified into fast and slow fatigable pools (329, 624, 625). Failures in axonal propagation, in particular across branch points, or in NMJ transmission seem of little significance in exercise settings and might primarily pertain to pathological situations (619). The observed decrease in motor unit firing patterns and discharge rates could be mediated by afferent feedback and upstream input (619). In some paradigms, fatigue of specific muscles, in particular the respiratory muscles, will limit performance (626, 627). In muscle cells, different processes have been proposed to affect contractility and fatiguability, such as contraction-induced depletion of endogenous fuel stores together with inadequate provisioning of exogenous energy substrates and oxygen supply (619, 628). The accumulation of intra- and extracellular by-products of prolonged contraction or metabolites enriched because of muscle fiber damage could likewise govern fatigue, such as products of energy metabolism, oxidative stress, inflammation, or respiration (CO_2_) (619, 628). In essence, the mechanistic underpinnings of muscle-intrinsic fatigue are not entirely clear. Interestingly, in contrast to the stimulating effect of ROS and RNS on muscle contractility at lower levels, once a critical threshold is reached, dampening effects of accumulated ROS and RNS on performance are observed (FIGURE 16*B*) (629). For example, ROS induces muscle fatigue, mediated by a desensitization of receptors and myofibrillar proteins to Ca^2+^, and a decrease in Na^+^-K^+^ pump activity (FIGURE 16*C*) (534). This concentration-dependent, biphasic bell-shaped effect on muscle performance and fatigue might be a protective mechanism against excessive contractile activity and subsequent tissue damage, with ROS serving as an internal rheostat for the strain exerted on fibers (534). RNS evoke a similar response by initiating processes such as muscle pain and fatigue to mitigate overexertion and overload linked to excessive cellular and tissue damage. For example, NO modulates synaptic transmission by retrogradely affecting presynaptic structures of the NMJ, activates nociceptor complexes containing the NO-sensitive calcitonin gene-related peptide (CGRP) receptor and thereby causes muscle pain, or reduces muscle force production, at least in part by reducing myosin ATPase activity and Ca^2+^ release from the SR by affecting the RYR and the SERCA pumps (536–539). Thus, for both ROS and RNS, modulation of intramyocellular Ca^2+^ homeostasis is central for the regulation of fatigue (629). Of note, the benefits of inhibition of the production of ROS and RNS with pharmacological or nutritional antioxidants to delay fatigue, reduce cellular damage, and shorten the recovery period are superseded by the dampening effects on the procontractile and performance aspects of ROS and RNS during exercise, resulting in an overall reduction in training response/adaptation (534). Chronic elevation of ROS in inactive fibers such as in bedrest leads to pathological outcomes of constitutively stimulated redox signaling in muscle (630). In this context, restoration of ROS levels and redox signaling pathway activity by exercise, pharmacological, or nutritional interventions might confer clinical benefits (630, 631). However, antioxidant treatment with supplements or nutritional components must be carefully tailored to the specific context: oxidative distress and potential antioxidant deficiencies must be confirmed, an evidence-based, personalized treatment strategy designed, and the outcome of the treatment monitored (631). Otherwise, ineffective treatments or adverse effects might result, for example in blunting oxidative eustress in exercising individuals (631).

### 4.8. Repair and Regeneration, Multicellular Cross Talk, and Refueling

#### 4.8.1. Multicellular interactions in muscle repair.

**FIGURE 17.**
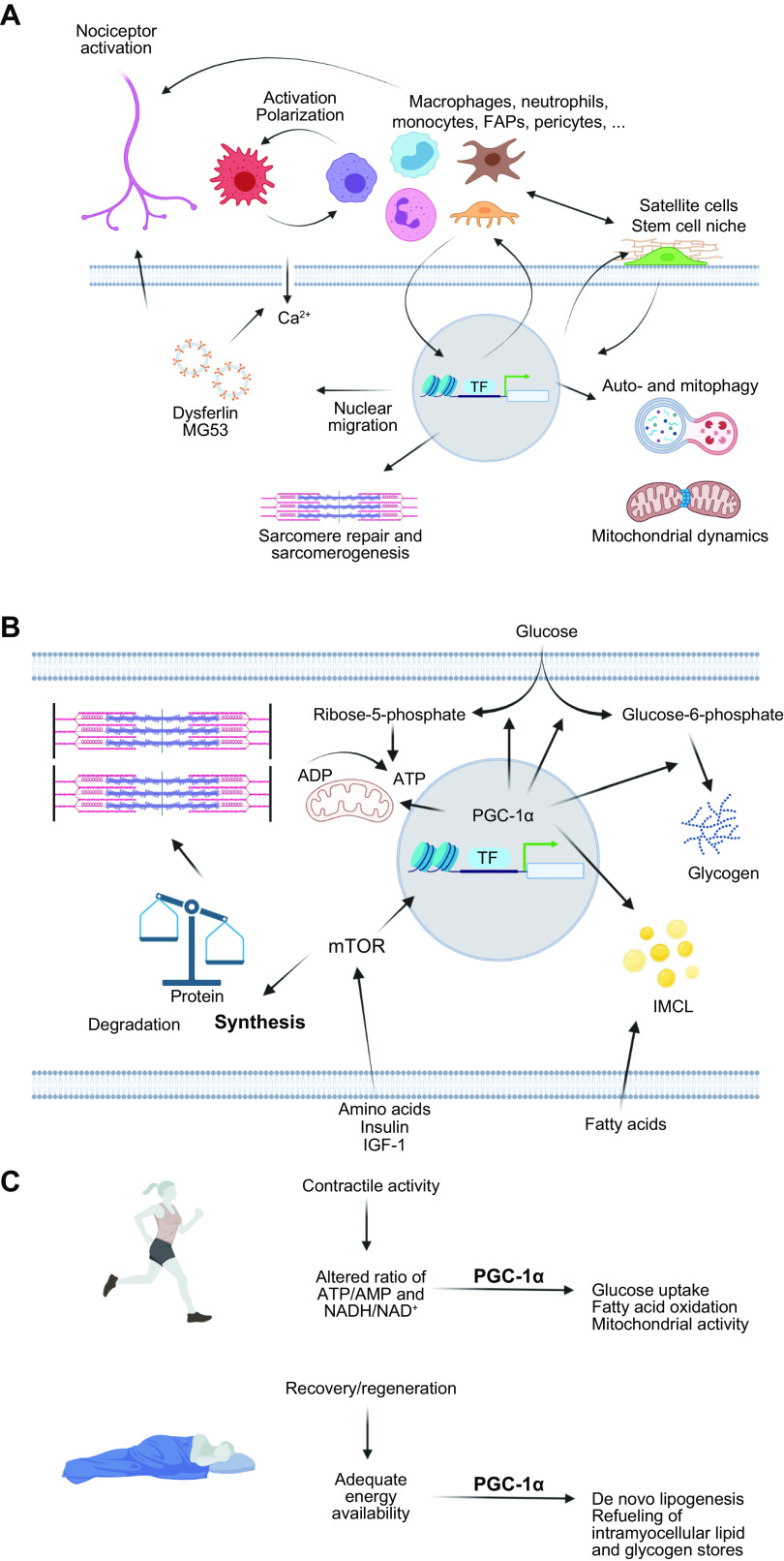
Muscle repair, regeneration, and refueling. *A*: multicellular interactions and intramyocellular processes that control muscle repair. Resident and infiltrating cells of different types are engaged by various signals in a temporally highly orchestrated manner. Thereby, damaged material is removed, muscle fibers repaired or de novo formed, and functional retrieval achieved. Activation of nociceptor signaling should prevent further exertion and damage during repair and regeneration. Moreover, the multicellular processes are complemented by intramyocellular pathways to restore membrane and sarcomere integrity as well as organelle function. *B*: postexercise refueling of glycogen, intramyocellular lipids (IMCL), and protein structures. Depleted intramyocellular energy substrate stores are replenished after exercise depending on a systemic, anabolic context, i.e., the availability of the corresponding substrates and signaling of anabolic hormones. Because of the energetic demand for protein, glycogen, and IMCL synthesis, these processes are coordinated with mitochondrial activity and ATP production. *C*: temporal specification of catabolic and anabolic processes by peroxisome proliferator-activated receptor γ coactivator 1α (PGC-1α). To avoid a futile cycle, the catabolic activity of PGC-1α during contractions must be separated from the anabolic function in regeneration. The mechanistic underpinnings of the transcriptional specification of this coactivator (and other regulators with multipurpose roles) remain unknown. FAP, fibro-adipogenic precursor; MG53, mitsugumin 53; mTOR, mammalian target of rapamycin; TF, transcription factor. Image created with BioRender.com, with permission.

Upon cessation of exercise, a pleiotropic, highly coordinated program of repair, regeneration, and refueling is initiated. Skeletal muscle tissue comprises a complex assortment of different cell types, many of which are still being identified with novel technical approaches such as single-cell RNA sequencing (scRNA-seq) (632–634). As noted above, it is well accepted that the cross talk between different cells is involved in exercise adaptation, such as the release of IL-13 from type 2 innate lymphoid cells (506). To date, however, data describing the processes involved in repair and regeneration of muscle tissue have, to a large extent, come from genetic, pharmacological, and other models of severe physical damage (635–637), with little information derived from physiological in vivo exercise conditions (638). Upon damage, a cascade of events is commenced in which muscle fiber-derived and other signals activate resident immune cells, promote the infiltration of additional immune cell populations, orchestrate the activation, polarization, and termination of immune cell phenotypes, and engage satellite and other myogenic cells to mediate muscle repair and regeneration (FIGURE 17*A*) (635–637). In exercised human skeletal muscle, resident and infiltrating neutrophils, leukocytes, monocytes, and macrophages first promote an inflammatory environment, phagocytose damaged tissue, and clean up cellular debris within hours after an exercise bout (635–637). The infiltration and activation of these immune cells are at least in part orchestrated by a cocktail of cyto- and chemokines that is released from myofibers and other cells including IL-6, C-X-C motif ligand 8 (CXCL8/IL-8), C-C motif chemokine ligand 2/monocyte chemotactic protein-1 (CCL2/MCP1), tumor necrosis factor α (TNF-α), IL-1β, and interferon γ (IFN-γ) (635–637). However, the composition of this cocktail depends on the preceding exercise modality, load, and intensity. The release of a disintegrin, metalloproteinase 8 (ADAM8) and other proteases and the subsequent remodeling of the ECM facilitate immune cell infiltration into muscle tissue (635–637). A shift of macrophage polarization from the pro-inflammatory M1 to the anti-inflammatory M2 type is a hallmark for the second phase, in which tissue is regenerated within hours to days (635–637). The release of IL-10, platelet-derived growth factor (PDGF), transforming growth factor β (TGF-β), IGF-1, angiopoietin, VEGF, follistatin, NO, hepatocyte growth factor (HGF), and other signaling molecules from M2 macrophages, T cells, mast cells, fibro-adipogenic progenitors, and type 2 pericytes boosts fibrotic activity and other pathways to remodel the ECM to accommodate repaired and novel myofibers and stimulate the activation of satellite cells and the formation of capillaries and related processes that are required to reconstitute muscle tissue (635–637, 639, 640). Functional retrieval is completed by the expression of mature myosin heavy chains and subsynaptic NMJ genes to ensure proper contraction and innervation (635–637). This tightly coordinated process is critical for proper regeneration and tissue remodeling. The absence or elongation of the pro-inflammatory response or a reduced anti-inflammatory response impairs muscle regeneration and could result in detrimental events such as fibrosis. Regeneration is typically complete within 4–7 days for most exercise challenges (635–637). In response to muscle-damaging exercise, the infiltration of neutrophils occurs within 24 h, followed by an increase in macrophages after 2–7 days (637, 641). However, the exact time course of muscle regeneration in response to different exercise paradigms is poorly understood, and it is unclear how these processes are engaged in athletes. It remains to be determined whether trained muscles differ from untrained muscles in terms of absolute number of specific cells such as tissue-resident macrophages, lymphocytes, or fibro-adipogenic progenitors or the relative proportion of a certain cell type (i.e., M2 macrophages). The relative amount of myonuclei compared to total nuclear number is lower in soleus compared with the extensor digitorum longus (EDL) (∼40% and 60%, respectively), indicating a higher abundance of nonmuscle mononucleated cells in oxidative muscles that could potentially contribute to better regeneration (642). In line with this observation, a trained muscle might harbor a distinct cellular content and improved regenerative capacity: for example, mice overexpressing muscle PGC-1α exhibit a higher proportion of anti-inflammatory M2 macrophages in muscle tissue and show improved regeneration upon muscle injury (643, 644). Whether the time course of muscle regeneration is accelerated in athletes with a prolonged history of training, thereby hastening recovery, is currently unknown.

#### 4.8.2. Fiber repair and regeneration.

Muscle damage is greater after eccentric/plyometric/lengthening or isometric exercise undertaken with elongated muscle length compared with concentric/miometric/shortening contractions (637). Exercise-induced muscle damage is characterized by a force reduction, systemic increase in myocellular enzymes and proteins such as creatine kinase (CK) and myoglobin, muscle soreness upon palpation, but also swelling and a decrease in range of motion (637). Although some of these symptoms appear immediately after exercise (e.g., loss of muscle force), others such as muscle soreness present 24–48 h later, often being disassociated from CK levels, which peak after 3–4 days (637). Different mechanisms exist to deal with muscle cell damage in an escalating manner (645). For example, membrane lesions are rapidly sensed and repaired, involving specialized features of membrane trafficking components, in particular the action of dysferlin upon influx of Ca^2+^ through gaps in the cell membrane (646, 647), as well as other mediators such as the tripartite motif (TRIM) protein 72 (TRIM72), alternatively called Mitsugumin 53 (MG53), that also serves as a myokine for distal organ repair (648). The restoration of membrane damage is furthermore facilitated by the migration of myonuclei to the site of lesion (649), with this process most likely supported by the exchange of proteins between nuclei (650), and the microtubule-dependent transport of RNAs and ribonuclear proteins within the myofiber (651). Then, mechanical strain leads to non-uniformity and overstretching of sarcomeres, as well as disruption of Z disks, resulting in impaired force production and overload of sarcolemma and T tubules (637). Of note, fast muscle fibers are more susceptible to damage induced by eccentric contractions, which could be due to ultrastructural differences such as the narrow and more fragile Z disks, suggesting that a training-induced shift toward slow muscle fibers might protect the muscle from damage (357). This raises the question of whether muscles of athletes are better protected against recurrent mechanical strains and/or trained muscles have an enhanced repair and regeneration capacity. Mechanosensing, membrane rupture, stretch-activated channels, and dysregulated ECC induce intracellular signaling pathways, such as higher intracellular Ca^2+^, which result in degradation of damaged structures as described above. Neurotrophic factors produced by muscle fibers and satellite cells, in particular activation of the bradykinin receptor B2-nerve growth factor (NGF) and cyclooxygenase 2 (COX-2)-glial cell line-derived neurotrophic factor (GDNF) pathways, stimulate muscle nociceptors and thereby contribute to the pain experienced in DOMS (637, 652). Of note, interference with the inflammatory cascade, even the pro-inflammatory phase, can be detrimental to muscle recovery and adaptive remodeling (637, 653). Massage, thermal therapy (hot or cold), compression, active regeneration, along with various pharmacological and nutritional approaches often have antagonistic effects on DOMS, muscle recovery, and functional remodeling (654). Thus, probably the best practice to reduce future/subsequent muscle damage is to repeat a similar exercise bout, albeit at reduced intensity/loading (637). Accordingly, in resistance-trained individuals, recovery of maximum voluntary isometric torque occurs faster and is accompanied by reduced muscle soreness and lower CK levels compared with untrained individuals (655, 656). These attenuated symptoms of muscle damage, which also resolve after a shorter period, are hallmarks of the “repeated bout effect” (637). A trained muscle therefore has greater protection against contraction-induced damage. However, it is not clear whether this effect is conferred by better resilience against insults, more efficient repair and regeneration, or a combination of these (637). The observation of the repeated bout effect extends to the contralateral, non-trained muscle and suggests a systemic propagation of this signal (637). However, the underlying mechanisms are unknown. Nevertheless, it is clear that improved functional recovery is essential to sustain the high training intensities and volumes of athletes without overreaching/overtraining (657).

#### 4.8.3. Muscle refueling.

Full functional retrieval requires additional processes, including restoration of organelle function, sarcomere repair, and replenishment of substrate stores (FIGURE 17*B*). Optimally, a supercompensation is achieved, as observed for muscle glycogen stores (428). The efficiency of refueling depends on promoting an optimal anabolic environment and is dependent on providing adequate nutritional availability (81). In this setting, energy sensors such as AMPK remain inactivated, and the activity of anabolic regulators such as mTOR are increased by the availability of amino acids, glucose, and fatty acids and stimulation by insulin, IGF-1, and other anabolic hormones (556, 557, 658). The ensuing promotion of protein synthesis is instrumental to support the restoration and de novo formation of sarcomeric and other structures. Intracellular sensors of glucose and fatty acids help to restore glycogen and intramyocellular lipid stores. For example, PGC-1α positively regulates the transcriptional program for lipogenesis, lipid droplet assembly, and perilipins (488, 659). As noted above, the increased intramyocellular lipid level in the muscle of endurance-trained athletes resembles the accumulation of these in muscle from patients with type 2 diabetes (the “athlete’s paradox”). However, although the diabetic patient is insulin resistant, the endurance-trained athlete is insulin sensitive based on the daily turnover and flux of lipid stores (660). PGC-1α also increases muscle glucose uptake while restricting the entry of glucose into glycolysis and boosting glucose-6-phosphate activity in the pentose phosphate pathway and glycogen resynthesis (488). PGC-1α matches these energy-demanding anabolic processes to adequate mitochondrial function and ATP synthesis. Moreover, PGC-1α-mediated pentose phosphate pathway activity produces ribose-5-phosphate, the building block for ATP and the other nucleotides (488). Intriguingly, the anabolic function of PGC-1α in this context is opposite to that during muscle contraction, in which this coactivator strongly stimulates catabolic pathways, including fatty acid β-oxidation (FIGURE 17*C*). Thus, to avoid futile cycles a temporal specification of PGC-1α, including distinct transcription factor binding and DNA element recruitment, is likely, although the molecular characteristics associated with such a specification are unknown.

### 4.9. Circadian Clock

**FIGURE 18.**
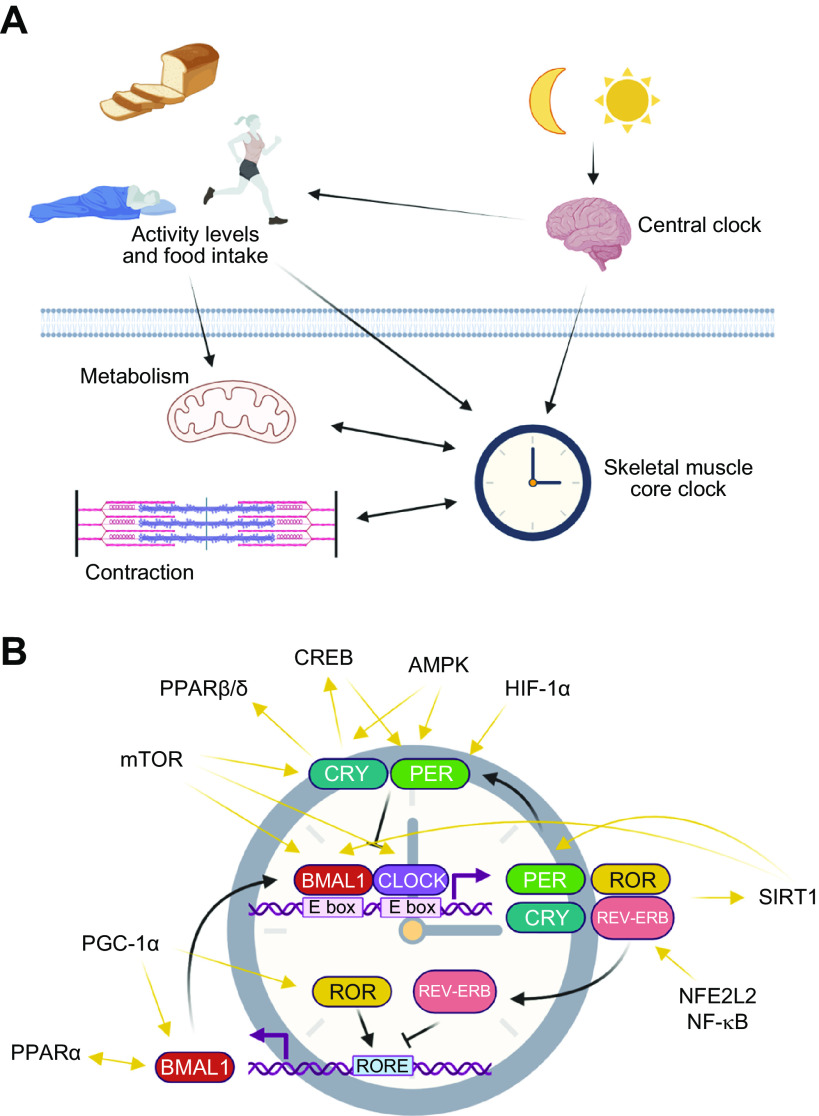
Circadian regulation of skeletal muscle function and plasticity. *A*: circadian regulation of activity and muscle function. Circadian regulation of sleep-wake cycles, feeding, physical activity, or other zeitgebers is sensed and translated by the master clock in the suprachiasmatic nucleus as well as peripheral clocks in almost every cell in the human body. As a consequence, various physiological processes, e.g., muscle cell metabolism or contractile performance, can be affected in a circadian manner. *B*: interactions between core clock genes/proteins with regulators of muscle function and plasticity. Such cross talk pertains to the regulation of gene expression, protein-protein interactions, and/or enzymatic activity. Thereby, physiological processes in muscle might be affected by the core clock. Inversely, muscle fiber metabolism, contractile activity, oxygen availability, redox balance, and other perturbations could modulate the skeletal muscle clock. AMPK, AMP-dependent protein kinase; BMAL1, brain and muscle ARNT-like 1; CLOCK, circadian locomotor output cycles kaput; CREB, cAMP response element binding protein; CRY, cryptochrome circadian regulator; HIF-1α, hypoxia-inducible factor 1α; mTOR, mammalian target of rapamycin; NFE2L2, nuclear factor erythroid-derived 2-like 2; NF-κB, nuclear factor κB; PER, period; PGC-1α, peroxisome proliferator-activated receptor γ coactivator 1α; PPARα/β/δ, peroxisome proliferator-activated receptor α/β/δ; REV-ERB, nuclear receptor subfamily 1 group D member 1/2; ROR, retinoic acid-related orphan receptor; RORE, ROR elements; SIRT1, sirtuin 1. Image created with BioRender.com, with permission.

Almost all physiological processes in humans are under the control of circadian rhythms (661). Locomotion and physical activity belong to the most fundamental aspects of the behavior of higher animals and in an evolutionary context required coordination of activity with the availability of prey and food, the avoidance of predators, along with adequate rest, sleep, and related recovery processes (662). As described in sect. 2, external cues, such as timing of food and physical activity, serve as zeitgebers (time givers) to modulate the circadian clock in cell-autonomous peripheral tissues and thereby adapt many tissues/organs to the prevailing environmental conditions (663). Circadian control of the muscle phenotype is influenced by circadian rhythmicity and executed by a subset of the transcriptome oscillating in this tissue (662), with a reciprocal relationship between the core molecular clock and external stimuli such as the time of exercise training (FIGURE 18*A*). Several regulatory nodes induced by an acute exercise response potentially influence muscle clock oscillations (FIGURE 18*B*) (664–666). For example, activation of AMPK affects the stability of Period2 (PER2) and cryptochrome circadian regulator 1 (CRY1), two components of the core clock (662, 667). CRY1 and CRY2 interact with PPARβ/δ and thereby reduce the oxidative phenotype of muscle cells (662, 667). Circadian Locomotor Output Cycles Kaput (CLOCK) and Brain and Muscle ARNT-Like 1 (BMAL1), two other core clock components, regulate SIRT1 transcription in skeletal muscle. SIRT1 in turn deacetylates PER2 and BMAL1, thus in part counteracting the acetyltransferase activity of CLOCK (667). Bidirectional interactions between the clock, mTOR, and protein synthesis contribute to a link between circadian oscillations and proteostasis (667). HIF-1α induces the transcription of PER1 and PER2, whereas NRF2/NRE2L2 and NF-κB reciprocally control gene expression of nuclear receptor subfamily 1 group D member 1 (NR1D1/REV-ERBα) (667). SIRT1-deacetylated PGC-1α induces the expression of retinoic acid-related orphan receptor α (RORα/NR1F1) and coactivates RORα in the transcriptional regulation of BMAL1 (667). REV-ERBα and RORα are part of an accessory arm of clock control and exert opposite effects in core clock modulation. CREB activity is affected by CRY1 and in turn controls gene expression of PER1 and PER2 (667). Finally, PPARα and BMAL1 exert mutual transcriptional regulation (662). Many of these interactions have not been validated in human muscle skeletal, but there is ample potential for cross talk between factors that are modulated by contractile activity and those that entrain and synchronize the molecular clock (662, 663, 665–667). Accordingly, the gene expression of several components of the molecular clock are affected by exercise, such as BMAL1, PER2, and CRY1 (663). Curiously, other clock components, including CLOCK, PER1, CRY2, and REV-ERBβ/NR1D2, were unaffected by skeletal muscle contractile activity in these studies, which conflicts with a general modulation of clock phase and amplitude by exercise. As such, these data suggest a specific control of a subset of clock genes and not a complete resynchronization. Such a targeted interaction could imply that some of these proteins might have additional functions unrelated to the molecular clock such as the control of cellular metabolism. Indeed, the consequence of the cross talk between exercise factors and the molecular clock for circadian rhythmicity, muscle function, and exercise adaptation is unclear and is likely to be confounded by many factors including meal time, sleep, psychological stress, and other zeitgebers (665). Animal experiments with voluntary wheel running conducted under a skeleton photoperiod suggest that skeletal muscle clock oscillations are robust even in the face of perturbations induced by daytime running and feeding (668). Moreover, studies of the transcriptome, proteome, and phosphoproteome of skeletal muscle of mice undergoing maximal endurance tests at different times of the day indicate a strong association with metabolic parameters such as muscle and liver glycogen concentrations, suggesting more indirect effects of circadian oscillations (668). Thus, future studies need to determine the extent of the influence of the molecular clock on the exercise responses and vice versa, including functional consequences for acute endurance and resistance exercise bouts, chronic training adaptations, and peak performance at competitions.

### 4.10. An Integrative View of the Molecular Mechanisms

**FIGURE 19.**
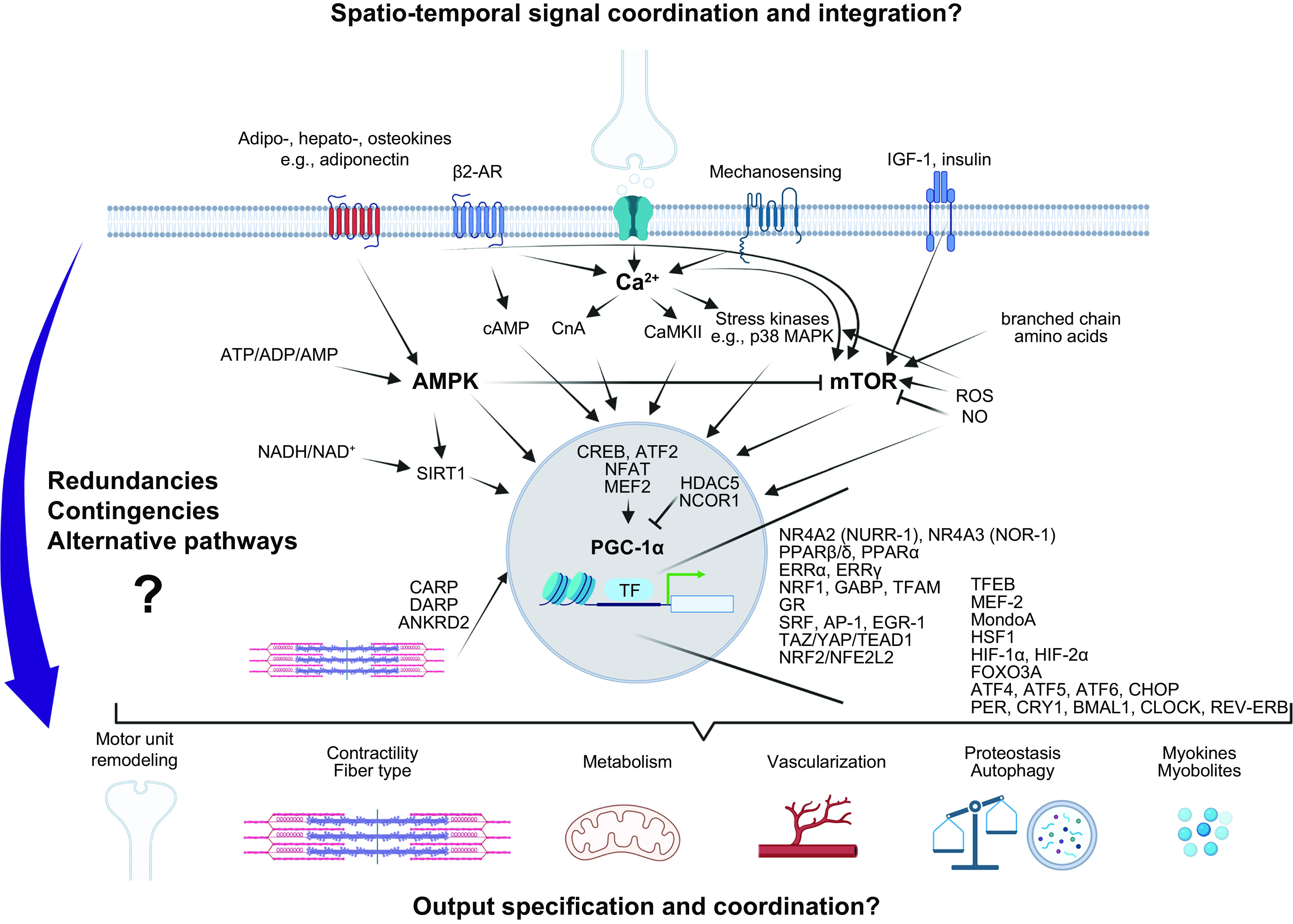
Molecular mechanisms in contracting muscle fibers. A small selection of molecular sensors and mediators as well as a simplified summary of the presumed interactions are depicted. Nota bene: the spatio-temporal integration and coordination of these pathways, functional redundancies (e.g., overlapping or parallel pathways), contingencies (back-up processes with the same function), alternatives (back-up processes leading to similar adaptations using different functions), specification and coordination of downstream effects and adaptations, in particular in chronic settings, as well as many other aspects are still only understood at a very rudimentary level. β-AR, β-adrenoreceptor; CARP, cardiac ankyrin-repeat protein; CnA, calcineurin A; CREB, cAMP-dependent binding protein; DARP, diabetes-related ankyrin-repeat protein; ERRα/γ, estrogen-related receptor α/γ; GR, glucocorticoid receptor; HSF1, heat shock factor 1; NO, nitric oxide; PPARα/β/δ, peroxisome proliferator-activated receptor α/β/δ; ROS, reactive oxygen species; SIRT1, sirtuin 1. See text for other abbreviations. Image created with BioRender.com, with permission.

In this section, we summarized the molecular mechanisms and pathways that are activated in skeletal muscle in response to endurance and/or resistance exercise, with a focus on the putative pathways for which an association between the acute exercise response and chronic training adaptation has been consistently reported. Other reviews have discussed the exercise-induced changes in various pathological conditions such as muscle wasting, cachexia, or sarcopenia (669–672), which may be completely distinct or share some commonalities with those in healthy muscle in well-trained athletes. There are also several pathways and factors that contribute to the pathoetiology of muscle wasting and diseases. Although voluntary and forced muscle inactivity do not necessarily mirror contractile engagement (673), some of these could play a role in exercise training adaptation. For example, the levels of myostatin, which rise in several pathological states (15), are reduced by exercise (674), and the absence of myostatin signaling may be an important contributor to training adaptation. However, this might depend on factors such as baseline control, exercise modality, or the pathological context regarding comorbidities. Related mechanisms such as the repression of activin receptor type II (ActRII), for which myostatin is one of the ligands, by the m(6)A methyltransferase-like-3 (METTL3) could also potentially contribute to muscle hypertrophy after exercise training (675). Readers are referred to recent reviews on muscle atrophy and disease states (560, 676–679). Finally, many studies using gain- and loss-of-function of targeted genes have reported altered muscle metabolism or contractile function, most of which require validation after exercise interventions in both rodent models and humans (485, 486, 680, 681). Many of these factors will undoubtedly add to our current knowledge regarding human exercise-induced muscle plasticity. Notwithstanding these limitations and caveats, we still have a poor understanding of how exercise adaptation is regulated. Most studies focus on individual pathways and factors, often centered on the “usual suspects,” and precisely how the complex and interdependent network of signaling pathways and mechanisms is spatio-temporally coordinated and integrated is unclear (FIGURE 19). A reductionist study of these pathways is difficult because muscle activity and plasticity are under robust control, with numerous redundancies (overlapping or parallel pathways that are engaged in a physiological setting), backups/contingencies (pathways that are engaged if other mechanisms are impaired, controlling the same biological program), and alternatives (processes that are used when others are dysfunctional, leading to different physiological adaptations but resulting in similar outcomes) (18, 41, 682). For example, three of the most characterized factors with putative roles in contraction-induced remodeling (AMPK, mTOR, and PGC-1α) are dispensable for many training-induced adaptations. First, inducible ablation of muscle AMPKα does not affect whole body substrate utilization, muscle glucose uptake, fatty acid, or mitochondrial respiration during exercise (683). Similarly, muscle-specific knockout mice for PGC-1α still increase mitochondrial biogenesis with training, at least in some studies (576). The function of mTORC1, with roles in the early events leading to muscle hypertrophy, is compensated by non-mTOR-dependent pathways during recovery from exercise (581). Integrity of raptor is important for hypertrophy induced by synergist ablation overload but not the related boost in the rate of protein synthesis (684). Genetic models with inducible muscle-specific inhibition by ablation of raptor and sustained activation of mTORC1 by disruption of TSC1 have little effect on preserving muscle mass (524) or facilitating hypertrophy (685), respectively. It is conceivable that such results are caused by flawed or imperfect experimental model systems, with many studies relying on constitutive gain- and loss-of-function. Moreover, targeting strategies might be imprecise, such as the use of raptor and rictor to genetically ablate mTORC1 and mTORC2, respectively, leveraging TSC1/2 to activate mTORC1 activity, or using rapamycin as a pharmacological inhibitor of mTORC1. Even though raptor and rictor are unequivocal members of mTORC1 and mTORC2, many other potential interactions of these proteins beyond their direct function in forming the mTOR complexes have been proposed (686) or demonstrated (687). Similarly, TSC1/2 and the downstream effector RHEB clearly result in activation of mTOR but potentially also the modulation of the function of other proteins (688). Furthermore, physiological mTOR activity is regulated transiently and in a pulsatile manner, quite different from the sustained, long-term loss- and gain-of-function in the animal models. Finally, rapamycin is a potent inhibitor of mTORC1 activity but at different dosages and administration durations serves also as a modulator of the activity of other protein complexes, including mTORC2 and signal transducer and activator of transcription 3 (STAT3) (689). Notably, STAT3, like mTORC1, exerts effects on muscle mass (690). However, these seemingly paradoxical findings in relation to PGC-1α, AMPK, and mTOR should not necessarily be interpreted as a sign of their dispensability or insignificance in the physiological exercise response but rather as proof of the resilience of whole body systems to adapt even under adverse conditions. For example, although muscle-specific PGC-1α knockout animals can partially adapt to exercise training, this adaptation differs from the physiological training response seen in wild-type animals and occurs despite a lack of vascularization or metabolic adaptations in lactate and ketone body metabolism, thus presumably relying on alternative processes that provide similar benefits (575–577). How such compensation is achieved is unclear, but it is conceivable that the transcription factor binding partners still regulate the corresponding target genes even in the absence of this and other coregulators, albeit at lower levels or altered target specificity (474, 486). PGC-1α can regulate target genes with different binding partners, such as those involved in the hypoxic response by coactivating AP-1 or ERRα, implying functional redundancies in the transcriptional network engaged in exercise adaptation at different levels (474). Future studies should aim at obtaining an unbiased, holistic picture of the molecular mechanisms that control muscle plasticity in response to endurance and resistance training (18, 40). Such insights might also be important to better understand training interference effects and help in the design of concurrent training programs for athletes involved in multisport events (i.e., the triathlon) or who require traits of both endurance and power for successful performance. Finally, our knowledge about the mechanisms that control chronic adaptations to endurance- or resistance-based training is sparse compared with that describing the responses to a single bout of exercise. Many of the adaptations in elite athletes with a prolonged history of training are not reflected in the transcriptional changes that occur after one acute exercise bout, such as the change in expression of different myosin heavy chains (9). Therefore, it is unlikely that a trained muscle only reflects the additive or sustained changes that are induced in individual acute exercise bouts (9). Epigenetic changes might be involved in priming and/or altering the gene expression profile of trained versus sedentary and of acute exercise-induced changes in the trained versus the naive state (41, 585, 691). Precisely how the temporal sequence of individual exercise bouts over time results in training adaptation remains a fertile area for future research. Together, sects. 2–4 have described training practices and the physiological and molecular pathways involved in the acute exercise response; sect. 5 integrates these aspects to discuss interindividual differences and the specific aspects that underpin elite athlete performance.

## 5. CAN WE ALL BECOME GOLD MEDALISTS?

### 5.1. Individual Responses to Training

Elite sporting performance is the result of the interaction between genetic and training-related factors (692, 693), and although several genes or gene clusters have moderate associations with performance or performance-related phenotypes (694–696), the genomic signatures associated with elite athletic performance across a wide range of events/sports have yet to be identified. Notwithstanding the lack of direct links between genetic variants and elite performance, the notion that there exist interindividual responses to exercise training and that innate factors may explain a large part of the training-induced variance in maximal aerobic capacity in previously untrained persons can be traced back to the classic HERITAGE studies conducted in the 1980s by Bouchard and colleagues (697). In these and other recent investigations, the most common reported primary outcome measure in response to endurance-based training programs was an individual’s $\dot{\text{V}}{\text{O}}_{2\max}$ attained during an incremental exercise test to exhaustion, either treadmill walking/running or ergometer cycling, usually lasting 10–15 min. After 2–3 mo of endurance training (3 or 4 sessions per week), $\dot{\text{V}}{\text{O}}_{2\max}$ is typically increased by 10–15% at the group level, but the magnitude of improvement can be as little as 1–2% and as great as 35% (698). A key observation from these early human training studies was that up to 20% of subjects demonstrated little change in $\dot{\text{V}}{\text{O}}_{2\mathrm{peak}}$ in response to a standardized training protocol and were considered “exercise resistant” (699). However, individuals who demonstrate a low training response to one outcome measure (e.g., $\dot{\text{V}}{\text{O}}_{2\max}$) do not always display the same response in other parameters. This makes the concept of responders versus non-responders open to different interpretations. Indeed, exercise training induces a multitude of health- and performance-related benefits (9, 700), some of which may even have a non-physiological basis (701).

Recently, the notion that there exist exercise-resistant subjects or non-responders has been challenged on the grounds that in those studies in which individuals exhibit no meaningful change in a specified outcome variable, the training impulse has been inadequate in terms of either volume or intensity overload (702). To test this hypothesis, Montero and Lundby (702) recruited 78 healthy young male volunteers and first subjected them to 6 wk of supervised training. Individuals trained in five groups differing in the number of exercise sessions per week: they performed 60 min of cycle ergometer exercise either one, two, three, four, or five times a week, corresponding to 60, 120, 180, 240, and 300 min total training time. After completing this first phase of training, the group mean maximal work rate sustained during an incremental cycle test (W_max_) was increased in all groups apart from the group who undertook just a single training session per week. In groups one, two, three, four, and five, 69%, 40%, 29%, 0%, and 0% of individuals, respectively, were non-responders [i.e., did not have a significant increase in the maximal work rate (W_max_) attained during an incremental test to exhaustion]. Subsequently, individuals classified as non-responders to the first phase of training performed a second 6-wk block of training in which two additional 60-min sessions per week were included, independent of the number of sessions completed during the first 6 wk. Following this protocol of volume overload, the lack of training-induced increase in W_max_ was universally abolished. Although further research needs to be undertaken to determine whether such findings can be extrapolated to other populations, these results fundamentally challenge the notion of non-responders and suggest that if a training stimulus is of sufficient volume and/or intensity and follows the principles of progressive overload and specificity, then those individuals thought to be exercise resistant or low responders can indeed become “responders” (702). In this context, Booth and Laye (703) believe that the term non-responder should be replaced by individuals who demonstrate “low sensitivity” to a training stimulus and that such individuals merely require increased training volumes and/or intensity to drive favorable responses. At the population level, focusing on only selected measures of training response and labeling an individual as a non-responder is somewhat of a reductionist approach to exercise. Indeed, if we accept that exercise is a “polypill” (46, 704) exerting a plethora of positive benefits (700), then by focusing on a small number of measures of response we ignore the fact that exercise works through so many different pathways and mechanisms that the chances of an individual exhibiting no single biological benefit is highly unlikely (701, 705).

Regarding elite athletic performance, the issue of low responders or non-responders becomes somewhat irrelevant: to become an elite performer, one must be initially well endowed in the traits that have critical roles in the athlete’s event, and one should also be a high responder to exercise training (83). Furthermore, without a rich genetic endowment, world-class athletic performance is unattainable (83). In this regard, there are substantial differences in performance-related traits measured in the sedentary state (i.e., before any training intervention). Individuals who have high levels of a trait before exposure to exercise training are greatly predisposed to experiencing early success, which also might have a big impact on motivation and subsequent training adherence. This is not the case in non-elite sedentary populations in which no correlation is observed between the baseline level of fitness (i.e., $\dot{\text{V}}{\text{O}}_{2\max}$) and the response to an exercise training dose (699), suggesting a unique biology underlying trainability. In support of this contention, Rønnestad et al. (292) report the case of an individual with a $\dot{\text{V}}{\text{O}}_{2\max}$ of >70 mL/kg/min in the untrained state that increased by 30% after 3 yr of specialized training to >95 mL/kg/min and coincided with a world championship title. Animal studies of selected breeding for aerobic running capacity also reveal high and low responders to standardized exercise training. To determine the inherited components of acquired running capacity, a model of two-way artificial selection for animals that were either low or high responders to exercise training was developed (706). These two animal lines were tested for maximal treadmill running distance before and then after 8 wk of standardized treadmill run training protocol (i.e., the same absolute training load). After 15 generations of selection, and 8 wk of training, the high-response rats improved an average of 223 m in run distance, whereas exercise capacity declined by 65 m in the low-response animals. Taken together, a continuum of responses to standardized exercise training protocols exists (698), yet low sensitivity to adapt may be mitigated by revision of exercise prescription including training frequency, duration, and intensity (701, 702). Although an understanding of how genetic factors contribute to interindividual training responses may improve personalization of training prescription, at present genomic predictors for response trainability are lacking (707).

### 5.2. Genetic Predisposition to Becoming an Elite Athlete

An indication of genetic predisposition toward athletic prowess could be inferred from a variety of observations. For example, people from the western parts of Africa (including Ghana and Nigeria) as well as the descendants of the slaves that were transported from these regions to the “New World” (the West Indies or the United States) are excellent sprinters. In contrast, athletes from Eastern Africa, such as Kenya and Ethiopia, are famous for their extraordinary long-distance running feats. In fact, ∼90% of elite marathon runners worldwide are of Ethiopian or Kenyan descent (708). However, it is unclear whether different genetic, and in extension anthropometric (709) or rather environmental, factors and training practice (118) explain this phenomenon. For example, the higher altitude plateaus of the Eastern African countries might facilitate endurance training adaptations in contrast to the mostly sea-level landscape of Western Africa. Despite these alternative explanations, it is estimated that ∼65% of athletic ability can be explained by genetic factors (710). Moreover, there are data indicating that maximal endurance capacity as well as trainability is inherited (699, 711) and that the genetic makeup accounts for a substantial contribution to performance levels (712). However, despite evidence that genetic components are strongly related to the phenotypic traits of elite athletes, knowledge of the specific genes underpinning this predisposition is limited. Rare examples for extreme genetic variants underline the genetic contribution to athlete status. One example is the mutation in the EPO receptor gene that resulted in a more active truncated protein in the Finnish cross-country skier Eero Mäntyranta (713). His Hb levels were at least 200 g/L, which is substantially higher compared with other endurance athletes or nonathletes (∼150 g/L) and could thereby have contributed to the three Olympic gold medals and two World Championships he won over his career (266, 713). In broader populations, the two polymorphisms that are most described and linked to athletic performance are located within the ACE and ACTN3 genes that encode for the angiotensin I-converting enzyme and α-actinin-3, respectively (714). In fact, the first polymorphism that was associated with athlete status was ACE I/D (715). ACE is part of the renin-angiotensin system and is involved in regulating blood pressure by converting angiotensin I to the active vasoconstrictor angiotensin II. ACE activity in serum is lower in the presence of the insertion (I) allele containing 287 base pairs within intron 16 (716). The I allele is associated with successful endurance capacity, whereas the deletion (D) allele is associated with prowess in strength/power events (716). However, inconsistencies exist within this classification: in elite endurance runners, an association with the D allele or the I allele as well as a lack of any association have been reported (708, 714). In comparison, data on ACTN3 polymorphisms are more robust. ACTN3 is an actin-binding protein that is exclusively expressed in type II fibers and located at the Z disk, suggesting a role in high-velocity force contractions (717). The single-nucleotide polymorphism (SNP) in the ACTN3 gene results in a premature stop codon (X) instead of the arginine (R) at position 577, and the XX genotype is deficient in expressing the ACTN3 protein (718). The 577R allele has been associated with elite sprint/power athletes and explosive performances, RR being superior to RX and XX genotypes (719, 720). In contrast, the XX variant is more frequently observed in elite endurance athletes compared with non-athletes and is extremely rare in elite power athletes (719). In line with these observations, the absence of ACTN3 results in an increased endurance performance and higher oxidative phenotype in the muscle of knockout mice (721).

During the last two decades, at least 155 polymorphisms related to elite endurance (93 polymorphisms) or power athletes (62 polymorphisms) have been identified (722). Endurance markers that have been replicated in at least three independent studies include ACE I, ACTN3 577X, HFE (homeostatic iron regulator), PPARA, and PPARGC1A, and power markers include ACE D, ACTN3 577R, AMPD1 (adenosine monophosphate deaminase 1), HIF1A, MTHFR (methylenetetrahydrofolate reductase), NOS3, and PPARG (722). Of all 93 polymorphisms associated with elite endurance athletes, the 3 located within the ADRB2 (adrenoceptor β2), ADRB3, or PPARGC1A genes have also been shown to be associated with baseline $\dot{\text{V}}{\text{O}}_{2\max}$ of a non-athlete population (723) and the 5 variants of the ACE, AMPD1, CKM, HIF1A, and PPARD genes with $\dot{\text{V}}{\text{O}}_{2\max}$ trainability (724). However, even though several genetic variants have been identified in genomewide association studies (GWASs) of elite athlete status, there is no subset of genes that allows the identification of elite athletes (696). Often, studies are underpowered, which is not surprising regarding the limited number of elite athletes and accessibility of biological samples, and therefore many results cannot be replicated in different cohorts (696). Based on a meta-analysis including 1,520 endurance athletes and 2,760 non-athletes control subjects, a polymorphism in the GALNTL6 gene, encoding for polypeptide *N*-acetylgalactosaminyltransferase-like 6, may be a significant marker for athletic performance (696). In endurance athletes, the C allele is overrepresented compared with non-athletes, whereas the T allele of GALNTL6 is more frequently observed in elite power athletes compared with endurance athletes or non-athletes and is associated with higher peak power in active men (696, 725). Another recent meta-analysis in elite endurance athletes identified polymorphisms in the MYBPC3 (myosin-binding protein C3) and NR1H3 (nuclear receptor subfamily 1 group H member 3/LXRα) genes that were also correlated with $\dot{\text{V}}{\text{O}}_{2\max}$ (726). Specific SNPs in the HFE, NFIA-AS2 (nuclear factor I A antisense RNA 2), and TSHR (thyroid stimulating hormone receptor) genes that are more frequently observed in elite endurance athletes compared with controls have also been associated with high $\dot{\text{V}}{\text{O}}_{2\max}$ among athletes (727–729). Additionally, athletes with homozygous C alleles of NFIA-AS2, encoding a long noncoding RNA, have improved hematologic parameters such as higher Hb levels, since NFIA-AS2 may be involved in the regulation of the transcription factor NFIA and erythropoiesis (727, 728). However, even if a combination of GWAS and selected physiological measures in elite athletes may help identify SNPs for various genes, data including such measures are limited and so far these candidates have not been replicated (722). In the future, it might be possible to link polymorphisms to the responsiveness of individuals (i.e., low vs. high responders) to standardized training interventions, as a set of 21 SNPs was able to predict almost 50% of the variation observed in the $\dot{\text{V}}{\text{O}}_{2\max}$ training response (698). However, as noted, replication of these data remains difficult, and not all SNPs seem to affect trainability across all population groups (698). Importantly, the identified polymorphisms are based on associations, and for the large majority of these the functional relevance and mechanistic aspects of the gene variants in muscle biology are unknown. Therefore, identification of SNPs with larger effect sizes, replication of the identified SNPs in independent cohorts, as well as studies including a larger sample size and possibly additional physiological measures to discriminate individuals (i.e., $\dot{\text{V}}{\text{O}}_{2\max}$ and performance outcomes) are necessary to gain knowledge about genetic factors that underpin elite athlete performance. These studies will have to be combined with mechanistic investigations of the functional effect of gene variants and SNPs to understand how differences are brought about.

Gain- and loss-of-function studies in mouse models have identified 31 genes associated with endurance performance (681). For eight of these, genetic variants have been reported to be associated with elite endurance athletes (681, 722). The genes associated with an elite endurance athlete status and enhanced endurance performance in a gain-of-function mouse model include PPARD, PPARGC1A, PPARGC1B, and PPP3CA (protein phosphatase 3 catalytic subunit α), and those correlated with endurance performance in a loss-of-function mouse model include ACTN3, ADRB2, BDKRB2 (bradykinin receptor B2), and HIF1A (681, 722). Although 47 genes were identified in mouse models that induce hypertrophy in a gain- or loss-of-function model, no corresponding human polymorphisms were described for most of the genes that were associated with a strength/power muscle phenotype (680). However, gene variants in IGF1 and ADRB2 have been found in the phenotype of power athletes and are 2 of the 47 genes that induce hypertrophy in a gain-of-function model (722).

Collectively, most of the identified polymorphisms differ between studies and only explain a very small fraction of the interindividual differences in endurance and strength. As a consequence, the current knowledge is inadequate for talent identification or prediction of training response (68). Besides the question of whether genetic talent identification will ever be feasible in the future, ethical and practical issues also need to be considered (718, 730). “Genetic” prediction of athletic prowess and specialization disregards personal preferences and choices, with potential detrimental consequences on enjoyment, motivation, and ultimately adherence. Talent identification and premature specialization might also preclude the multidisciplinary practice in youths that predicts world-class performance (731). Moreover, sensitive genetic information has potential for misuse and unexpected outcomes, and could have psychological consequences that could even extend to other family members. Additionally, the reported associations are observed at the population level and hence have a very low predictive value at the individual level (732). In fact, there are frequently individuals with a seemingly less favorable genotype who achieve elite athlete status (718). Moreover, monozygotic twin studies revealed a strong impact of discordant leisure-time physical activity on performance, fitness, health, and well-being, to a large extent disconnected from the genetic endowment (733). In summary, the current scientific evidence supporting the contributions of specific genetic variants to elite athletic performance phenotypes is weak (734). This is partly because complex traits are modulated not by several genes with large effect sizes but instead by polygenic systems defined by hundreds or thousands of loci, characterized by alleles with small effect sizes, plus less frequent alleles (83). In contrast to the commercial for-profit genetic testing to predict training selection and response, non-genetic testing, such as assessment of physical performance, might be more useful for athlete stratification (68, 83, 692, 693, 735). Importantly, instead of talent identification, genetic information could also be used for the screening of polymorphisms that are associated with injury risk among athletes (i.e., stress fractures or tendinopathies) to reduce injury by individualized preventative measures (718), with much fewer ethical considerations attached.

### 5.3. The Aging Athlete and Athletic Performance: Slowing Down with Speed

During the past century, there has been a steady increase in life expectancy among most countries in the Western world. However, such enhanced longevity (life span) has not always been accompanied by a proportional elevation in healthy life expectancy, so-called “healthy aging” or “healthspan” (736). Thus, an objective of medical and aging research is not necessarily to prolong life span per se but instead to increase the healthspan and compress morbidity later in life (737). At the population level, aging is strongly associated with a rise in sedentary behavior and concomitant declines in physical and mental capacity. An examination of the performance profiles of individuals who continue to train and compete throughout their entire life provides insights into the extremes of human function and the upper limits of physiology during the human aging process (738, 739). The birth of the “masters” athlete movement (those individuals >35–40 yr of age, depending on the sport) can be traced back to the late 1960s and early 1970s. At this time, there was a massive increase in the number of people who started exercising, either for health and pleasure or to pursue competitive endeavors. In the United States, this escalation in structured physical activity initially centered on distance running and was inspired by a few select individuals such as Roberta Gibb and Kathrine Switzer, who were the first female finishers in the Boston marathon in 1966 and 1967, respectively, and Frank Shorter, who won the gold medal in the men’s Olympic marathon in 1972 and silver in the 1976 Olympics. Throughout the next two decades, there was an explosion in the number of marathon races held in capital cities throughout the world, with applications to run in these events far exceeding the number of available starting places. The mid-1970s also saw the birth of the triathlon, which consisted of an amalgamation of three separate sports: swimming, cycling, and running. From humble beginnings (15 men started and 12 finished the inaugural “Ironman” triathlon in Hawaii in 1978), the Hawaii Ironman is generally considered one of the most difficult 1-day sporting events in the world, and today Ironman races attract almost half a million entries worldwide each year. These competitive, mass-participation inner-city marathons and triathlons were the motivation for a generation of women and men to start training for specific competitions/races and laid the foundation for a generation of athletes who are now in their eighth or ninth decade of life and have participated in formal exercise training throughout their life span (740). As such, we now have both cross-sectional and longitudinal data on a cohort of masters athletes who have maintained rigorous training schedules over many decades and have better health outcomes than their age-matched non-athletic counterparts. Such well-trained individuals represent the optimum phenotype for examining the effects of aging on performance and vice versa, as these individuals are most likely minimally affected by the negative effects of the age-related decrease in voluntary physical inactivity and changes thus largely driven by an inherent aging process (739). Although cross-sectional data on masters athletes are easier to obtain than longitudinal data, the former can only provide the age-related performance decline for a population, whereas longitudinal data show individual trajectories. A detailed analysis of the age-related declines in performance across multiple sporting events is beyond the scope of this review, and the reader is referred to previous work in this area (741–746). Here, we provide a general overview of the effects of aging on overall physical performance declines and discuss some of the mechanisms that underpin this decay in performance capacity.

**FIGURE 20.**
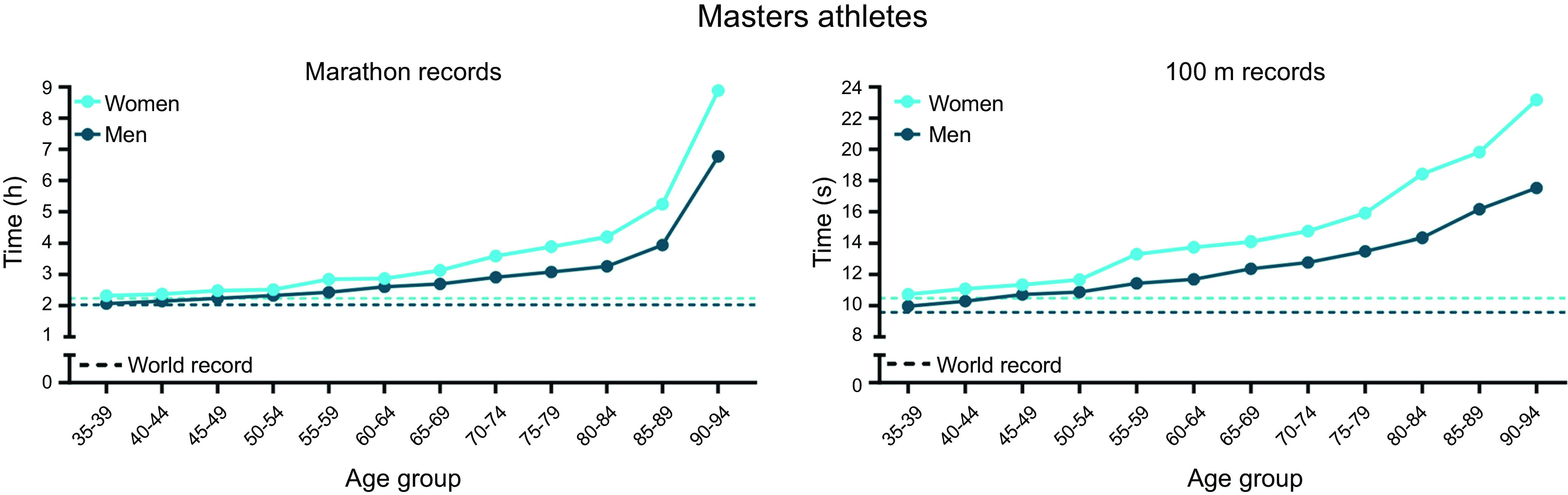
Age-related decline in records of sprint and endurance events. Records for marathons (https://world-masters-athletics.com/championships/non-stadia/) and 100 m sprints (747) of masters athletes represent the age-induced reduction in tissue and organ function that despite high training loads leads to a decrease in performance. However, they also highlight the potential of the human body to achieve incredible performances at advanced age with adequate training.

Plotting age-group world records for males and females across various sporting events provides insights into the rates of decline in performance capacity across the healthspan (FIGURE 20). Such cross-sectional data offer the “best-case scenario” for each age band but do not provide any information on the individual athletes’ decay curves. Several observations are noteworthy. First, the rate of performance decline does not appear to differ between or within sports (e.g., the various track and field events and multiple swimming strokes and distances) (745). Second, there are no major sex-related differences in the deterioration in performance for most sports (746), and although there is a greater absolute drop-off in performance for females compared to males, the relative decline is similar. Performance declines for most of the running, swimming, and cycling events, independent of distance, are not linear but curvilinear (741). However, at ∼70 yr of age, there is an accelerated increase in performance decrements for almost all sporting performances. For example, world records show rapid declines after age 70 in swimming, long-distance running, and sprint performance (744). Although it is unlikely that those athletes still training and competing after the age of 70 can maintain the same absolute training volumes and intensities, there is no reason to suspect that the relative training intensity has diminished. It is worth noting that only 5% of athletes competing at age 80 yr are still competing at age 90 yr. Therefore, world-best performances in these latter years may merely reflect a lower number of participants rather than any underlying physiological factor responsible for the drop in performance capacity. Nevertheless, the performance decline in athletes aged ≥80 yr is threefold greater compared with athletes aged 30–69 yr (1.62% vs. 0.46% per year) and accelerates around 67 yr, especially for sprint/power disciplines (742, 743).

The factors that constrain performance with aging are event-specific, meaning that for each sport/event there may typically be one or more physiological/mechanical systems that limit exercise capacity. For example, the decline in maximal running velocity (independent of distance) is likely to be underpinned by deteriorations in several properties in skeletal muscle that include a decrease in the maximum strength or power output, a slower rate of force development and transmission, and a reduction in elastic energy storage and recovery of tendons (748). During the normal aging process, there is a progressive loss of muscle mass, mainly in the lower body, which increases after the age of ∼45 yr, with the absolute decline being faster in men than in women (749). Even in masters athletes who have undertaken a lifetime of training, a reduction in size and function of muscle is observed, which is most pronounced in the fast fibers (750). This selective loss of fast-twitch fibers explains, in part, the greater magnitude of performance decline in sprint and explosive events compared with endurance-based activities. Cross-sectional data from 37- to 90-yr-old masters athletes indicate that peak anaerobic power declines by 7–14% per decade, with this reduction being similar between male and female athletes (751). Despite the selective loss of fast muscle fibers, Cristea et al. (752) have shown that a 20-wk program that combined sprint training with heavy and explosive strength exercises specifically targeting fast-twitch muscle fibers improved maximal, explosive, and sport-specific force production in elite masters sprinters (aged 52–78 yr). Furthermore, at a single-fiber level, it seems that power output per unit size (i.e., muscle quality) is not reduced in muscles of aging athletes (753), suggesting that the loss in power output can mainly be ascribed to the loss of fast muscle fibers rather than muscle quality. In contrast to sprint-based activities, men and women with a prolonged history (5 days/wk for 7 h/wk over the past 52 yr) of endurance-based training have capillarization and aerobic enzyme activity similar to younger (25 yr of age) exercisers, which in turn is 20–90% greater than the parameters determined in elderly age-matched non-exercisers. Although whole muscle responses offer unique insights into age-related muscle deterioration, they fail to provide a better understanding regarding the potential mechanisms underlying this phenomenon.

With endurance-based sports, there is a decrease in $\dot{\text{V}}{\text{O}}_{2\max}$ of ∼1% per year after the age of 35 yr (754). This is due to a combination of factors including, but not limited to, an increase in ventricular stiffness that contributes to worsening left ventricular diastolic function reflected by reductions in early inflow velocity, ratio of early to late inflow velocity, and early diastolic tissue velocity and increases in the isovolumic relaxation time and the time constant of isovolumic pressure decay (755). Although lifelong exercisers have a greater stroke volume and consequently a superior functional capacity and cardiovascular reserve than their sedentary peers, there may be some adverse consequences of a lifetime of rigorous endurance-based training. Several studies have reported increased risk of atrial fibrillation, atherosclerotic plaques, and aortic dilation in masters athletes compared with age-matched untrained individuals (756–758). A J-shaped association between exercise volume and all-cause mortality, cardiovascular disease, and total cancer, as opposed to the L-shaped association with diabetes, would imply a negative risk profile at very low and very high volumes of activities (759). These findings, however, contrast with results of other studies in different athlete populations, in which a J-shaped association was not observed (760, 761). In fact, many studies and meta-analyses reveal reduced mortality in former athletes (761–763), even in those competing at the highest level such as United States, French, or Polish Olympians or French participants of the Tour de France (764–767). Often, assessment of potential effects of genetic components and the benefits of lifelong training in elite athletes is confounded by healthier lifestyle habits such as being more active or a non-smoker (768, 769).

### 5.4. Looking Ahead: Personalized Training Using Wearables

The application of wearable appliances/sensors to sport is a relatively new phenomenon. In 1978, a Finnish company pioneered the first wearable heart rate monitor, subsequently introducing a monitor with an integrated computer interface, which gave athletes the ability to view and analyze their training data on a computer for the first time. In 2009, a professional European soccer club used wearable devices for measuring player workload during games. That device was among the first to allow coaches real-time monitoring of each player’s biometrics for signs of exhaustion or injury while on the field of play. The generation of real-time data during competition situations is important: athletes have long been able to monitor their physiological status under controlled laboratory conditions, but competition demands including prevailing environmental factors (heat and humidity, wind speed, altitude, other athletes/players, crowds) impose a different set of stressors that cannot be mimicked in the laboratory (770). Over the last 20 years, the growing interdisciplinary merging of technologies has led to milestones in the field of advanced sportswear systems. Such systems are designed to assist athletes to reach their desired fitness and performance goals by helping to create an optimal micro- and macroenvironment and/or physiological state for comfort, to facilitate best performances by providing real-time information on the environment and state athlete status (770). Of note, most sensors for monitoring athletic training loads and sports performance have been driven by technological advances in other disciplines, mainly clinical medicine (e.g., continuous glucose monitoring systems) and the military (770).

The concept of wearable sensors in sport is broad and includes any information gathering system that an athlete can wear/carry while participating in normal training/competition environments. Wearables can be worn by athletes on their person, in their clothing, or within their equipment and incorporate sensors, a microprocessor, and a form of communication unit that enables connectivity within a personal area network (PAN) where a smartphone or other appliance stores data and operates as a gateway with connectivity to the internet (771). Sensors and devices for athletes must be small and light, as well as flexible, durable, and impact resistant. Perhaps most importantly, any device should not negatively affect normal range of motion in an athlete’s chosen discipline. Simultaneously, wearables must produce precise measurements of biometrics like motion, heart rate, blood pressure, respiration rate, oxygen kinetics, blood, saliva, and sweat markers, and impact forces (772).

During the past decade, there has been a global explosion in wearables in sport and other health-related fields (772) underpinned by rapid developments in “smart technologies” such as artificial intelligence (AI) and machine learning. These technologies rely on sensor systems that collect, process, and transmit relevant data (such as biomarkers and other training/competition indexes) that are crucial to evaluate an athlete’s condition and maximize performance (771). In the sports setting, these platforms have several objectives: *1*) to gather valid, reliable, high-quality, data-rich sensory information from athletes in training/competition environments; *2*) to apply sophisticated analytics methods to identify patterns for determining athlete health and training/competition status; *3*) to obtain real-time data regarding training-related metrics (i.e., training variables, sleep quality, diet) while assisting athletes/coaches to manage a range of performance variables and outcomes to detect early signs and symptoms of overtraining (657); and *4*) to establish novel performance outcomes that supplement subjective, manually collected data and coach-based feedback with automated, objective data from devices. Data obtained from wearables can furthermore be leveraged to learn new techniques and provide real-time feedback on this process (770). For example, Samsung developed the Samsung SmartSuit to optimize body posture during short-track speed skating. The suit includes five integrated sensors and enables real-time feedback to the coach regarding the body position of the athlete: in this scenario, real-time feedback is transmitted to the athlete with instructions to modify body position to reduce drag and optimize performance. The Dutch short-tracker Suzanne Schulting used this suit to prepare for the Olympic Games in 2018 and became the first Dutch Olympic gold medal winner for 1,000 m, a feat she repeated in 2022.

Training load for sport performance encompasses both external and internal dimensions, with external training loads representing the physical work performed during a training session and internal loads being the associated biochemical and biomechanical stress responses. With regard to athlete monitoring, wearables provide information over four broad domains (771, 773): *1*) internal load, representing the psychophysiological responses to a given external load and typically determined by measurement of heart rate, oxygen uptake, blood lactate, and ratings of perceived exertion; *2*) external load, determined by the physical demands associated with a given stimulus, monitored by global navigation satellite systems, inertial measurement units, or linear/angular transducers that provide measures related to distance covered at certain velocities, acceleration/deceleration, and change of direction forces; *3*) well-being, monitored by subjective scales related to fatigue, DOMS, stress, quality of sleep, or mode state; and *4*) readiness, assessed by measures of heart rate variability, heart rate recovery, and variations in the test results of selected neuromuscular or submaximal/maximal test protocols. As long-term improvements in training adaptation and performance capacity are ultimately the result of an athlete’s cumulative internal load over multiple acute work bouts, the measurement of internal load and the factors influencing these outcomes is of paramount importance for the coach and athlete. A knowledge of the relationships between internal and external training loads has the potential to enhance training prescription, periodization, and athlete management through a detailed assessment of training fidelity and efficacy (773). Whether such information will provide coaches with an objective framework for evidence-based decisions remains to be determined. Moreover, potential issues with accuracy, dependability, or data security and privacy will have to be considered and solutions proposed that are acceptable for all stakeholders (774).

### 5.5. The Application of “-Omics” Technologies to Exercise Biology

In the past quarter century, the field of exercise biology has evolved to include sophisticated analysis of multi-tissues and organs through the application of established techniques already employed in other disciplines, as well as various -omics platforms to complement classic approaches (775, 776). Such inquiry has provided both a greater understanding of the biological bases of the health benefits of exercise (38) as well as knowledge of muscle bioenergetics and adaptation to training in recreationally active individuals and subelite athletes (55, 160, 777). The application of molecular techniques to exercise biology has provided novel insights into the complexity and breadth of intracellular signaling networks involved in response to both endurance- and resistance-based exercise (55, 161, 777). The recent explosion in global -omics technologies in the exercise sciences has also provided new opportunities to map the complexity and interconnectedness of biological networks underlying the tissue-specific responses and systemic benefits of exercise training (161, 455, 586, 778–782). A “sportomics” approach (the use of -omics sciences in sports) has even been proposed to complement existing methods of studying and monitoring an athlete’s state of fatigue and physical performance and aid in talent identification (783, 784). There have been in-depth and integrated multiomics profiling efforts of the response to acute exercise in subclinical populations (785), along with longitudinal “big data” approaches to develop prediction models for biomarkers for precision medicine (786). However, to date, few studies have been undertaken in elite athletic cohorts (787).

Although there is some evidence to suggest that a combined -omics solution will greatly facilitate discovery of the genetic and non-genetic influences on sporting performance, training response, injury predisposition, and other potential determinants of successful human performance (788), large-scale, collaborative efforts involving well-defined phenotypic cohorts will be essential for major progress to be made in the field of elite sport performance (782). Indeed, integration of data from multiple -omics approaches will require large sample sizes, big data sets, and expertise in computational biology to resolve the complex biology associated with the diverse exercise responses to endurance- and resistance-based training regimens. This will necessitate collaborative efforts between multiple research teams using common procedures and experimental protocols to execute multicenter exercise/lifestyle intervention trials with the goal of collecting sufficient functional and molecular data to further elucidate the mechanisms responsible for adaptive response to various exercise training regimes. Issues of data privacy and accessibility will have to be considered.

Such an approach is already underway: the Molecular Transducers of Physical Activity Consortium (MoTrPAC) is a multicenter study on the effects of two different forms of exercise (endurance and resistance training) across individuals of different ages and sexes as well as sedentary and well-trained individuals (782). There are two main aims of MoTrPAC: the first is to study the response to exercise at the whole body and cellular levels and to identify the molecular underpinnings that might be responsible for the adaptive process and variation among individuals. The second aim is to deliver a map of the biological molecules and pathways underlying the systemic effects of acute and chronic exercise (454, 782). Ultimately, the knowledge gained from MoTrPAC and other similar large-scale undertakings (e.g., the Wu Tsai Human Performance Alliance) will give biological science researchers and health professionals the insights to develop personalized training protocols to maximize performance and/or health benefits based on the unique molecular signatures and specific targets identified. Notwithstanding the increased knowledge that will accrue from a better understanding of these sophisticated biological processes and pathways, advances in training techniques for achieving new limits in human athletic performance have rarely had their origins in science. Part of the reason that sport science and exercise biology has failed to inform training practices stems from a reluctance of coaches to modify their methods, many of which have been nurtured and perfected over decades and are firmly entrenched as coaching “lore.” Donating tissue samples for exercise biologists to gain mechanistic insights into various training protocols has also met with limited success. As such, knowledge of training methods to enhance elite sport performance has traditionally evolved by way of trial-and-error observations of a few pioneering coaches and their athletes, with exercise scientists playing “post hoc” roles attempting to explain the underpinning biological mechanisms (99). Although major breakthroughs in the knowledge of how exercise activates numerous cellular, molecular, and biochemical pathways have been witnessed, direct evidence linking such effects to specific performance outcomes and understanding how these effects exert their benefits in different athletic populations remains elusive and a challenge for future research. To do so, exercise biologists who investigate training adaptation and elite athletic performance will have to integrate information pertaining to an athlete’s genetic and epigenetic background with tissue-specific gene expression, proteome, and metabolomic profiles to predict potential improvements in strength, aerobic capacity, and other traits necessary for elite performance.

## 6. SUMMARY, CONCLUSIONS, AND PERSPECTIVES

We stand on the shoulders of giants who have unraveled seminal and fundamental aspects of muscle biology and metabolism (789). Today, human studies are boosted by technological and conceptual advances that enable the investigation of molecular mechanisms, cellular functions, multicellular dynamics, as well as inter-tissue and inter-organ cross talk in an unprecedented manner. For example, the identification of myokines resulted in the new definition of skeletal muscle tissue as an endocrine organ, in fact the largest in our body (790, 791). Similarly, metabolism of kynurenine (792) and excess ketone bodies in hyperketonemia (575) in skeletal muscle, boosted by exercise, imply a role for this tissue in the detoxification of dysregulated endogenous metabolites, analogous to xenobiotic detoxification in the liver. scRNA-seq has yielded novel insights into the cellular composition of muscle, including the identification of previously unidentified cell types and the first analyses of multicellular dynamics and interactions (632–634). Likewise, single-nucleus RNA-seq (snRNA-seq) reveals a hitherto unsuspected coordination between nuclei in the same syncytial myofiber but also a surprising heterogeneity and subspecification of transcriptional programs that extends beyond the classically defined synaptic, extrasynaptic, and myotendinous nuclear populations (793–795), combined with insights into protein transfer between nuclei (650), RNA transport in the muscle fiber (651), and even the movement of myonuclei in specific contexts (649). Exon skipping, CRISPR-Cas9-based approaches, and adeno-associated viral (AAV) vectors have been applied in different settings and pathologies, including muscle diseases, in the preclinical setting, such as therapy of Duchenne muscular dystrophy (796). AAV-based gene therapy is now also used clinically, for example, in spinal muscular atrophy patients (797). Finally, progress is being made in the recognition of our increasingly sedentary lifestyle as a major risk factor for chronic metabolic diseases, the prescription of exercise-based interventions in the general population for preventing and/or treating an ever-increasing number of widespread conditions, and establishing physical activity as a cornerstone in medical practice and public health (26, 798, 799).

In elite sport, world records continue to be broken across a wide range of events. Performance improvements or declines depend on many factors, including technology, sports science, support for a particular sport, talent identification, investment of time, and effectiveness of training protocols (800–802). Advances in elite performance can also vary between individual events in one sport, as is the case for Olympic swimming competitions, with strong trends for improvement in some strokes and a relative plateau in others (803). With an increasing number of former Olympians and elite athletes now participating in masters competitions, age-group records are being surpassed and seemingly unattainable performances recorded such as the sub-3 hour marathon by a 70-yr-old man (804). Although big data approaches will be facilitated by the rapidly evolving tracking systems and wearables technologies combined with machine learning, AI, and other analysis methods (66, 805, 806), it is questionable as to what extent training practices of elite athletes have been facilitated by any major laboratory-based scientific breakthroughs to date. Indeed, despite our greater understanding of some of the mechanistic underpinnings of muscle plasticity and exercise adaptation, the upper limits of adaptation remain poorly studied (18). Even though numerous potential regulatory and functional key players have been identified, we do not know whether the picture is complete, how these factors are activated and engaged, how different pathways are integrated, or how the regulatory and functional outcome is specified, orchestrated, and coordinated. Moreover, the complexity of the apparent regulatory and functional redundancies, contingencies, alternatives, and adaptive mechanisms that ensure robust regulation of muscle plasticity as one of the most fundamental aspects of human life and evolution remains enigmatic (18). Our insights into the regulation of muscle plasticity in response to endurance-based training stimuli far surpasses that of resistance-based exercise, in part because of the availability of more robust or at least more commonly used animal models and protocols that are more physiological and translatable for the former training practice (807, 808). Compared to the muscle and whole body responses to an acute bout of exercise, the mechanistic aspects of chronic training-induced plasticity are less well investigated and understood. Similarly, the mechanistic understanding of the molecular bases of reduced training and/or detraining, as well as of retraining and muscle memory, remains rudimentary (433). Finally, we have limited knowledge of the dynamic multidirectional cross talk between muscle and other cell types, within and beyond muscle tissue, which is instrumental for adequate muscle function and delays a better delineation of training adaptation. For example, studies of the motor unit, the unity of muscle fiber and motor neuron, should include sensory-motor circuits in the spinal cord and supraspinal systems, brain stem neurons with descending axons, and brain regions that are involved in locomotor control and integrate sensory feedback, all important for muscle control, resistance training adaptation, muscle memory, and other processes (329, 456, 464, 809).

**FIGURE 21.**
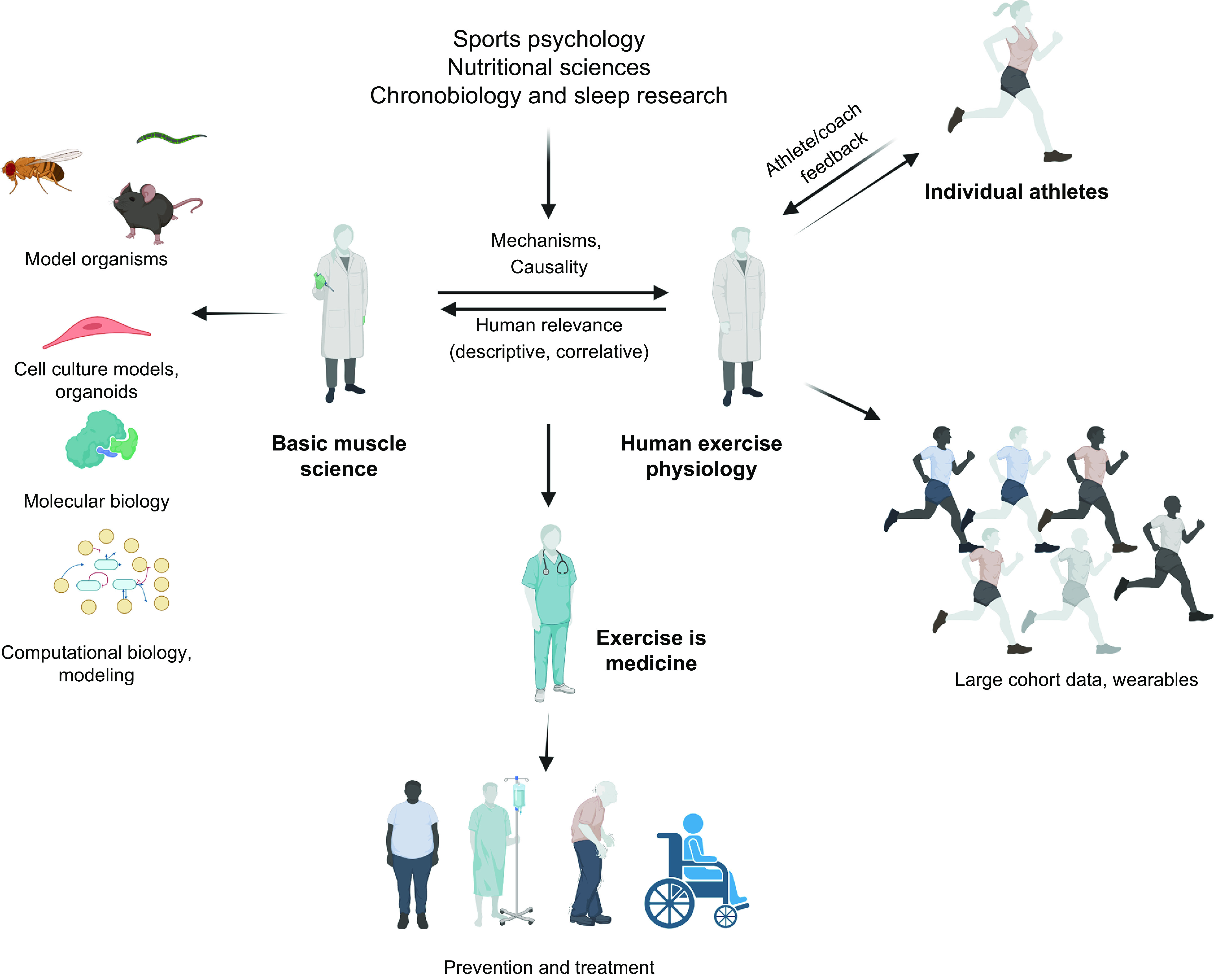
The future of exercise science for safe, evidence-based, and personalized approaches. To overcome existing hurdles and efficiently leverage the power of novel techniques and approaches, a close interaction between basic muscle research, applied human exercise physiology, as well as athletes and coaches should be aimed for. Model organisms, cell culture, and molecular and computational biology might provide insights into cause-effect relationships, epistasis, etiologies, and mechanisms complementing the descriptive and correlative studies in human volunteers. Inversely, data from large cohorts that will become available because of widespread use of wearables and trackers as well as those obtained in and based on feedback from athletes and coaches will reveal processes and pathways to be explored in mechanistic detail. The molecular athlete should furthermore be informed by sports psychology, e.g., in regard to motivation, perseverance, compliance and adherence, nutritional sciences, chronobiology, sleep research, and other fields of relevance to training. Finally, a mutual exchange between the observations in training and those in various pathologies associated with an inactive lifestyle or inadequate muscle functionality will help to push the boundaries of physical activity interventions in the prevention and treatment of numerous diseases. Image created with BioRender.com, with permission.

How will we overcome these shortcomings, and what could the future of muscle research look like? In addition to new advances in technological possibilities, analysis methods, and computational modeling approaches, one aim would be stronger interaction, collaboration, and networking between basic science, sports sciences, and coaches as well as athletes (FIGURE 21). All these fields, when optimally synergized, may help to obtain a better understanding of muscle plasticity from the inactive to the extreme (810, 811). Currently, training paradigms pioneered by athletes tend to inform and guide research, as was the case for interval training practiced by Paavo Nurmi in the 1920s and Emil Zátopek in the 1950s for middle- and long-distance running disciplines (812). Such innovative methods are currently enjoying intense scientific scrutiny in the refined form of HIIT, for both athletic endeavors and the fitness and well-being of the general population (100). Athlete feedback is central to understanding individualized training response, fatigue recovery, or concurrent training design and helps to study the mechanistic aspects that underlie these processes for iterative optimization of training and competition (71). Importantly, sports psychology and neurobiology should integrate morphological, cellular, and mechanistic aspects to identify the relevant circuitries, regions, and signals involved in the control of these factors, including the cross talk between muscle and brain (494, 813, 814).

Bypassing this chain of research by jumping directly from basic science to athletes often leads to a mismatch between preclinical data and “real-world” performance. For example, based on mechanistic insights and mouse experiments with so-called “exercise mimetics,” some coaches and athletes experimented with performance-enhancing compounds, most recently AMPK and PPARβ/δ activators, without waiting for robust scientific validation from human trials (45, 46). Not only is there no evidence for performance enhancement in humans (815), but in some cases (e.g., metformin, resveratrol, and rapamycin), there may be a reduction of training adaptation (45, 46). Moreover, such compounds may have a significant risk of severe adverse effects that might not be relevant in the time frame of application and life expectancy in rodents but could have detrimental long-term effects in humans (45, 46). For example, prolonged, sustained activation of AMPK could lead to a catabolic state, lactic acidosis, cardiac hypertrophy, brain inflammation, and reduced cognitive abilities (45, 46, 816). PPARβ/δ ligands increase the risk of tumors in rodents when given at high doses over a prolonged period of time, and even AMPK’s action can switch from tumor suppressor to tumor promoter once cancer develops (45, 46, 817). It is encouraging to see that integrative approaches are increasingly pursued at different centers and organizations in which elite athlete training and health management is under the same roof as integrative research. Thus, collectively, mechanistic understanding, implementation in training design, technological innovations, and other advances, cross-fertilized with data from psychology, nutrition, and sleep research, will help to further optimize athletic performance in a safe, personalized, and evidence-based approach (818, 819). Thereby, pseudoscience, baseless claims, “quick fixes,” and other potentially detrimental interventions can be minimized and information separated from misinformation (820). Finally, integrative research in exercise should be combined with exercise medicine to further our understanding of the immense potential of exercise-based interventions to prevent and treat many chronic diseases in the general population (821), along with the use of training for injury rehabilitation in physical therapy. A better understanding in the athletic setting thus could and should inform interventions in the general population and in patients (822). For example, studies of resilience and motivation might also help to design approaches to facilitate and improve adherence and compliance to exercise training (823–825). Such an approach could leverage novel avenues such as virtual reality exergames (826, 827). Insights into exercise physiology are leveraged in disease diagnostic, prevention, treatment, and rehabilitation, e.g., the use of blood pressure measurements during exercise (exercise hypertension) to reveal undiagnosed or masked hypertension and predict cardiovascular disease risks (828, 829). Many more areas exist in which concepts derived from elite sports will benefit non-athletes and patients (822). In this context, exercise sociology could contribute to overcome existing individual and societal challenges (830). Hopefully, exercise will firmly be recognized and accepted in all aspects of clinical practice and the political and societal framework established to facilitate and promote an active lifestyle.

Finally, an issue that will have to be addressed in the field of biological/sports science is the formulation of hypotheses and interpretation of data and results. Too often, papers claim to have solved an open question in an absolute manner, even though in many cases seemingly conflicting, contradictory, or non-overlapping alternative studies exist. Often, such studies depend on relatively small subject numbers and might have to be interpreted in light of age, sex, training protocol, sampling time, and other parameters (diet, sleep, comorbidities, fitness level, chronotype, and time of day of the study) (831). Human and animal studies are guilty of this alike. Studies in model organisms have a limited predictive power for human exercise physiology but enable causative and mechanistic insights, which are difficult to conduct in human trials that rely mainly on descriptive or correlative data. Moreover, while model organisms provide access to all different types of muscle beds and allow the analysis of whole muscles, human studies are in most cases limited to small, single biopsies of one muscle (typically the vastus lateralis), taken at different times before, during, or after exercise, with considerable variability in outcomes and hence interpretation of results (388, 446, 832). It might be advisable to be aware of possible limitations and keep an open mind vis-à-vis alternative interpretations, study-specific bias, and system complexity surpassing simplistic explanations. For example, there most likely is more than one cause for muscle fatigue (833); satellite cell recruitment might be important for hypertrophy in some but not all cases (658); the relative contribution of training intensity and volume on mitochondrial function in muscle might be very context-dependent (104, 105) as is the relative contribution of muscle hypertrophy to gains in strength (834, 835); and polarized or pyramidal intensity distributions might be optimal for performance enhancement (116, 132–134). Even “established principles,” “dogmas,” and “laws” should constantly be questioned, validated, and refined. Unfortunately, more often than not, literature searches for such findings disappear into a trail of never-ending, consecutive citations. In addition, data and the arising hypotheses must be examined under consideration of the technical and conceptual possibilities of the respective historical time period. For example, Henneman’s size principle of motor unit recruitment (836) certainly holds up under the laboratory conditions using the exact preparations and the methods that were used in the 1950s to 1970s. However, the rigid view of this principle has since been refined and modified with novel approaches (i.e., using more complex musculotendon-skeletal systems, physiologically relevant range of forces, physiological and neural stimulation as opposed to electromyography and -physiology, or consideration of neural drive, cortical and afferent input to each individual motor neuron) (319, 321, 330, 625). Moreover, it is not clear how the size principle can accommodate muscle fiber type shifts in exercise (624), and recent scRNA-seq and snRNA-seq approaches revealed a greater diversity in motor neuron populations than the classically defined types based on transcriptional profiles (326, 837). Thus, even though probably correct at its core, the size principle might be oversimplified, and motor unit recruitment and plasticity certainly warrant further study. Similarly, even though Nobel prize worthy, August Krogh’s ideas on oxygen delivery and muscle microvasculature have, for some aspects, not withstood the test of time or have been refined and altered by more modern and comprehensive methods (595). The myonuclear domain hypothesis was postulated to account for the syncytial nature of myofibers, implying that a nucleus is needed for adequate support for transcription and translation within a specific cell volume, hence a fixed myonuclear domain, in these extraordinarily large cells (838). Accordingly, satellite cell recruitment would be needed to provide additional nuclei in fiber hypertrophy (839). However, the myonuclear domain hypothesis fails to provide an adequate and complete explanation for several observations. First, removal of myonuclei in atrophy is controversial and not observed consistently (839–843). Second, the myonuclear domain has high flexibility and scales with body size, fiber type, mitochondrial activity, fiber hypertrophy, and other parameters (838, 844–846). Third, even though the spacing between most myonuclei is roughly even, regional differences exist, most notably in the tight clusters of three to five subsynaptic myonuclei at the NMJ and similar clusters at the myotendinous junction, for both of which one nucleus should theoretically be sufficient to serve the respective cytoplasmic domain (468). Ample evidence exists of intracellular movement of myonuclei (847), as in postexercise fiber repair (649), exchange of proteins between nuclei (650), and microtubule-mediated transport of ribonucleoproteins and RNAs within the myofiber (651), all of which imply a highly plastic system transcending a more rigid definition of myonuclear domains. Finally, in cells that rival myofibers in terms of size or length, such as certain motor neurons with an axonal length of >1 m (848) compared with some of the longest muscle fibers in the human musculus sartorius reaching the length of ∼60 cm (849), one nucleus, located asymmetrically in the cell body in the spinal cord, seems sufficient to provide transcripts for the whole cell. Thus, the evolutionary pressure for and physiological function of the syncytial nature of myofibers remain largely mysterious.

These are just a few examples to illustrate that in exercise biology and sport science, as in any place of scientific and social discourse (850–852), we should have passionate arguments but remain fair, civil, agnostic, and open minded, carefully consider alternative results and hypotheses, and constantly challenge, validate, and refine (or refute!) seemingly “established” principles. In the final analyses, modern sports science and exercise biology offer numerous opportunities to assist elite athletes to refine training methods, optimize adaptation, stay healthy and injury free, achieve their desired physique, and fight against fatigue factors that limit successful performance. The accomplishments of elite athletes will continue to entertain and amaze us, as science attempts to catch up and explain the biological bases of such feats.

## GRANTS

Work in the laboratory of R.F. and C.H. related to this article was supported by the Swiss National Science Foundation (310030_184832), the European Research Council (616830-MUSCLE_NET), Innosuisse (44112.1 IP-LS), the Swiss Society for Research on Muscle Diseases, the Jain Foundation, the Biozentrum, and the University of Basel.

## DISCLOSURES

C.H. is an associate editor of *Physiological Reviews* and was not involved in and did not have access to information regarding the peer-review process or final disposition of this article. An alternate editor oversaw the peer-review and decision-making process for this article. None of the other authors has any conflicts of interest, financial or otherwise, to disclose.

## AUTHOR CONTRIBUTIONS

R.F., J.A.H., and C.H. prepared figures; drafted manuscript; edited and revised manuscript; and approved the final version of the manuscript.

## GLOSSARY

### Abbreviations

AMPK: AMP-dependent protein kinase
ATP: Adenosine triphosphate
CSA: Cross-sectional area
DOMS: Delayed-onset muscle soreness
ECM: Extracellular matrix
EPO: Erythropoietin
Hb: Hemoglobin
HIF-1α: Hypoxia-inducible factor 1α
HIIT: High-intensity interval training
IL: Interleukin
MPS: Muscle protein synthesis
mTOR: Mammalian target of rapamycin
mTORC1: mTOR complex 1
NMJ: Neuromuscular junction
OXPHOS: Oxidative phosphorylation
PGC-1α: Peroxisome proliferator-activated receptor γ coactivator 1α (gene name PPARGC1A)
1RM: One-repetition maximum
RYR: Ryanodine receptor
SERCA: Sarcoplasmic/endoplasmic reticulum Ca2^+^-ATPase
SNP: Single-nucleotide polymorphism
SR: Sarcoplasmic reticulum
VEGF: Vascular epithelial growth factor
$\dot{\text{V}}{\text{O}}_{2\max}$: Maximal oxygen uptake
